# Supplementary material for: A double-blind, placebo-controlled, randomized, multi-centre, phase III study of MLC901 (NeuroAiDTMII) for the treatment of cognitive impairment after mild traumatic brain injury
Source: PLoS One. 2025 Jul 10;20(7):e0310229. doi: 10.1371/journal.pone.0310229 (PMC12244563; doi:10.1371/journal.pone.0310229)
Supplement: S1 File — (PDF) [file pone.0310229.s001.pdf]

CLINICAL STUDY PROTOCOL

**Protocol Number**	#EFSA2020_03
**Protocol Version, Date**	Version 3.0 dated 08-02-2022
**Protocol Approval Date**	08-02-2022
**Study Title**	A randomized double-blind, placebo-controlled, multi-center trial to investigate the efficacy and safety of NeuroAiD II™ (MLC901) to improve cognitive functioning in non-surgical mild traumatic brain injury patients
**Study Acronym**	**SAMURAI (**a randomized double-blind controlled trial to investigate the **S**afety and effic**A**cy of **M**LC901 in cognitive recovery post tra**U**matic B**RA**in **I**njury)
**Study Phase**	PHASE III
**Investigational Product**	NeuroAiD II™ (MLC901)
**Sponsor**	Moleac Pte Ltd, Helios #09-08, 11 Biopolis Way, Singapore 138667
**The name of the legal entity, authorized by the Sponsor to organize and conduct clinical study**	Contract Research Organization<br>Atlant Clinical Ltd<br>12 Krivokolennyi per., Building 1, Moscow, 101000, Russian Federation<br>Tel.: +7 (495) 628 3802<br>Fax: +7 (495) 628 3633

Version History

**Date**	**Version Number**
28-12-2020	v. 1.0 - Initial Protocol
08-04-2021	v. 2.0
08-02-2022	v. 3.0

CONFIDENTIAL

This document contains confidential information. This information is intended for persons responsible for the carrying out and the organization of the study and can be provided to them under the condition of the consent of these persons on the further non-distribution of this information.

SIGNATURES

Sponsor's Representative

Typed Name	Htar Htar Nwe
Title	Senior Medical Advisor
Signature	Date: 2022.06.16 16:08:04 +02'00'
Date signed	

Contract Research Organization's Representative

Typed Name	Elena Dementyeva
Title	Project Management Director
Signature	
Date signed	

INVESTIGATOR PROTOCOL AGREEMENT PAGE

I, the undersigned, am responsible for the conduct of the study at this site and agree to the following:

- I understand and will conduct the study according to the protocol, any approved protocol amendments, ICH GCP and all applicable regulatory authority requirements and national laws of Russian Federation in the field of drug circulation.
- I will not deviate from the protocol without prior written permission from the Sponsor and prior review and written approval from the Institutional Review Board or Independent Ethics Committee, except where necessary to prevent any immediate danger to the subject.
- I have sufficient time to properly conduct and complete the trial within the agreed trial period, and I have available an adequate number of qualified staff and adequate facilities for the foreseen duration of the trial to conduct the trial properly and safely.
- I will ensure that any staff at my site(s) who are involved in the study conduct are adequately trained regarding the protocol and their responsibilities. In the case of delegating any of my study responsibilities I will provide the Sponsor with a Delegation of Activities certificate.
- I understand that some regulatory authorities require Sponsors of clinical studies to obtain and supply, when required, details about the Investigators' ownership interests in the Sponsor or the Investigational Medicinal Product and information regarding any financial ties with the Sponsor. The Sponsor will use any such information that is collected solely for the purpose of complying with regulatory requirements. I therefore agree to supply the Sponsor with any necessary information regarding ownership interest and financial ties (including those of my spouse and dependent children), and to provide updates as necessary.

Typed Name	
Title	
Study site	
Signature	
Date signed	

TABLE OF CONTENTS

SIGNATURES 2
TABLE OF CONTENTS 4
1 GENERAL INFORMATION 10
1.1 Contacts 10
1.2 Protocol synopsis 12
1.3 Study Schedule 21
2 BACKGROUND INFORMATION 23
2.1 Rationale 23
2.2 Name and Description of the Investigational Product 23
2.2.1 Description and Composition of the Drug Product 25
2.3 Summary of Findings from Nonclinical Studies that Potentially have Clinical Significance and from Clinical Studies that are Relevant to the Study 26
2.3.1 Nonclinical studies 26
2.3.2 Clinical studies 27
2.4 Summary of the Known and Potential Risks and Benefits, if any, to Human Subjects 31
2.5 Description of and Justification for the Route of Administration, Dosage, Dosage Regimen, and Treatment Period 32
2.6 Regulatory Requirements to be Considered During Study Conduction 34
2.7 Description of the Population to be Studied 35
2.8 Critical processes and data identification 35
2.9 References to Literature and Data that are Relevant to the Study, and that Provide Background for the Study 35
3 STUDY AIM, OBJECTIVES AND ENDPOINTS 36
3.1 Study aim 36
3.2 Objectives and endpoints 36
3.2.1 Efficacy 36
3.2.2 Safety 37
4 STUDY DESIGN 38
4.1 Description of Study Design 38
4.1.1 Study procedures 39
4.2 Description of the Measures Taken to Minimize/Avoid Bias 43
4.2.1 Randomization 43
4.2.2 Blinding 43
4.3 Description of the Study Treatments 43

4.4 Expected Duration of Subject Participation, and a Description of the Sequence and Duration of All Study Periods, Including Follow-up 44

4.5 Description of the "Stopping Rules" or "Discontinuation Criteria" for Individual Subjects, Parts of Study and Entire Study 44

4.6 Accountability Procedures for the Investigational Product(s), Including the Placebo and Comparator 44

4.7 Maintenance of Study Treatment Randomization Codes and Procedures for Breaking Codes 45

4.8 Identification of Any Data to be Recorded Directly on the Electronic CRF (eCRFs) ...45

5 SELECTION AND WITHDRAWAL OF SUBJECTS 46

5.1 Subject Inclusion Criteria 46

5.2 Subject Exclusion Criteria 46

5.3 Contraception 47

5.4 Subject Withdrawal Criteria 47

5.5 Replacement of the Subjects 48

5.6 Follow-up of the Withdrawn Patients 49

5.7 Strategies for Recruitment and Retention 49

5.7.1 Recruitment 49

5.7.2 Retainment 49

6 TREATMENT OF SUBJECTS 50

6.1 Treatment(s) to be Administered 50

6.1.1 Investigational Product 50

6.1.2 Placebo 52

6.2 Medications/Treatments Permitted (Including Rescue Medication) and Not Permitted Before and/or During the Study 52

6.3 Procedures for Monitoring Subject Compliance 53

7 ASSESSMENT OF EFFICACY 54

7.1 Specification of the Efficacy Parameters 54

7.2 Methods and Timing for Assessing, Recording, and Analyzing of Efficacy Parameters 54

8 ASSESSMENT OF SAFETY 58

8.1 Specification of Safety Parameters 58

8.2 Methods and Timing for Assessing, Recording, and Analyzing Safety Parameters 58

8.3 Procedures for Eliciting Reports of and for Recording and Reporting Adverse Event and Concurrent Illnesses 58

8.3.1 Definitions 59

8.3.2 Evaluation of severity 60

8.3.3 Causality assessment 60
8.3.4 Documentation and reporting of adverse events 61
8.3.1 Reporting Adverse Events to Subjects 62
8.3.2 Responsibility of the Sponsor....63
8.4 Type and Duration of the Follow-up of Subjects After Adverse Events....63
8.5 Safety data review by DSMB 63
9 STATISTICS....65
9.1 Description of the Statistical Methods to be Employed....65
9.1.1 Descriptive analysis of demography and baseline data....65
9.1.2 Efficacy analysis....65
9.1.3 Safety analysis 66
9.2 Number of Subjects Planned to be Enrolled....67
9.3 Level of Significance to be Used....68
9.4 Criteria for the Termination of the Study 68
9.5 Procedure for Accounting for Missing, Unused, and Spurious Data....68
9.6 Procedures for Reporting Any Deviation(s) from the Original Statistical Plan 68
9.7 Selection of Subjects to be Included in the Analyses 68
10 DIRECT ACCESS TO SOURCE DATA/DOCUMENTS 69
11 QUALITY CONTROL AND QUALITY ASSURANCE 70
11.1 Site Monitoring Visits....70
11.2 Protocol deviations 70
11.3 Quality Assurance Audits and Regulatory Agency Inspections....70
11.4 Data Monitoring and Quality Control....70
11.5 Steering Committee 71
11.6 Data Safety Monitoring Board (DSMB)....72
12 ETHICS 74
12.1 Approval by Ethics Committee....74
12.2 Subject Information and Informed Consent....74
12.3 Confidentiality of patient data 75
13 DATA HANDLING AND RECORD KEEPING....76
14 FINANCING AND INSURANCE....77
15 PUBLICATION POLICY 78
16 REFERENCES....79
17 APPENDICES....82

17.1 Glasgow Coma Scale 82
18 AMENDMENTS TO THE PROTOCOL 83

TABLES

Table 1.1. Schedule of Study Procedures 21
Table 2.1. Name and Description of the Investigational Product 23
Table 2.2. Composition of MLC901. 25
Table 2.3. Number of patients experiencing medical events over 24 months in the CHIMES-E Study (44,45) 30
Table 3.1. Study efficacy objectives and endpoints. 36
Table 3.2. Study safety outcomes and endpoints 38
Table 4.1. Study treatments. 43
Table 5.1. Acceptable Effective Methods of Contraception 47
Table 6.1. Description of IP. 50
Table 6.2. Composition of MLC901. 51
Table 6.3. Description of Placebo. 52
Table 7.1. Efficacy outcomes, assessment methods and terms. 54
Table 7.2. Outcome measures. 55
Table 9.1. Complex Attention score: Mean (SD) (extract from the Supplementary Table 1 in (21) 67

FIGURES

Figure 4.1. Study design. 39

LIST OF ABBREVIATIONS

ADR	Adverse drug reaction
AE	Adverse Event
ALT	alanine aminotransferase
AR	Adverse reaction
AST	aspartate aminotransferase
BDNF	Brain-derived neurotrophic factor
BI	Barthel Index
BMI	Body Mass Index
BP	Blood pressure
CTCAE	Common Terminology Criteria for Adverse Events
CFQ	Cognitive Failures Questionnaire
CFR	Case report form
CNS-VS	Central Nervous System Vital Signs
CRF	Case report form
ECG	Electrocardiogram
DSMB	Data safety monitoring board
GCP	Good Clinical Practice
GOS-E	The Glasgow Outcome Scale Extended
GSC	Glasgow Coma Scale
HADS	The hospital Anxiety and Depression Scale
HPMC	Hydroxypropyl methylcellulose
HR	Heart rate
IB	Investigator's Brochure
ICF	Informed consent form
ICH	International Conference on Harmonization
IP	Investigational Product
ITT	Intent-to-Treat
IWRS	Interactive Web Response System
LOCF	Last Observation Carry-Forward
LEC	Local Ethical Committee
mg	milligram
min	minute
MMSE	Mini-Mental Status Examination

mTBI	Mild traumatic brain injury
NIHSS	National Institutes of Health Stroke Scale
PI	Principal Investigator
PP	Per-Protocol
PVDC/PE/PVC	Polyvinylidene Chloride/ Polyethylene/Polyvinyl chloride
QOLIBRI	Quality of Life after Brain Injury
SAE	Serious adverse event
SC	Steering Committee
SUSAR	Suspected unexpected serious adverse reaction
TBI	Traumatic brain injury
TEAE	Treatment-emergent adverse event
WOCBP	Women of childbearing potential

1 GENERAL INFORMATION

1.1 Contacts

Sponsor's contact details

**Moleac Pte Ltd**<br>Sponsor Legal Registered Address/ Contact Address: Helios #09-08, 11 Biopolis Way, Singapore 138667
**Senior Medical Advisor**<br>Htar Htar Nwe, MBBS<br>Tel.: +65 6211 3710 Ext 182<br>Tel. (mobile): +65 8533 0651<br>E-mail: htarhtar.nwe@moleac.com
**Sponsor's medical expert**:<br>Senior Medical Advisor<br>Htar Htar Nwe, MBBS<br>Tel.: +65 6211 3710 Ext 182<br>Tel. (mobile): +65 8533 0651<br>E-mail: htarhtar.nwe@moleac.com
**Sponsor Serious Adverse Events (SAE) Coordinator**:<br>Senior Medical Advisor<br>Htar Htar Nwe, MBBS<br>Tel.: +65 6211 3710 Ext 182<br>Tel. (mobile): +65 8533 0651<br>E-mail: htarhtar.nwe@moleac.com

The legal entity, authorized by the Sponsor to organize and conduct clinical study, contact details:

Contract Research Organization Atlant Clinical, Ltd

Address: 12 Krivokolennyi per., Building 1, Moscow, 101000, Russian Federation

Tel.: +7 (495) 628 3802

Fax: +7 (495) 628 3633

Project Management Director

Elena Dementyeva

Tel.: +7 (495) 628 3802 ext.211

Tel. (mobile): +7 (929) 963 6209

E-mail: elena.dementyeva@atlantclinical.com

The list of study centers and Investigators that are involved in this clinical study (name, address of the center, name of the PI) will be provided separately.

Only local laboratories will be involved in the trial.

1.2 Protocol synopsis

Sponsor	Moleac Pte Ltd<br>Helios #09-08, 11 Biopolis Way, Singapore 138667
Study Title	A randomized double-blind, placebo-controlled, multi-center trial to investigate the efficacy and safety of NeuroAiD II™ (MLC901) to improve cognitive functioning in non-surgical mild traumatic brain injury patients (SAMURAI)
Protocol Number	#EFSA2020_03
Study Phase	PHASE III
Study type	Randomized, double-blind, placebo-controlled, multi-center trial
Investigational Product and dosing	NeuroAiD II™ (MLC901), 2 capsules 3 times a day
Route of Administration	Oral administration
Administration conditions	MLC901 capsules could be:<br>- swallowed as such with water<br>- opened and drunk once powder has been diluted in a sufficient amount of water of room temperature *<br>**Clumping of the powder content may be observed due to the inherent nature of the powder extract. This does not indicate deterioration of quality if used within the specified shelf life.*
Study population	Adult patients aged 18-65 years with long-term cognitive impairment following mild traumatic brain injury (mTBI)
Number of subjects	182 patients
Subject participation duration	No more than 314 days for each patient
Study Purpose:<br><br><br><br>Study Objectives:	To determine the efficacy of NeuroAiD II™ (MLC901) in improvement of cognitive functioning of adult patients with long-term cognitive impairment following mild traumatic brain injury (mTBI) and to assess safety of NeuroAiD™ (MLC901) in these patients.<br><br>**Efficacy**<br>1. To determine the effect of NeuroAiD II™ (MLC901) on changes in:<br>• complex attention,<br>• other cognitive domains: executive functioning, processing speed, memory (visual and verbal), reaction time<br>• post-concussion symptoms<br>• quality of life<br>• levels of anxiety and depression<br>in adult patients with long-term cognitive impairment following mTBI compared to baseline after 6 months of NeuroAiD II™ (MLC901) treatment with reference to placebo-treated adult patients with long-term cognitive impairment following mTBI.

	2. In case of any improvement in any of the above parameters upon completion of NeuroAiD II™ (MLC901) treatment: to assess changes in improved parameters 3 months after treatment completion with reference to the placebo-treated group.<br><br>**Safety**<br>The objective of the study is to assess safety of NeuroAiD II™ (MLC901) compared to placebo, following a course of 6-month treatment in adult patients with mTBI.
Study Design:	This is a randomized double blind, placebo-controlled, multi-center clinical trial. This clinical trial will include 182 adults (aged between 18 and 65 years of age) who experienced a mild TBI in the past 1 to 12 months and experience cognitive functioning difficulties as indicated by a score of >30 on the Cognitive Failures Questionnaire. Eligible participants will be randomized to receive either NeuroAiD II™ (MLC901) or matching placebo.<br><br>The study will consist of 3 periods:<br>• **Screening** (Day -14…-1)<br>• **Treatment period** (6 months, Day 1 – Day 180±14), during which patients will daily receive an oral dose of IP or placebo<br>• **Follow-up period** (3 months, Day 180±14 – Day 270±30), after which final efficacy assessment will be performed<br><br>The time period from Screening (Visit 1) to Visit 2 (Day 1, Randomization) will not exceed 14 days. If the patient leaves the study before study completion for any reason, then the Early-Termination visit is performed, which should include all procedures and assessments, planned for the End-of-Study visit on Day 270±30.
Study Schedule and Procedures	Written Informed Consent will be signed prior to any study procedures commencement.<br><br>Screening procedure (Visit 1, Day -14…-1)<br><br>• The eligibility screening will include completion of the Cognitive Failures Questionnaire (CFQ). A score of >30 will be used to determine if the participant is experiencing cognitive impairment. CFQ will be performed after signing ICF at screening visit.<br>• If the subject has a CFQ score >30 and is ready to participate in the study, then the Investigator should check if the subject fulfils all other Inclusion and Exclusion Criteria.<br>• Collection of medical history, prior/concomitant medication, demographic and anthropometric data, date/time of TBI and injury characteristics including worst recorded GCS score.<br>• Pregnancy test<br>• Assessment of vital signs<br>• Physical examination

	• Clinical Laboratory Tests<br>• ECG<br>• A meeting with the Investigator will be arranged for baseline assessment and randomization.<br><br>**Treatment period**<br><u>Randomization assessment (Visit 2, Day 1)</u><br>• Randomization (IP/Placebo)<br>• Collection of baseline characteristics, medical history, prior/concomitant medication<br>• Efficacy outcomes to be assessed:<br>− Cognitive functioning (complex attention, executive functioning, processing speed, memory (visual and verbal), reaction time) assessed with CNS-Vital Signs (CNS-VS)<br>− Rivermead Post Concussion Symptoms Questionnaire (RPQ) - Questionnaire for Post-concussion symptoms<br>− Health related quality of life (QOLIBRI) – Questionnaire for assessment of quality of life<br>− Hospital Anxiety and Depression Scale (HADS) – Questionnaire for mood assessment<br><br>• Safety assessments:<br>- Vital Signs<br>- Physical examination<br>- Collection of information about AEs<br>• Investigational product supply<br><br><u>Assessments after 1 and 3 months of treatment (Days 30±7 (Visit 3), 90±7 (Visit 4))</u><br>• Efficacy outcomes to be assessed:<br>− CNS-Vital Signs (CNS-VS) - Computerized assessment for Cognition<br>− Rivermead Post Concussion Symptoms Questionnaire (RPQ) -Questionnaire for assessment of post-concussion symptoms<br>− Health related quality of life (QOLIBRI) – Questionnaire for assessment of quality of life<br>− Hospital Anxiety and Depression Scale (HADS) – Questionnaire for mood assessment<br><br>• Safety assessments:<br>− Vital Signs<br>− Physical examination<br>− Information about AEs<br><br>• Collection of prior/concomitant medication

<table>
<tr><td></td><td>
Compliance assessment
Investigational product supply
<u>Assessment after 6 months of treatment (Day 180±14, Visit 5)</u>
Efficacy outcomes to be assessed:
CNS-Vital Signs (CNS-VS) - Computerized assessment for Cognition
Rivermead Post Concussion Symptoms Questionnaire (RPQ) -Questionnaire for assessment of post-concussion symptoms
Health related quality of life (QOLIBRI) – Questionnaire for assessment of quality of life
Hospital Anxiety and Depression Scale (HADS) – Questionnaire for mood assessment
Safety assessments:
Vital Signs
Laboratory tests
Physical examination
Information about AEs
ECG
Pregnancy test
<u>Collection of prior/concomitant medication</u>
<u>Compliance assessment</u>
Follow-up period
<u>Assessment 3 months after treatment completion (End-of-Study, Visit 6, Day 270±30)</u>
Efficacy outcomes to be assessed:
CNS-Vital Signs (CNS-VS) - Computerized assessment for Cognition
Rivermead Post Concussion Symptoms Questionnaire (RPQ) -Questionnaire for assessment of post-concussion symptoms
Health related quality of life (QOLIBRI) – Questionnaire for assessment of quality of life
Hospital Anxiety and Depression Scale (HADS) – Questionnaire for mood assessment
Safety assessments:
Vital Signs
Physical examination
Information about AEs
Collection of prior/concomitant medication
Compliance assessment
</td></tr>
<tr><td>Efficacy endpoints</td><td>Primary efficacy endpoint</td></tr>
</table>

	Change in complex attention score, determined using Central Nervous System Vital Signs (CNS-VS) computer cognitive testing system, after 6 months of treatment compared to baseline in the group of patients receiving NeuroAiD II™ (MLC901), compared to the placebo group.<br>**Secondary efficacy endpoints**<br>• Changes in the scores for the following cognitive domains: executive functioning, processing speed, memory (visual and verbal) and reaction time, determined by CNS-VS system, after 6 months of treatment compared to baseline, in the NeuroAiD II™ (MLC901) group, compared to the placebo group.<br>• Change in the Rivermead Post Concussion Symptoms Questionnaire (RPQ) total score, after 6 months of treatment, compared to baseline, in the NeuroAiD II™ (MLC901) group compared to the placebo group.
Exploratory endpoints	• Change in the Health-related quality of life questionnaire (QOLIBRI) total score after 6 months of treatment, compared to baseline, the NeuroAiD II™ (MLC901) group compared to the placebo group.<br>• Change in the Hospital Anxiety and Depression Scale (HADS) score after 6 months of treatment, compared to baseline, in the NeuroAiD II™ (MLC901) group compared to the placebo group.<br>• Changes, compared to baseline, in the analyzed parameters (complex attention, executive functioning, processing speed, visual and verbal memory, reaction time, RPQ score, QOLIBRI score, HADS score) 9 months after NeuroAiD II™ (MLC901) treatment initiation with reference to baseline, compared to the placebo group.
Safety endpoints	• Number of TEAEs, recorded during the study period (270±30 days) in the NeuroAiD II™ group and in the Placebo group<br>• Number of ADRs, recorded during the study period (270±30 days, from Day 1 up to End-of-Study visit) in the NeuroAiD II™ group and in the Placebo group<br>• Number of SAEs, recorded during the study period (270±30 days) in the NeuroAiD II™ group and in the Placebo group<br>• Number of patients who discontinued treatment in the NeuroAiD II™ group and in the Placebo group<br>o Due to AE<br>o For any reason<br>• Number of clinically significant (determined by the Investigator) deviations in the results of hematology, blood chemistry and urine laboratory tests upon NeuroAiD II™/Placebo treatment completion (Day 180±14) in the NeuroAiD II™ group and in the Placebo group compared to baseline
Inclusion Criteria	1. Patient with mild TBI, which occurred 1-12 months prior enrolment to the study. Mild TBI is defined as an external force from an incident causing injury to the brain and resulting in an

	altered level of consciousness. The mTBI diagnosis should be documented. Mild TBI is evidenced by any of the following:<br>• best Glasgow Coma Score 13-15 as assessed on scene, on admission and over next 3 days<br>• loss of consciousness for up to 30 minutes<br>• dazed and confused at the time of injury or post-TBI amnesia of < 24 hours duration.<br>2. Patient experiencing cognitive impairment following injury, determined by the Cognitive Failures Questionnaire score >30.<br>3. Adult male or female patients aged 18-65 years.<br>4. The patient has signed an Informed Consent form (ICF) for participation in this study before initiation of study procedures.<br>5. The patient can understand all protocol requirements, perform the study procedures, and agree to all limitations specified in the protocol.<br>6. The patient agrees to use adequate contraception methods.
Exclusion Criteria	1. Moderate or Severe TBI, determined by best Glasgow Coma Score of <13 (as assessed on scene, on admission and over next 3 days), or injury requiring neurosurgery (even if surgery was not done)<br>2. Co-existing severe co-morbidity, including end stage renal failure, spinal cord injury, significant substance abuse, severe liver disease, significant mental illnesses, diabetes requiring insulin injections, severe agitation, advanced cancer or other severe conditions with life expectancy of less than 5 years. The decision on significance of the comorbidity for inclusion/exclusion from current study is made by the Investigator.<br>3. Current participation in another clinical trial within 30 days.<br>4. Women who are pregnant or who have a positive urine pregnancy test or breast-feeding.<br>5. Not fluent in Russian language or have aphasia/dysphasia.<br>6. No documented evidence of mTBI.<br>7. Known allergy to MLC901/NeuroAiD II™ or any of its component ingredients.<br>8. Other medical condition which in the opinion of the Principal Investigator would place undue risk on the patient if included in the trial or likely to interfere with NeuroAiD II™.<br>9. History of psychic (including depressive) disorders, physical and other factors that do not allow for adequate self-assessment of

	one's behaviour and for compliance with the protocol requirements, including history of psychiatric disorders.<br>10. Use of hormonal contraceptives, either oral or implant*.<br>*Subjects and their sexual partner must use an acceptable single- or double method of contraception from the Screening visit until completion of the study. The possibility of known or unknown drug interactions between the hormonal contraceptives and the investigational medicinal product always exist. Moreover, there is a possibility that the investigational product may lessen the effectiveness of a hormonal contraceptive agent. Hence female subjects with childbearing potential using hormonal contraceptives will not be included in the study.
Withdrawal Criteria	1. The patient withdraws the Informed Consent.<br>2. Patient's pregnancy: patients will be recommended to use effective contraception methods during the study, however if pregnancy occurs during the study, the IP/placebo treatment and other study procedures concerning the pregnant patient are cancelled, patient's pregnancy will be monitored up to pregnancy completion if the patient agrees.<br>3. Any medical condition of the patient that requires withdrawal of that patient from the study, as judged by the Investigator.<br>4. Patient lost to follow-up.
Restricted medication	The medications to be restricted during the study include drugs with nootropic, metabolic or sedative effects, or those which can exert such effects based on the Investigator's opinion or otherwise can strongly affect patient's performance upon CNS-VS testing. The Investigator should prescribe any of these drugs only upon consultation with the Sponsor and after the Sponsor approves the use of such medication.
Statistical methods	An overall comprehensive statistical analysis plan (SAP) for this clinical trial (including trial design) will be developed and finalized after trial initiation and before data lock/unblinding.<br>Data on demographics, laboratory results, vital signs, physical examination will be presented using the following descriptive statistics: number of observations, percentages, mean, standard deviation, median, quartiles, minimum and maximum values.<br>These baseline differences and differences in outcomes at 1, 3, 6 and 9-month follow-up will be summarized using means, standard deviations, medians, quartiles, minimums and maximums.<br>Baseline value for all parameters is defined as the most recent estimate before first drug intake.<br>Efficacy analysis

	Primary analysis will employ the Intention-to-treat population (ITT population), sensitivity analyses will be performed using ITT population with LOCF imputations and PP population.<br><br>Primary efficacy endpoint: Change in complex attention score, determined using Central Nervous System Vital Signs (CNS-VS) computer cognitive testing system, after 6 months of treatment compared to baseline in the group of patients receiving NeuroAiD II™ (MLC901), compared to the placebo group.<br><br>For primary endpoint analysis for each patient, the difference in complex attention scores, determined using CNS-VS computer testing, after 6 months of therapy compared to baseline, will be calculated: the change in complex attention score compared to baseline. Mixed effects model (PROC MIXED) will be used with adjustments for baseline and potential covariates. All timepoints will be used for the model. The participant and site will be used as the random effects. Model selection will be undertaken with each outcome using standard selection heuristics. Covariates will be selected based on improving the overall efficiency of the model. Regardless, baseline and age, gender, time since injury (1-3 months/4-12 months) and study center will be included as covariates in the mixed effects model. Descriptive statistics will be calculated to change in complex attention in each group, as well as mean values obtained by the method of least squares from the mixed effects model.<br><br>Superiority of MLC901 over placebo will be claimed if<br><br>H0: $LSM_T >= LSM_R$ is rejected and thereafter<br><br>H1: $LSM_T < LSM_R$ is accepted at significance level of 5%,<br><br>where LSM_T is a Least Square Mean of Change from Baseline of Complex attention score after 6 months of treatment assessed by the mixed model repeated measures analysis in the MLC901 group and LSM_R is a Least Square Mean of Change from Baseline of Complex attention score after 6 months of treatment assessed by the mixed model repeated measures analysis in the Placebo group (lower score of Complex attention is better).<br><br>Secondary efficacy endpoints and exploratory endpoints<br><br>The same approach as for primary endpoint will be used for analysis of secondary efficacy endpoints and exploratory endpoints, and completely similar hypotheses are be formulated as for the analysis of the primary endpoint. Mixed effects models will be used to analyze the change in values from baseline, adjusted for covariates and baseline estimates. No sensitivity analysis will be performed for secondary endpoints. No adjustments will be made for multiple comparisons, hypotheses will be tested hierarchically, hypothesis testing by secondary endpoints will be taken into account if statistically significant differences between groups for the primary endpoint are confirmed.<br><br>For all comparisons, a critical significance level of 0.05 was chosen.

	Safety analysis<br>Safety analysis will be performed in the safety population. Regardless of the reason for the completion of the study, the data of all patients who received at least one IP dose according to the assignments will be included in the safety analysis.<br>Adverse events will be coded using MedDRA dictionary. Incidences of TEAEs/SAEs will be calculated for assessment of AEs. Incidences of adverse events reported during the study will be presented as number of patients with AE in total and in each treatment group. Also, number of AEs per each severity category and per causal relationship with the study drug will be presented.<br>Incidences of adverse events will be compared between the IP and the Placebo group. The proportion of patients with at least one adverse event will be compared in the NeuroAiD II™ group and in the placebo group.
Sample size justification	Sample size was calculated using PASS 14 software.<br>Assumptions for sample size calculations:<br>An estimate of 20 for standard deviation of change in complex attention from baseline to 6 months was obtained from the BRAINS pilot study of NeuroAiD II (MLC901) in 78 patients with mild or moderate TBI. A minimally clinically meaningful difference of -10 in the mean change in complex attention, determined by CNS-VS, per group was determined. Target power - 80%. Two-sided significance level - 5%. The proportion of subjects in groups – 1:1. Hypotheses: H_0: $M_T \geq M_R$, H1: $M_T < M_R$, where M_T is an arithmetical mean of change of Complex attention score after 6 months of treatment, compared to baseline, in the MLC901 group, and M_R is an arithmetical mean of change from baseline of Complex attention score after 6 months of treatment in the placebo group (lower score of Complex attention is better).<br>128 subjects are required to detect a clinically meaningful difference of -10 in the mean changes in complex attention between MLC901 and placebo arms with 80% power at a two-sided 5% significance level. The sample size is increased to 182 subjects (91 per arm) to allow for 30% dropouts. Considering very high screening failure rate up to 364 subjects can be screened.
Version and Date	Version 2.0 dated April 8, 2021

1.3 Study Schedule

Table 1.1. Schedule of Study Procedures

Procedure	Screening (Visit 1)	Randomization (Visit 2)	Visit 3	Visit 4	Visit 5	Visit 6
**Time window, days**	**Day -14..-1**	**Day 1**	**Day 30±7**	**Day 90±14**	**Day 180±14**	**End-of-Study, Day 270 ±30**
Informed Consent Form	X[1]					
Inclusion/exclusion criteria assessment	X					
Medical History	X	X*				
Prior/Concomitant Medication Review	X	X*	X	X	X	X
Pregnancy test	X				X	
Demographic Data (age, race)	X					
Height, weight, BMI calculation[2]	X					
Collection of baseline characteristics (including social information)		X				
Date / time of TBI and injury characteristics including worst recorded GCS score	X					
Cognitive Failures Questionnaire (CFQ)	X					
Randomization		X[3]				
CNS-Vital Signs (Cognitive assessment)		X	X	X	X	X
Quality of life assessment (QOLIBRI)		X	X	X	X	X
Mood assessment (HADS)		X	X	X	X	X
Post-concussion symptoms (RPQ)		X	X	X	X	X
Physical Examination	X	X**	X	X	X	X
12-Lead ECG	X				X	
Vital Signs	X	X**	X	X	X	X
Clinical Laboratory Tests (Hematology, blood chemistry, urine)	X				X	
Compliance assessment			X	X	X	X
Adverse Event Monitoring		X	X	X	X	X
Investigational product supply		X	X	X		
Study drug administration		Day 1 – Day 180±14 days				

[1] Informed Consent Form must be obtained prior to performing any study-related procedures;
[2] Body weight and height will be obtained without outerwear and shoes; BMI = weight (kg)/height (m^2)

[3] Subjects will be randomized just prior to dosing. Further drug intake will be done according to the randomization scheme.
* Not necessary if complete information was obtained upon Screening
** Not necessary if screening (visit 1) and randomization (visit 2) are performed on the same day (which will be Day 1).

2 BACKGROUND INFORMATION

2.1 Rationale

Traumatic brain injury (TBI) is a leading cause of disability and death in young adults globally (1-3). It was estimated that about half of the population has suffered from a TBI at some moment in their life (around 90% of all TBI cases are mild TBI), there are about 50 million people experiencing new TBI annually and the burden of TBI is increasing globally (1,4). The annual global cost of TBI is already immense and estimated to be around 400 billion US dollars (1).

Injury to the brain is caused from the mechanical impact of the brain onto the bony surfaces within the skull or from penetration of objects into the skull; and diffuse axonal injury (DAI) as a result of rotational forces as the brain moves within the skull. As a result of injury brain cells can die, affecting the functioning of areas that they help to control e.g. causing neurological/cognitive deficits. The most frequent sites of cerebral contusion in closed TBI are the temporal and basal-frontal regions, both of which are associated with cognitive functioning (5,6).

Persistent cognitive deficits have been reported to occur in up to half of adults following a mild TBI and can profoundly impact a person's day-to-day functioning, often affecting their ability to return to work, or impacting their capacity to engage in independent living (6,7). The most common cognitive deficits include difficulties with complex attention, executive functioning, cognitive flexibility and memory. After a brain injury (particularly for mild injuries) many people recover spontaneously as the brain mobilises surviving elements of the central nervous system in the damaged area to facilitate recovery (8). However often this spontaneous recovery process is insufficient, and people continue to experience cognitive, emotional and physical impairments (9-11).

Despite the huge and increasing burden from mild TBI, there is still no proven effective pharmacological treatment to improve post-TBI cognitive functioning and further research into potential new interventions is needed. Due to the complexity of the recovery processes involved after injury to the brain, there is increasing evidence that the search for a single molecule which specifically acts on a single target is not optimal. Combination therapies comprising more than one active ingredient may offer a better treatment strategy. Herbal medicine may represent a valuable resource in such a search for safe and effective therapy.

NeuroAiD II™ (MLC901) is a traditional Chinese medicine, a simplified formulation of MLC601 (NeuroAiD I). MLC601 (NeuroAiD I) contains 9 herbal and 5 non-herbal components and has extensive historical use in humans, NeuroAiD II™ (MLC901) contains only the 9 herbal components. The herbal components of MLC601 and MLC901 are the same.

NeuroAiD II™ has been shown to both protect brain cells from dying after injury in addition to stimulating generation of new neural cells, connections and pathways (reviewed in detail in the IB (36)). NeuroAiD II™ treatment has been shown to be followed by cognitive improvement in patients who had experienced some cognitive impairment after mild or moderate TBI (21). Taking into account the good safety profile of NeuroAiD II™, it is supposed to provide benefit for patients who had experienced mild or moderate TBI, manifested in improvement in TBI-associated cognitive impairment.

2.2 Name and Description of the Investigational Product

Table 2.1. Name and Description of the Investigational Product

**NeuroAiD II™ (MLC901)**	
**Trade name:**	NeuroAiD II™ (MLC901)
**International Non-proprietary Name (INN):**	NeuroAiD II™ (MLC901)
**Manufacturer/Marketing Authorization Holder:**	Moleac Pte Ltd,<br>Helios #09-08, 11 Biopolis Way, Singapore 138667
**Dosage form:**	Capsule 400 mg (size 0)
**Dosage:**	Recommended treatment is 2 capsules orally, 3 times a day (i.e. 6 capsules per day). Standard treatment is 12 weeks. MLC901 capsules could be:<br>- swallowed as such with water<br>- opened and drunk once powder has been diluted in a sufficient amount of water of room temperature *<br>**Clumping of the powder content may be observed due to the inherent nature of the powder extract. This does not indicate deterioration of quality if used within the specified shelf life.*
**Appearance:**	The capsule cap is dark blue and capsule body is light blue in color. The capsule size is 0. The powder content is brown to dark brown color and has some agglomerates.
**Storage terms:**	To be stored in a cool dry place not exceeding 30°C
**Package and labelling:**	Capsules are gelatin-free. They are made up of Hypromellose (hyrodypropyl methycelluose HPMC) which is permitted as a food additive for human consumption in accordance with 21 CFR 172.874. The capsules are packed in blister made of PVDC/PE/PVC multilayer clear blister front and 25-micron aluminum backing with 12 capsules per stip. Every 5-blister strip (i.e. 60 capsules) is prepacked into an aluminum sachet before being placed in a box.

Each IP kit contains 3 sachets. One sachet contains 5 blisters, and one blister contains 12 capsules. One capsule contains 400 mg of NeuroAiD II™ (MLC901) or Placebo.

The herbal extract powder of NeuroAiD II™ (MLC901) capsules is inherently hygroscopic in nature, and it easily absorbs moisture which cause agglomeration. Therefore, the packaging of MLC901 capsules (aluminum sachet with desiccant as additional protective layer to pack the blisters) has been designed to reduce formation of agglomerates and provide the necessary protection in this regard. Hence, it is not allowed to open all sachets (for 2 months' supply) to label

the blisters with the visit and randomization numbers during dispensing. In view of this, the visit and randomization numbers on the blister labels couldn't be indicated to ensure that the quality of NeuroAiD II (MCL 901) capsules is maintained throughout the treatment duration. Also, patients should be informed to keep blisters in the sachet after opening the sachet (i.e. to put the blister into the sachet after each IP administration).

2.2.1 Description and Composition of the Drug Product

The MLC901 drug product comprises the extracts of 9 botanical active ingredients blended with commonly used excipients. The 9 botanical active ingredients are traditional herbs well documented in the Pharmacopoeia of the People's Republic of China. It is intended for oral administration and presented in the form of hard hypromellose (HPMC) capsules (size 0) with a content weight of 400 mg.

Each 400-mg capsule contains extracts of botanical active ingredients added with excipients in the quantities specified in Table 2.1.

Table 2.2. Composition of MLC901.

**Active Ingredients**				
	**Ingredients (Latin names)**	**Parts Used**	**Quantity* (mg) per capsule**	**Equivalent extract per capsule (mg)**
1	*Radix Astragali*	Dried root	800	111.5
2	*Radix et Rhizoma Salviae miltiorrhizae*	Dried root/rhizome	160	22.3
3	*Radix Paeoniae rubra*	Dried root	160	22.3
4	*Rhizoma chuanxiong*	Dried rhizome	160	22.3
5	*Radix Angelicae sinensis*	Dried root	160	22.3
6	*Flos Carthami*	Dried flower	160	22.3
7	*Semen persica*	Dried ripe seed	160	22.3
8	*Radix polygalae*	Dried root	160	22.3
9	*Rhizoma Acori tatarinowii*	Dried rhizome	160	22.3
**Excipients**				
	**Name**	**Functions**	**Quantity (mg) per capsule**	

1	Dextrin	Bulking agent	57
2	Maltodextrin	Bulking agent	51
3	Magnesium stearate	Lubricant	2
**Ingredients of Capsule Shell**			
	**Name**	**Functions**	**Quantity (mg) per capsule**
1	Brilliant blue FCF-FD&C Blue 1	Capsule colorant	0.0819
2	Titanium dioxide	Capsule-opacifier	1.895
3	Hypromellose	Capsule- structure	89.79
4	Water	Solvent	4.23

* Based on input quantity of raw herbs used for extraction.

There are no ingredients from animal origin in MLC901 (only herbal extracts).

NeuroAiD is a traditional medicine that was certified as being compliant with good manufacturing practice and was registered under the Chinese name Danqi Piantan Jiaonang with the Sino-Food and Drug Administration (Sino-FDA) in August 2001 for the treatment of stroke (MLC601) and included both herbal and non-herbal components (12). The formula of NeuroAiD has subsequently been simplified (NeuroAiD II™, MLC901) to include only the 9 herbal components (*Radix astragali, Radix salvia miltiorrhizae, Radix paeoniae rubra, Rhizoma chuanxiong, Radix angelicae sinesis, Carthamus tinctorius, Prunus persica, Radix polygalae* and *Rhizoma acori tatarinowii*), yet showing similar efficacy (13).

NeuroAiD II™ (MLC901) has several advantages over the parent product that may lead to improved patient compliance. The MLC901 formulation excluded animal ingredients that are present in NeuroAiD (MLC601), allowing the need for consumption of less capsules per day with no increase in the size. The capsule shell is made of hypromellose instead of gelatin, hence is suitable for vegetarians. MLC901 should inherently be just as safe as MLC601. It was registered in Singapore as Chinese Proprietary Medicine in March 2010 and has been commercialized in more than 30 countries. MLC901 has the potential to facilitate the restoration of neuronal circuits through its antioxidant properties and promotion of cell proliferation and stimulation of axonal and dendritic neuronal circuits (13,14).

2.3 Summary of Findings from Nonclinical Studies that Potentially have Clinical Significance and from Clinical Studies that are Relevant to the Study

2.3.1 Nonclinical studies

MLC601 and MLC901 have been shown to demonstrate neuroprotective, anti-inflammatory, and neurorestorative properties in animal and cellular models of cerebral ischemia and other brain injuries. NeuroAiD II™ has been shown to both protect cells from dying after injury in addition to stimulating the generation of new neural cells, connections and pathways (13, 14; reviewed in detail in the IB (36)).

This may be due to stimulation of the growth factor BDNF which regulates neuronal survival (13) and increased cerebral flow velocity (15). Previous studies on NeuroAiD II™ have focused on the use in rats in specific animal models, and both healthy controls and people who have experienced a stroke (an injury to the brain from internal causes). In rats two studies have revealed that animals given NeuroAiD II™ after an ischemic injury show improved survival, neurological recovery and decreased neuro-degeneration in the acute phase following injury (13,14,36).

Along with enhanced neuronal survival and recovery, cognitive and neurological improvements occurred with MLC601 and MLC901 following traumatic brain injury (TBI) as shown by Tsai et al. and Quintard et al. (37,38).

A series of motor tasks, i.e. spontaneous forelimb elevation, foot-fault placing test, and ladder-climbing test, were used by Tsai et al (38) to assess motor functions. MLC601 treatment after TBI in rats significantly improved neurological and motor outcomes which were correlated with attenuation in contusion volume, fewer apoptotic neurons and less microgliosis. The rats with TBI, administered intraperitoneally with 0.4 mg/kg MLC601, performed statistically significantly better in all the above tests than did vehicle-treated rats with TBI between 4 and 21 days after TBI. MLC601 was administered 4 days after TBI and then once a day for 2 consecutive days.

In the study conducted by Quintard et al. (37), a new approach to behavior was used with a modified version of the Novel Object Recognition Task which allows investigation of the ''what-where-when'' dimensions of episodic memory in rodents. TBI was induced by a moderate lateral fluid percussion applied to the right parietal cortex. MLC901 was injected intraperitoneally (0.075 mg/ml in a bolus of 500 µl) at 2 h post-TBI, and then administered in drinking water at a concentration of 10 mg/ml until sacrifice of the animals. MLC901 treatment decreased brain lesions induced by TBI. It prevented the serum increase of S-100 beta (S100B) and neuron-specific enolase (NSE), reduced the infarct volume when injected up to 2 h post-TBI, prevented edema formation and assisted its resolution, probably via the regulation of aquaporin 4. These positive MLC901 effects were associated with an upregulation of vascular endothelial growth factor (VEGF) as well as an increase of endogenous hippocampal neurogenesis and gliogenesis around the lesion.

Quintard et al. clearly showed that TBI reduced object exploration and totally suppressed temporal order memory but not spatial memory. In the episodic-like memory test, the rats after TBI which received vehicle, failed to discriminate between the two 'old' objects and the two 'recent' ones.

MLC901 completely reversed this severe impairment in TBI rats and restored the performance of the MLC901-treated TBI rats to that observed in sham rats. Analysis of temporal order memory 7 days after TBI showed that the MLC901-TBI rats' performances were above chance level and similar to those of MLC901-Sham rats. These results show that MLC901 makes possible the recovery of the temporal order memory, which is impaired after TBI.

This data is consistent with the hippocampal neurogenesis and elevated BDNF expression observed with MLC901 (13).

2.3.2 Clinical studies

Efficacy and safety of NeuroAiD in both healthy controls and stroke patients were reported by Gan et al. (20), both the immediate and long-term effects of NeuroAiD were tested. In the article three studies are reported which revealed that there were no statistically or clinically significant differences in haemostatic, haematological or biochemical parameters due to NeuroAiD. These safety findings were supported in a subsequent study by Young et al (17) in 114 stroke patients

where they revealed that there were no statistically or clinically significant differences in biochemical and haematological parameters or on electrocardiogram tests between participants taking the NeuroAiD and taken placebo at 3 months. On the other hand, there is evidence suggesting a beneficial effect of NeuroAiD on cerebral blood circulation and functional outcomes post-stroke. To explore the possible impact of NeuroAiD on cerebral blood flow, a study was conducted with 80 patients post-stroke (15). It was revealed that participants taking NeuroAiD demonstrated increased cerebral blow flow velocity in comparison to placebo. It was also observed that participants taking NeuroAiD had higher scores of a measure of everyday functioning (Barthel Index).

Other studies have explored the influence of NeuroAiD on functional outcomes, including a small clinical trial conducted with 40 patients one month following stroke (18). Trends in improvement of limb functioning were observed in the NeuroAiD group in comparison to placebo. The frequency of reporting of adverse events was equal between groups and all events were classified as mild or moderate in severity. Subsequently a study was completed in Iran with 150 patients up to 1 month post-stroke which revealed that participants taking NeuroAiD for 3 months showed improved scores on motor functioning in comparison to those taking placebo capsules (16). A larger study of NeuroAiD in a pooled sample of 605 patients post-stroke also demonstrated trends in improvement in physical functioning in participants taking NeuroAiD (12). However this study has some methodological limitations that impact on the interpretation of the results e.g. the control group took an active alternative Chinese medicine (Buchang Naoxintang Jiaonang).

Two studies of NeuroAiD II™ (MLC901) in patients after acute ischemic stroke have been reported (36) with treatment duration of 1 and 3 months (Protocols #EFSA2011_01, EFSA2014_04, 36,49,50). After one month of treatment, MLC901 in addition to standard treatment and early rehabilitation after a stroke was associated with better improvements in neurological, cognitive, functional, emotional, and disability parameters compared to standard treatment and rehabilitation alone (36,49):

- NIHSS (National Institutes of Health Stroke Scale; 0 - No stroke symptoms, 1–4 Minor stroke, 5–15 Moderate stroke, 16–20 Moderate to severe stroke, 21–42 Severe stroke),
- MMSE (Mini-Mental Status Examination; questionnaire to measure cognitive impairment),
- BI (Barthel Index; ordinal scale of performance in activities of daily living),
- Rehabilitation Activities Profile (assessment of communication, mobility, personal care, occupation, and relationships, based on International Classification of Impairments, Disabilities and Handicaps)
- HADS (Hospital Anxiety and Depression Scale)

In an observational study, after a 3-month course of MLC901 as add-on treatment to standard rehabilitation therapy, an association between the use of MLC901 and recovery was detected. Multivariate analyses identified baseline Barthel Index score, time to treatment, and MLC901 to be positive predictors of improvement. Among patients with more severe stroke (NIHSS >14), more patients who received MLC901 showed above-median improvements on mRS (modified Rankin Scale; measures the degree of disability or dependence in the daily activities of people after stroke) compared to those who did not, at month 1 (71.4% vs. 29.4%; $p = 0.032$), and a similar trend was retained at month 3 (85.7% vs. 50%; $p = 0.058$).

A pilot study by Theadom et al. (21) of NeuroAiD II™ (MLC901) in N=78 adults with impaired CFQ at baseline revealed significant improvements [as measured by Cohen's d effect size] in

complex attention (P=0.04) and executive functioning (P=0.04) following mild to moderate TBI following 6 months of treatment compared to controls. Additionally, there was a small improvement in the cognitive domain of quality of fatigue. As few participants experienced physical functioning deficits following TBI, a ceiling effect on the GOS-E measure was noted. There were no trends in effect noted for fatigue. It was concluded that a full scale, multi-centre clinical trial is needed to determine clinical efficacy of NeuroAiD II™ (MLC901) in adults following TBI on cognitive functioning, symptoms and quality of life.

Another study by Al Fauzi et al. (22), called Neurological Prognosis after brain Trauma and Use of NeuroAiD™ (NEPTUNE) in a population of N=32 adults, aimed to assess the effects of MLC601 on the functional and neurological outcome of patients with non-surgical moderate TBI (GCS 9 to 13) when given within 2 days of injury. All patients were followed for 6 months. On Barthel Index, the MLC601 group had higher median values compared to the control group at all time points, reaching significance at month 3 (47.5 vs. 35.0; p = 0.014) and at month 6 (67.5 vs. 57.5; p = 0.055). Trajectories of BI over time showed significant improvement of Barthel Index in the MLC601 group from time of discharge to month 3 to month 6 (40.0 to 47.5 to 67.5, respectively).

Safety pharmacology of NeuroAiD (MLC601) and NeuroAiD II (MLC901), both from clinical studies and spontaneous reports from markets, is detailed in the Investigator's Brochure, Section 5.3. Clinical Safety. By now, no data suggesting negative influence of NeuroAiD (MLC601) and NeuroAiD II (MLC901) on the cardiovascular, respiratory and central nervous system or specific types of toxicity has been reported. Data from clinical studies confirm that NeuroAiD does not affect blood hematology, blood biochemistry and hemostasis, ECG parameters, renal and liver functions (12,16,17,20,36).

In the reported clinical studies of NeuroAiD I or II occurrence of adverse events in patients receiving NeuroAiD was not higher than AE occurrence in the control group. Among adverse events, the more commonly reported with treatment were predominantly gastrointestinal, e.g., nausea, vomiting, diarrhea, and abdominal discomfort, which may be attributable to the number of capsules taken per day, i.e., 4 capsules three times a day for MLC601 and 2 capsules three times a day for MLC901. Allergic reactions and gastrointestinal tract reactions are expectable due to the herbal nature of the drug. However, according to clinical data, they were rather rare and mild.

Due to severity of underlying neurological diseases (including stroke, dementia) serious adverse events were recorded in the studies of NeuroAiD. There was no overall increase in serious adverse events reported, they were mostly related to complications of the underlying disease.

Two clinical studies demonstrated clinical safety of MLC601 in healthy subjects and stroke patients: MLC601 did not modify hemostatic, hematological, and biochemical parameters in normal subjects (43 patients) and stroke patients (10 patients) (20). Patients after ischemic stroke received MLC601 in addition to aspirin and other standard medications for associated medical conditions, such as diabetes mellitus, hypertension, hypercholesterolemia, and ischemic heart disease. No significant differences were observed in hemostatic, hematological, and biochemical parameters tested at 1 and 4 weeks compared to baseline.

In a safety study (80 patients with ischemic stroke received MLC601) there was no statistical difference in hemoglobin, creatinine, sodium, potassium, ALT and AST at the different specified timepoints up to 6 months (39).

Reports of open-label use in stroke patients observed low rates of adverse events, with only 4 cases (of which only 2 were considered likely related to treatment: diarrhea and "heatiness") among 24 patients, and only 3 "side effects" reported as reason for discontinuing treatment among 192 patients (19,40).

Earlier completed randomized controlled studies reported very low rates of adverse events in the study treatment group which were not different from the active control or placebo group (12,15,16,18,41,39).

The randomized, double-blind, placebo-controlled CHIMES Study randomized 1099 patients from Singapore, Philippines, Thailand, Malaysia, Hong Kong and Sri Lanka who suffered an ischemic stroke of intermediate severity within 72 hours to either MLC601 4 capsules 3 times a day or matching placebo for 3 months (42,43). The first 114 subjects were included in a clinical and laboratory safety study (17). Occurrences of adverse events (AEs) were similar between the treatment groups and were commonly related to the underlying stroke. There were no statistically significant differences between treatment groups in all the hematology tests at 3 months.

At the end of the CHIMES Study, 542 patients on MLC601 and 545 on placebo were included in the safety analysis. 230 (42%) subjects on MLC601 reported 459 AEs compared to 218 (40%) subjects on placebo reporting 504 AEs. The frequency of MedDRA preferred terms by system organ class is provided in IB (36, Section 7 Appendix).

The pre-planned CHIMES-E extension study (EFSA2007_04) followed up CHIMES patients (n=880) up to 2 years from study inclusion (44,45). While no further treatment was given apart from standard stroke therapies and rehabilitation, it provided further opportunity to assess the long-term efficacy and safety of MLC601 even after the study treatment was already discontinued. The study showed that by month 24, rates of death and occurrence of any vascular event and other medical events classified according to organ system were similar between treatment groups. In particular, there was no difference in the rates of renal or hepatic adverse events. Neoplasm was reported in 4 subjects each for the MLC601-treated (gynecologic 1, lung 2, urinary 1) and placebo-treated (parathyroid 1, lung 2, urinary 1) groups.

Table 2.3. Number of patients experiencing medical events over 24 months in the CHIMES-E Study (44,45)

Medical Events	MLC601 (n=446)	Placebo (n=434)
Death	28 (6.3%)	29 (6.7%)
Vascular event*	56 (12.6%)	55 (12.7%)
Central nervous system, non-vascular	24 (5.4%)	19 (4.4%)
Cardiac, non-vascular	5 (1.1%)	3 (0.7%)
Hepatobiliary	2 (0.4%)	2 (0.5%)
Renal	10 (2.2%)	9 (2.1%)
Hematologic	12 (2.7%)	8 (1.8%)
Dermatologic	2 (0.4%)	0 (0.0%)
Endocrine	9 (2.0%)	5 (1.2%)
Gastrointestinal	18 (4.0%)	16 (3.7%)
Gynecologic	2 (0.4%)	0 (0.0%)
Infection	23 (5.2%)	26 (6.0%)
Ophthalmologic	0 (0.0%)	1 (0.2%)
Orthopedic	7 (1.6%)	7 (1.6%)
Psychiatric	4 (0.9%)	1 (0.2%)
Pulmonary	3 (0.7%)	6 (1.4%)

Medical Events	MLC601 (n=446)	Placebo (n=434)
Rheumatologic	6 (1.3%)	7 (1.6%)
Urinary	5 (1.1%)	6 (1.4%)

* Recurrent stroke or transient ischemic attack, acute coronary event, pulmonary embolism, peripheral vascular disease, sudden death

In a recent clinical study of NeuroAiD II™ (MLC901) in patients with mild TBI, NeuroAiD II™ (MLC901) was found to be safe and well tolerated in TBI patients (21). In the 36 patients with mild TBI who received NeuroAiD II™ (MLC901), 3 adverse events were observed: headache in 1 patient, sore tongue in 1 patient, itchiness in 1 patient. Hematuria due to a secondary medical condition was observed in one patient. In the Placebo group, consisting of 42 patients, adverse events included difficulty sleeping, headache, itchiness and upset stomach. These adverse events were minor. No serious adverse events were reported.

Moleac's pharmacovigilance database reveals that only minor side effects have been reported including: nausea, vomiting, diarrhoea, dizziness, headaches, dry mouth, digestive discomfort (due to the high herbal concentration), and various allergic reactions with similar findings in the published clinical trials reviewed (16-19). Of all serious adverse events observed in the clinical trials post-stroke "all events were commonly observed post-stroke including stroke progression, recurrent stroke and cardiac events" (17).

Details of clinical and preclinical studies of IP are provided in the IB (36).

2.4 Summary of the Known and Potential Risks and Benefits, if any, to Human Subjects

Risks to subjects in this study are related to:

- common procedures performed
- documented adverse events listed in the current reference products information.

These risks are communicated to the subjects in the consent forms.

Based on the safety profile of NeuroAiD II™, the risks through participating in the trial are considered low. Clinical safety data from MLC601 and MLC901 showed no overall increase in adverse events, most commonly reported AEs upon treatment were predominantly gastrointestinal, e.g. nausea, vomiting, diarrhea, and abdominal discomfort, which may be attributable to the number of capsules taken per day. The detailed list of expected events, that have been observed after IP administration in previous studies, is given in the respective section of IB (36).

Risks for subjects related to study procedures required by this protocol are minimal. The risk of participating in this clinical trial, similar to other trials, is related to blood drawing via venipuncture. Although hardly probable but there exists a possible risk of infection during blood sampling procedure. This risk can be eliminated by professionally performed procedure with qualified professional medical staff.

The total blood draw volume in this study is no more than 20 ml, which will be taken twice (in the beginning of the study and upon completion). Normal blood donation volume is up to 500 mL for one procedure, therefore the blood volume drawn in this study is incomparably lower than upon blood donation and there is no risk for volunteers in comparison to blood donation procedure.

The patients recruited in both NeuroAiD II™ and placebo are not in severe condition and are allowed to receive any other mTBI treatment besides IP. On the other hand, patients, receiving

placebo, avoid the risks, associated with IP intake and possible AEs, produced by its active substances.

Current study incorporates several measures to mitigate risks:

- measures will be taken during screening to ensure safety of the study subjects;
- the protocol specifies medical surveillance over the patients which includes recording and evaluation of AEs;
- in case of a known adverse reaction to the drug product, the investigator will take all the necessary actions, including discontinuation of the drug product;
- qualified personnel will perform the procedures in the scope of this study.

The benefit for the patient from participation in this clinical trial includes:

- thorough multiple-scale diagnostics of patient's condition after TBI;
- surveillance of dynamics of patient's condition for 9 months – during the study and follow-up period.

Risk-Benefit Assessment

Based on the information presented, benefits from participation in this study outweigh risks for patients in both treatment arms. Therefore, the benefit: risk balance supports the investigation of NeuroAiD II™ (MLC901) in the defined study population.

2.5 Description of and Justification for the Route of Administration, Dosage, Dosage Regimen, and Treatment Period

The IP/placebo will be administered orally in the dose of 2x400-mg capsules 3 times a day for 6 months. The route of administration, dosage, dosage regimen, and treatment period in this study are those used in the pilot study of efficacy and safety of NeuroAiD II™ (MLC901) in patients with mild and moderate TBI with the same design (21).

The pilot study by Theadom et al. (21) included 78 patients with moderate or mild TBI, of which only 2 patients (3%) had confirmed moderate TBIs, the vast majority of cases were mild TBIs. The dosing and regimen used were standard therapeutic doses, safety and efficacy of these doses and dosing regimen have been previously confirmed in clinical studies (21,36). The pilot study confirmed high tolerability and safety of NeuroAiD II™ (MLC901), taken in the abovementioned doses, together with significant improvement of patient's cognitive functioning, evaluated using the CNS-VS system (21).

Safety of MLC 901 in human is supported by the following:

- MLC901 contains extracts of nine herbal components which have been used individually as dietary supplement ingredient and as traditional medicine.
- The proposed raw daily dose (prior to extraction) of each herb in MLC901 is within the recommended dosage in the Chinese Pharmacopeia.
- MLC 601 and MLC901 have shown safety in humans in clinical trials and adverse event reporting.

NeuroAiD II™ (MLC901) is a traditional Chinese medicine, a simplified formulation of MLC601 (NeuroAiD I). MLC601 (NeuroAiD I) contains 9 herbal and 5 non-herbal components and has extensive historical use in humans, NeuroAiD II™ (MLC901) contains only the 9 herbal

components. The herbal components of MLC601 and MLC901 are the same. MLC601 is the precursor formulations of MLC901. The raw daily doses of the nine herbal components in MLC901 are comparable to those in MLC601.

The dose of MLC601 used in all the clinical studies in stroke, TBI and other neurological conditions was 4 capsules 3 times a day, each capsule being 400 mg with total daily dose is 4800 mg/day. The dose of MLC901 in completed and currently ongoing studies for other indications, e.g., vascular cognitive impairment, traumatic brain injury, and spinal cord injury, is 2 capsules 3 times a day, each capsule being 400 mg, with a total daily dose of 2400 mg/day. The details/publications of studies conducted with MLC901 and its precursor MLC601 are summarized in Appendices of the Investigator Brochure (36).

Clinical safety data from MLC601 and MLC901 showed no overall increase in adverse events, although more commonly reported with treatment are predominantly gastrointestinal, e.g., nausea, vomiting, diarrhea, and abdominal discomfort, which may be attributable to the number of capsules taken per day, i.e., 4 capsules three times a day for MLC601 and 2 capsules three times a day for MLC901. There was no overall increase in serious adverse events reported which were mostly related to or complications of the underlying disease. The therapeutic dose of MLC901 is 2 capsules 3 times a day. With only herbal ingredients, MLC901 is expected to have a comparable or even better safety profile than its precursor.

Overall Extent of Exposure in the clinical studies to justify dose rationale of MLC901

The oral dose investigated in most studies in stroke was 4 capsules 3 times a day (4800 mg/day) for MLC601 and 2 capsules 3 times a day (2400 mg/day) for MLC901, often given for a duration of one to three months. In four early safety and efficacy studies of MLC601 in neurodegenerative cognitive conditions (Alzheimer's disease, mild cognitive impairment and vascular dementia) MLC601 was given at dose of 1 capsule 3 times a day (1200 mg/day). Treatment duration was 16 and 18 months in the two Alzheimer's disease studies (Protocols #EFSA2011_03 and EFSA2013_01), and 6 months in the study of mild cognitive impairment (EFSA2015_01).

Details of study designs and patients' exposures to MLC601 and MLC901 in clinical studies are summarized in the investigator brochure. Data on study participants allocated to MLC601 or MLC901 in controlled and uncontrolled clinical studies by dose and treatment duration is listed in Table 2.4.

Table 2.4 Study participants allocated to MLC601 or MLC901 in controlled and uncontrolled clinical studies by dose and treatment duration

**Treatment Duration**	**Dosage**				**Total**
	**4 capsules for 2 doses (3200 mg)**	**1 capsule 3 times a day (1200 mg/day)**	**2 capsules 3 times a day (2400 mg/day)**	**4 capsules 3 times a day (4800 mg/day)**	
**Formulation: MLC601 (400 mg capsules)**					
≤ 1 day	SAFE2004_01: 54	-	-	-	54
1 month	-	-	-	SAFE2004_01: 10<br>EFSA1999_01a: 100<br>EFSA1999_01b: 300<br>EFSA2008_01: 20	430

**Treatment Duration**	**Dosage**				**Total**
	**4 capsules for 2 doses (3200 mg)**	**1 capsule 3 times a day (1200 mg/day)**	**2 capsules 3 times a day (2400 mg/day)**	**4 capsules 3 times a day (4800 mg/day)**	
2 to 3 months	-	-	-	EFSA2007_01a: 10	10
3 months	-	-	-	EFSA2009_01: 21<br>EFSA2009_02: 40<br>EFSA2009_03: 100<br>EFSA2007_03 / EFSA2007_04: 550<br>EFSA2011_02: 30<br>SAFE2010_01: 95<br>EFSA2007_02: 217	1053
1 to 6 months	-	-	-	EFSA2007_01b: 24	24
6 months	-	EFSA2015_01: 36	-	-	36
16 months	-	EFSA2013_01: 66	-	-	66
18 months	-	EFSA2011_03: 124	-	-	124
24 months	-	EFSA2014_01: 41	-	-	41
Subtotal	54	267	-	1517	**1838**
**Formulation: MLC901 (400 mg capsules)**					
1 month	-	-	EFSA2011_01: 15	-	15
3 months	-	-	#EFSA2014_04: 131	-	131
6 months	-	-	EFSA2014_02: 36	-	36
Subtotal	-	-	15	-	**182**

2.6 Regulatory Requirements to be Considered During Study Conduction

The study will be carried out according to the protocol and in compliance with Principles of the World Medical Association`s Declaration of Helsinki (Fortaleza, Brazil, 2013), Tripartite Agreement on the Good Clinical Practice (ICH GCP), rules for Good Clinical Practice of the Eurasian Economic Union, and the laws and applicable regulatory requirements of Russian Federation, including:

- Federal Law of the Russian Federation of November 21, 2011 No. 323- FZ "On fundamental healthcare principals in the Russian Federation" as presently in effect;
- Federal Law No. 61- FZ "On medicinal products circulation", current version;
- Order of the Ministry of Health of the Russian Federation № 200n dated April 1, 2016 “On approval of the rules of Good Clinical Practice”;
- Regulations on compulsory life and health insurance of the patient participating in the clinical studies of the drug approved by the Government Decree of the Russian Federation of September 13, 2010 No. 714 as presently in effect;
- Federal Law of July 27, 2006 No. 152- FZ "On personal data" as presently in effect;

- Eurasian Economic Commission Council Resolution dated November 3, 2016, No. 79 “Concerning approval of Guideline on good clinical practices”
- Eurasian Economic Commission Council Resolution dated November 3, 2016, No. 78 “Concerning Rules for Registration and Expertise of Medicines for Medical Use”
- The Order of Federal Service for Supervision in Healthcare (Roszdravnadzor) No. 1071 dated 15.02.2017 “On approval of the Pharmacovigilance Procedure”.

2.7 Description of the Population to be Studied

Adult patients who experienced mild TBI 1-12 month prior to the study and have cognitive impairment following injury (determined by the Cognitive Failures Questionnaire score >30), will be included in accordance with Inclusion/Exclusion criteria.

2.8 Critical processes and data identification

Critical information in the Study will include:

- data of CFQ assessment upon screening since patient’s condition after quite a long period after mild TBI is not evident
- data of all assessments using all the CNS-VS modules (complex attention, executive functioning, processing speed, memory (visual and verbal) and reaction time)
- data of mood assessment with HADS
- data of quality of life assessment using QOLIBRI
- data of post-concussion symptoms assessment using RPQ
- safety data (incidences of TEAEs, SAEs, data on deviations in results of laboratory tests)

2.9 References to Literature and Data that are Relevant to the Study, and that Provide Background for the Study

See Section 16.

3 STUDY AIM, OBJECTIVES AND ENDPOINTS

3.1 Study aim

To determine the efficacy of NeuroAiD II™ (MLC901) in improvement of cognitive functioning of adult patients with long-term cognitive impairment following mild traumatic brain injury (mTBI) and to assess safety of NeuroAiD™ (MLC901) in these patients.

3.2 Objectives and endpoints

3.2.1 Efficacy

- To determine the effect of NeuroAiD II™ (MLC901) on changes in:
  - complex attention,
  - other cognitive domains: executive functioning, processing speed, memory (visual and verbal), reaction time
  - post-concussion symptoms
  - quality of life
  - levels of anxiety and depression

in adult patients with long-term cognitive impairment following mTBI compared to baseline after 6 months of NeuroAiD II™ (MLC901) treatment with reference to placebo-treated adult patients with long-term cognitive impairment following mTBI.

- In case of any improvement in any of the above parameters upon completion of NeuroAiD II™ (MLC901) treatment: to assess changes in improved parameters 3 months after treatment completion with reference to the placebo-treated group.

Study efficacy objectives and the corresponding primary, secondary and exploratory endpoints are detailed in Table 3.1.

Table 3.1. Study efficacy objectives and endpoints.

Objectives	Endpoints
**Primary**	
To determine the effect of NeuroAiD II™ (MLC901) on changes in complex attention in adult patients with long-term cognitive impairment following mTBI compared to baseline after 6 months of NeuroAiD II™ (MLC901) treatment with reference to placebo-treated adult patients with long-term cognitive impairment following mTBI	Change in complex attention score, determined using Central Nervous System Vital Signs (CNS-VS) computer cognitive testing system, after 6 months of treatment compared to baseline in the group of patients receiving NeuroAiD II™ (MLC901), compared to the placebo group.
**Secondary**	
To determine the effect of NeuroAiD II™ (MLC901) on changes in cognitive functioning across other cognitive domains: executive functioning, processing speed, memory (visual and verbal) and reaction time in adult patients with long-term cognitive impairment following	Changes in the scores for the following cognitive domains: executive functioning, processing speed, memory (visual and verbal) and reaction time, determined by CNS-VS system, after 6 months of treatment compared to baseline, in the NeuroAiD II™ (MLC901) group, compared to the placebo group.

mTBI compared to baseline after 6 months of NeuroAiD II™ (MLC901) treatment with reference to placebo-treated adult patients with long-term cognitive impairment following mTBI.	
To determine the effect of NeuroAiD II™ (MLC901) on changes in post-concussion symptoms in adult patients with long-term cognitive impairment following mTBI compared to baseline after 6 months of NeuroAiD II™ (MLC901) treatment with reference to placebo-treated adult patients with long-term cognitive impairment following mTBI.	Change in the Rivermead Post Concussion Symptoms Questionnaire (RPQ) total score, after 6 months of treatment, compared to baseline, in the NeuroAiD II™ (MLC901) group compared to the placebo group.
**Exploratory Endpoints**	
To determine the effect of NeuroAiD II™ (MLC901) on changes in quality of life in adult patients with long-term cognitive impairment following mTBI compared to baseline after 6 months of NeuroAiD II™ (MLC901) treatment with reference to placebo-treated adult patients with long-term cognitive impairment following mTBI.	Change in the Health-related quality of life questionnaire (QOLIBRI) total score after 6 months of treatment, compared to baseline, the NeuroAiD II™ (MLC901) group compared to the placebo group.
To determine the effect of NeuroAiD II™ (MLC901) on changes in levels of anxiety and depression in adult patients with long-term cognitive impairment following mTBI compared to baseline after 6 months of NeuroAiD II™ (MLC901) treatment with reference to placebo-treated adult patients with long-term cognitive impairment following mTBI.	Change in the Hospital Anxiety and Depression Scale (HADS) score after 6 months of treatment, compared to baseline, in the NeuroAiD II™ (MLC901) group compared to the placebo group.
In case of any improvement in any of the above parameters after 6 months of NeuroAiD II™ (MLC901) treatment: to assess changes in improved parameters in the same adult patients with long-term cognitive impairment following mTBI after 9 months of treatment initiation (i.e. 3 months after treatment completion) with reference to the same placebo-treated group of adult patients with long-term cognitive impairment following mTBI.	Changes, compared to baseline, in the analyzed parameters (complex attention, executive functioning, processing speed, visual and verbal memory, reaction time, RPQ score, QOLIBRI score, HADS score) 9 months after NeuroAiD II™ (MLC901) treatment initiation with reference to baseline, compared to the placebo group.

3.2.2 Safety

The objective of the study is to assess safety of NeuroAiD II™ (MLC901) compared to placebo, following a course of 6-month treatment in adult patients with long-term cognitive impairment after mTBI.

Safety outcomes will include:

1. Cumulative rate of treatment-emergent adverse events (TEAEs) and adverse drug reactions (ADRs) during the study;
2. Cumulative rate of SAEs during the study;
3. Discontinuation of treatment (for any reason or due to AEs);
4. Clinically significant (assessed by the Investigator) deviations in the results of hematology, blood chemistry and urine laboratory tests compared to baseline (Detailed in Section 4.1.1).

Study safety endpoints are detailed in Table 3.2.

Table 3.2. Study safety outcomes and endpoints

Outcomes	Endpoints
Cumulative rate of TEAEs and ADRs during the study	• Number of TEAEs, recorded during the study period (270±30 days) in the NeuroAiD II™ group and in the Placebo group<br>• Number of ADRs, recorded during the study period (270±30 days, from Day 1 up to End-of-Study visit) in the NeuroAiD II™ group and in the Placebo group
Cumulative rate of SAEs during the study	Number of SAEs, recorded during the study period (270±30 days) in the NeuroAiD II™ group and in the Placebo group
Discontinuation of treatment (for any reason or due to AEs)	Number of patients who discontinued treatment in the NeuroAiD II™ group and in the Placebo group<br>• Due to AE<br>• For any reason
Clinically significant deviations in the results of hematology, blood chemistry and urine laboratory tests compared to baseline	Number of clinically significant (assessed by the Investigator) deviations in the results of haematology, blood chemistry and urine laboratory tests upon NeuroAiD II™/Placebo treatment completion (Day 180±14) in the NeuroAiD II™ group and in the Placebo group compared to baseline

Safety assessment is detailed in Section 8.

4 STUDY DESIGN

4.1 Description of Study Design

This is a randomized double blind, placebo-controlled, multi-center clinical trial. The overview of the study design is given below in Figure 4.1. This clinical trial will include 182 adults (aged between 18 and 65 years of age) who experienced a mild TBI in the past 1 to 12 months and experience cognitive functioning difficulties as indicated by a score of >30 on the Cognitive Failures Questionnaire (consistent with criteria in the pilot study (21)). Eligible participants will be randomized to receive either NeuroAiD II™ (MLC901) or matching placebo.

The study will consist of 3 periods:

- **Screening** (Day -14..-1)
- **Treatment period** (6 months, Day 1 – Day 180±14), during which patients will daily receive an oral dose of IP or placebo
- **Follow-up period** (3 months, Day 180±14 – Day 270±30), after which final efficacy assessment will be performed

The time period from Screening (Visit 1) to Visit 2 (Day 1, Randomization) will not exceed 14 days. If the patient leaves the study before study completion for any reason, then the Early-Termination visit is performed, which should include all procedures and assessments, planned for the End-of-Study visit on Day 270±30.

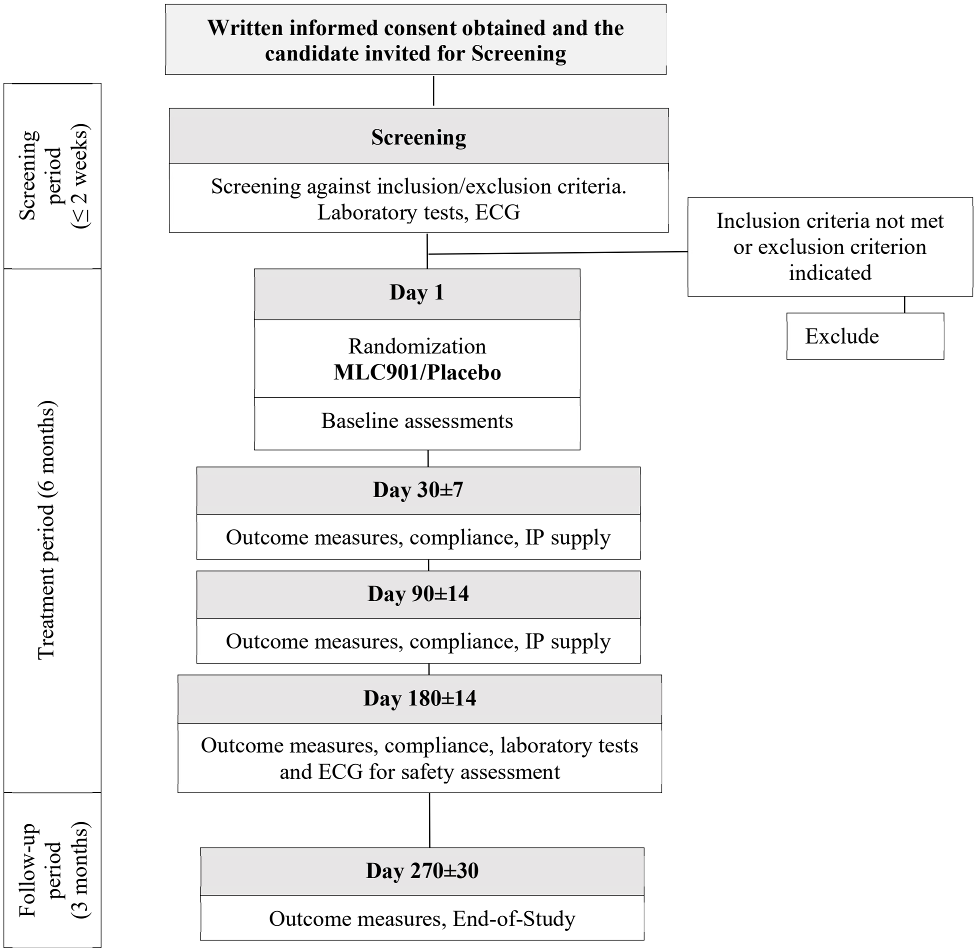

Figure 4.1. Study design.

4.1.1 Study procedures

Written Informed Consent

The Investigator will discuss all the necessary information about the study with the patient. The participant will have an opportunity to ask any questions about the study. Written consent must be obtained from all participants **prior to any study procedures**. The clinician/researcher obtaining consent should sign and date the consent form to state that the consent process has been completed according to the study protocol.

Two copies of the consent form should be signed and dated. One is to be retained by the participant. The other copy of the signed and dated information sheet and consent form must be left in the participant's file at the site.

The patient will receive the written information about:

- The investigational product and nature of this clinical study.
- Safety of the investigational product and risks for the patient.
- Terms of participation in the clinical study of the investigational product.
- Aim, objectives, and duration of the clinical study of the investigational product.
- Patient actions in case of unexpected effects of the investigational product on the patient's health.
- Terms of compulsory insurance of the patient's life and health.
- Confidentiality statement in relation to participation in the clinical study of the investigational product.

Screening procedure (Visit 1, Day -14..-1)

- The eligibility screening will include completion of the Cognitive Failures Questionnaire (CFQ). A score of >30 will be used to determine if the participant is experiencing cognitive impairment. CFQ will be performed after signing ICF at screening visit.
- If the subject has a CFQ score >30 and is ready to participate in the study, then the Investigator should check if the subject fulfils all other Inclusion and Exclusion Criteria.
- Collection of medical history, prior/concomitant medication (during the previous 12 months), demographic data (age, race), height, weight, BMI calculation, date/time of TBI and injury characteristics including worst recorded GCS score
- Pregnancy test
- Assessment of vital signs
- Physical examination
- Clinical Laboratory Tests
- ECG
- A meeting with the Investigator will be arranged for baseline assessment and randomization.

Randomization assessment (Visit 2, Day 1)

- Randomization
- Collection of baseline characteristics, medical history, prior/concomitant medication (Not necessary if complete information was obtained upon Screening)
- Efficacy outcomes to be assessed:
  - Cognitive functioning (complex attention, executive functioning, processing speed, memory (visual and verbal), reaction time) assessed with CNS-Vital Signs (CNS-VS)
  - Rivermead Post Concussion Symptoms Questionnaire (RPQ) - Questionnaire for Post-concussion symptoms
  - Health related quality of life (QOLIBRI) – Questionnaire for assessment of quality of life
  - Hospital Anxiety and Depression Scale (HADS) – Questionnaire for mood assessment

- Safety assessments:
  - Vital Signs
  - Physical examination
  - Collection of information about AEs

- Investigational product supply

Assessments after 1 and 3 months of treatment (Days 30±7 (Visit 3), 90±7 (Visit 4))

- Efficacy outcomes to be assessed:
  - CNS-Vital Signs (CNS-VS) - Computerized assessment for Cognition
  - Rivermead Post Concussion Symptoms Questionnaire (RPQ) -Questionnaire for assessment of post-concussion symptoms
  - Health related quality of life (QOLIBRI) – Questionnaire for assessment of quality of life
  - Hospital Anxiety and Depression Scale (HADS) – Questionnaire for mood assessment

- Safety assessments:
  - Vital Signs
  - Physical examination
  - Information about AEs

- Collection of prior/concomitant medication
- Compliance assessment
- Investigational product supply

<u>Assessment after 6 months of treatment (Day 180±14, Visit 5)</u>

- Efficacy outcomes to be assessed:
  - CNS-Vital Signs (CNS-VS) - Computerized assessment for Cognition
  - Rivermead Post Concussion Symptoms Questionnaire (RPQ) -Questionnaire for assessment of post-concussion symptoms
  - Health related quality of life (QOLIBRI) – Questionnaire for assessment of quality of life
  - Hospital Anxiety and Depression Scale (HADS) – Questionnaire for mood assessment

- Safety assessments:
  - Vital Signs
  - Laboratory tests
  - Physical examination
  - Information about AEs
  - ECG
  - Pregnancy test

- Collection of prior/concomitant medication
- Compliance assessment

<u>Assessment 3 months after treatment completion (End-of-Study, Visit 6, Day 270±30)</u>

- Efficacy outcomes to be assessed:
  - CNS-Vital Signs (CNS-VS) - Computerized assessment for Cognition
  - Rivermead Post Concussion Symptoms Questionnaire (RPQ) - Questionnaire for assessment of post-concussion symptoms
  - Health related quality of life (QOLIBRI) – Questionnaire for assessment of quality of life
  - Hospital Anxiety and Depression Scale (HADS) – Questionnaire for mood assessment

- Safety assessments:
  - Vital Signs
  - Physical examination
  - Information about AEs
- Collection of prior/concomitant medication
- Compliance assessment

Efficacy outcome assessments are detailed in Section 7. Safety outcome assessments are detailed below and in Section 8.

<u>Laboratory tests</u>

Standard hematology, blood biochemistry and urine tests will be conducted for safety monitoring at Screening (after eligibility is confirmed) and End-of-Study visits.

Hematology (approximately 5 mL blood sample):

- Haemoglobin (Hb)
- Hematocrit
- Total white blood cell and differential counts (Band cells and Segmented neutrophils, Eosinophils, Basophils, Monocytes, Lymphocytes)
- Red blood cell count
- Platelet count

Blood chemistry (approximately 5 mL blood sample):

- Total protein
- Aspartate Transaminase (AST)
- Alanine Transaminase (ALT)
- Glucose tests
- Creatinine
- Total Bilirubin
- Alkaline phosphatase

Standard urine test: specific gravity, color, pH, transparency, nitrites, glucose, protein, erythrocytes, epitheliocytes, cylinders, bacteria, leukocytes, mucus.

<u>Physical examination</u>

The examination will be carried out at every visit and will include:

- Lung auscultation
- Assessment of the visible mucous membranes of the upper respiratory tract
- Palpation and assessment of lymph nodes
- Evaluation of the abdominal organs and size of the liver and spleen (by palpation, without instrumental examination)
- Evaluation of the general condition, skin, eyes, musculoskeletal system, ENT, nervous system, gastro-intestinal tract, urinary track system.

<u>Vital signs assessment</u>

- Measurement of the body temperature
- Measurement of the respiration rate,
- Measurement of the blood pressure,

- Measurement of the heart rate

The assessment will be carried out at every visit. Subjects should be resting for at least 5 minutes prior to having vital sign measurements obtained in sitting position.

12-lead ECG

ECG will be done in supine position at Screening and End-of-study Visits. Subjects should be resting for at least 5 minutes prior to each ECG measurement.

Pregnancy test (in women with childbearing potential)

Pregnancy test will be performed upon Screening and at Visit 5 (upon treatment completion). It will be a standard urine test for β-HCG (test strip).

4.2 Description of the Measures Taken to Minimize/Avoid Bias

4.2.1 Randomization

After consenting to participate in the study and completing the baseline assessment, eligible participants will be randomized to receive either the NeuroAiD II™ (MLC901) or placebo using 1:1 stratified permuted block randomization. This approach will help to stratify by study center, time since injury [1-3 months/4-12 months] and gender. Time since injury between 3 and 4 months will be stratified to 1-3 months. Centralized stratified permuted block randomization will be done using Interactive Web Response System (IWRS). The randomization number will be generated by the system automatically after the investigator enters the patient data into the system. The study team, including the biostatistician, will be un-blinded only after database has been locked.

After the patient signs the ICF at the screening visit, he/she will be assigned the screening number that is assigned in the order of enrolment.

The randomization code will be assigned to each patient that passed the screening and was found eligible.

The randomization code of each patient will be recorded in the source records and the patient's Case Report Form (CRF). If the patient decides to leave the study or does not complete the study for any reason, then his randomization code should not be reused.

4.2.2 Blinding

This study is double-blind i.e. the patients and the investigators will not know the treatment assignments. Accordingly, the primary and secondary packaging of the IP and placebo will be blinded. The efficacy and safety outcomes will be defined based on the blinded analysis of data.

Information on unblinding is provided in Section 4.7.

4.3 Description of the Study Treatments

Table 4.1. Study treatments.

Investigational Drugs	Dose/Potency	Dose Frequency	Route of Administration	Treatment period	Use

NeuroAiD II™ (MLC901)	Standard dose of 2 capsules (0.4g/capsule)	3x/day for 6 months	orally	6 months	Study drug
Placebo	2 capsules (0.4g/capsule)	3x/day for 6 months	orally	6 months	Control

The experimental and control capsules will be presented in blister packs prepared by Moleac Pte Ltd and will have identical appearance.

All participants will continue to receive standard medical care, without any changes in medical treatment recorded.

4.4 Expected Duration of Subject Participation, and a Description of the Sequence and Duration of All Study Periods, Including Follow-up

The total duration of a patient's participation in this study will not exceed 314 days starting with the screening visit and finishing with the final End-of-Study visit or Early Termination visit.

The study will consist of 3 periods:

- **Screening** (Day -14 … Day -1)
- **Treatment period** (6 months, Day 1 – Day 180±14), during which patients will daily receive an oral dose of IP or placebo
- **Follow-up period** (3 months, Day 180±14 – Day 270±30), after which final efficacy assessment will be performed

4.5 Description of the "Stopping Rules" or "Discontinuation Criteria" for Individual Subjects, Parts of Study and Entire Study

The Sponsor may suspend the study at any time based on any reasons that include, but are not limited to, safety, ethics, or administrative issues. The Sponsor can terminate the study at any time if the aim and objectives of the study are not met. The Sponsor should inform the investigator or the leadership of the study center about the study suspension or termination in writing. If the study is suspended or terminated because of a safety issue, the Sponsor shall notify the investigators, regulatory authorities, and the Ethics Committees immediately.

4.6 Accountability Procedures for the Investigational Product(s), Including the Placebo and Comparator

Designated clinical staff will be responsible for receipt, storage, dispensing and accounting of study drugs according to Good Clinical Practice (GCP) and site's SOPs. The study drugs will be placed under appropriate storage conditions in an area with controlled access. Before dosing, the IP will be individually packed and labeled for each subject and period at the trial site and checked prior to IP administration.

The investigator must ensure that the Sponsor-supplied drug is used in accordance with the protocol and is dispensed only to subjects enrolled in the study. Drug supplies will be counted and reconciled at the site before being returned to the Sponsor. To document appropriate use of Sponsor-supplied drug, the Investigator will maintain records of all Sponsor-supplied drug delivery to the site and use by each subject. If any dispensing errors or discrepancies are discovered, the sponsor must be notified immediately.

The following information will be recorded by the Investigator: protocol number and title, name of Investigator, site identifier and number, description of Sponsor-supplied drugs, expiry date and amount dispensed, and the date and amount returned to the site by the subject. All study drug not returned to the site by a subject must be investigated by the site and appropriately documented.

To make the IP accountability process clearer, the Investigator will ask the subjects to fill the IP administration diary during the treatment period. The specially prepared IP administration diaries will be handled to subjects on Day 1.

4.7 Maintenance of Study Treatment Randomization Codes and Procedures for Breaking Codes

The double blinding will be maintained during the whole study. The randomization code of a patient can be broken in case of:

- Occurrence of an insured event if the assigned treatment must be revealed for qualification of the case
- Decision of the Sponsor
- Written requirement of the LEC that oversees the study

The patient's randomization code may be disclosed prematurely (during the clinical trial) in order to ensure patient safety, i.e. in the case of AE and in the case when information about the receiving treatment influences the further treatment tactics.

Unblinding is done using IWRS (Unblinding section). If possible, before breaking the code, the Investigator should inform the Study Sponsor and monitor about the need to disclose the code. The Investigator must make at least 3 attempts to contact the Study Sponsor / responsible monitor. In any case, after breaking the code the Investigator must inform the Sponsor and the monitor about it. The fact of code disclosure should be documented in the source documents.

A complete list of all the screening/randomization numbers used in the study and the corresponding therapy is a confidential document that is kept by an authorized representative and is used to monitor the conduct of the study.

The investigator must maintain the records with information on patient numbers. These records are stored in compliance with the rules applicable to confidential documents.

4.8 Identification of Any Data to be Recorded Directly on the Electronic CRF (eCRFs)

In this study, all data will be recorded in the primary documentation before entry into the CRF. At the end of each study participant visit, data of the visit including all scores will be entered into the CRF. CRF completion must be legible and complete. CRF must be kept current to reflect participant status at each phase during the course of the study. The investigator will be responsible for retaining all records pertaining to the study as specified in the contract.

5 SELECTION AND WITHDRAWAL OF SUBJECTS

This clinical trial will include 182 adults 18-65 years of age who experienced a mild TBI in the past 1 to 12 months and have cognitive functioning difficulties as indicated by a score of >30 on the Cognitive Failures Questionnaire (consistent with criteria in the pilot study). Eligible participants will be randomized to receive either NeuroAiD II™ (MLC901) or matching placebo.

5.1 Subject Inclusion Criteria

In order to participate in the study patient should meet the following inclusion criteria:

1. Patient with mild TBI, which occurred 1-12 months prior enrolment to the study. Mild TBI is defined as an external force from an incident causing injury to the brain and resulting in an altered level of consciousness. The mTBI diagnosis should be documented. Mild TBI is evidenced by any of the following:
    - best Glasgow Coma Score 13-15 as assessed on scene, on admission and over next 3 days
    - loss of consciousness for up to 30 minutes
    - dazed and confused at the time of injury or post-TBI amnesia of < 24 hours duration
2. Patient experiencing cognitive impairment following injury, determined by the Cognitive Failures Questionnaire score >30 (consistent with criteria in the pilot NeuroAiD II™ study (21)).
3. Adult male or female patients aged 18-65 years.
4. The patient signed an Informed Consent form (ICF) for participation in this study before initiation of study procedures.
5. The patient can understand all protocol requirements, perform the study procedures, and agree to all limitations specified in the protocol.
6. The patient agrees to use adequate contraception methods (for women of child-bearing potential)

5.2 Subject Exclusion Criteria

Patient will not be included in the study, if any of the following criteria is present:

1. Moderate or Severe TBI, determined by best Glasgow Coma Score of <13 (as assessed on scene, on admission and over next 3 days), or injury requiring neurosurgery (even if surgery was not done)
2. Co-existing severe co-morbidity, including end stage renal failure, spinal cord injury, significant substance abuse, severe liver disease, significant mental illnesses, diabetes requiring insulin injections, severe agitation, advanced cancer or other severe conditions with life expectancy of less than 5 years. The decision on significance of the comorbidity for inclusion/exclusion from current study is made by the Investigator.
3. Current participation in another clinical trial within 30 days
4. Women who are pregnant or who have a positive urine pregnancy test or breast-feeding
5. Not fluent in Russian language or have aphasia/dysphasia
6. No documented evidence of mTBI

7. Known allergy to MLC901/NeuroAiD II™ or any of its component ingredients
8. Other medical condition which in the opinion of the Principal Investigator would place undue risk on the patient if included in the trial or likely to interfere with NeuroAiD II™
9. History of psychic (including depressive) disorders, physical and other factors that do not allow for adequate self-assessment of one's behaviour and for compliance with the protocol requirements, including history of psychiatric disorders.
10. Use of hormonal contraceptives, either oral or implant.

5.3 Contraception

Females in this study will be of child-bearing potential (WOCBP) and not of child-bearing potential. Females considered to be not of child-bearing potential, are:

- Surgically sterile (i.e. both ovaries removed, uterus removed or bilateral tubal ligation) and/or
- Post-menopausal (no menstrual period for at least 12 consecutive months without any other medical cause).

A female subject is considered to be a WOCBP if she is physically capable of becoming pregnant and does not fulfil any of the above-mentioned criteria

Prior to participating in the study, all patients should be informed about the importance of preventing an unwanted pregnancy while participating in the study and the potential risk factors for an unwanted pregnancy.

Subjects and their sexual partner must use an acceptable single- or double method of contraception from the Screening visit until completion of the study. The possibility of known or unknown drug interactions between the hormonal contraceptives and the investigational medicinal product always exist. Moreover, there is a possibility that the investigational product may lessen the effectiveness of a hormonal contraceptive agent. Hence female subjects with childbearing potential using hormonal contraceptives will not be included in the study.

Acceptable single- or double-methods of contraception are presented below:

Table 5.1. Acceptable Effective Methods of Contraception

Single-method	Double-method (must use at least two of them)	
• Surgically sterile;<br>• Postmenopausal;<br>• Remain abstinent.	• Diaphragm;<br>• Cervical cap;<br>• Vaginal sponge;<br>• Non-hormonal IUD	• Condom;<br>• Spermicide;<br>• Vasectomy.

The study subjects should consent to use the methods of contraception described above throughout the study period or to complete abstinence during the study period.

5.4 Subject Withdrawal Criteria

1. The patient withdraws the Informed Consent.

2. Patient's pregnancy: patients will be recommended to use effective contraception methods during the study, however if pregnancy occurs during the study, the IP/placebo treatment and other study procedures concerning the pregnant patient are cancelled, patient's pregnancy will be monitored up to pregnancy completion if the patient agrees.
3. Any medical condition of the patient that requires withdrawal of that patient from the study, as judged by the Investigator.
4. Patient lost to follow-up.

The investigator can withdraw a subject from the study if any of the withdrawal criteria are met. Patients are free to withdraw from the study at any time upon request, without any consequence. Patients should be listed as having withdrawn consent only when they no longer wish to participate in the study and no longer authorize the investigators to make efforts to continue collection of the outcomes. Every effort should be made to encourage patients to remain in the study for the duration of the planned outcome assessments. The patients should be told that their data are important for the scientific research, even after they discontinue the drug product.

The participant should no longer be contacted regarding the study after notification of withdrawal.

A subject will be considered lost to follow-up if he or she fails to appear for a follow-up assessment and cannot be contacted with good effort. These efforts will be documented in the subject's record. If not a single dose of the IP/comparator was taken by the patient, and he/she left the study for any reason, then further follow-up is cancelled, and his/her data are not included in the statistical analysis.

If the patient decides to leave the study or does not complete the study for any reason, then his randomization code should not be reused.

Unless the patient withdraws consent, those who discontinue the IP early remain in the study, if possible, for further collection of outcomes. The reason for discontinuation of the IP should be documented in the CRF.

The Investigator submits the data on the withdrawn patients to the Sponsor, local Principal Investigator and the monitor of the study within 24 hours in a report that includes the patient's status at the end of the study, as follows:

1. *Completed the study.*
2. *Did not complete the study because of:*
   - Any condition of the patient that requires withdrawal of that patient from the study, as judged by the investigator
   - The patient wished to leave the study
   - The patient lost to follow-up
   - Pregnancy
   - Other reasons (specify)

5.5 Replacement of the Subjects

This study does not provide for the replacement of withdrawn study participants.

5.6 Follow-up of the Withdrawn Patients

The subjects that were withdrawn from the study because of an AE/SAE will be followed until the AE/SAE is resolved or the patient is stabilized.

5.7 Strategies for Recruitment and Retention

5.7.1 Recruitment

It is anticipated that patients with mTBI will attend the participating clinical centers. Clinicians based within recruiting centers will be asked to identify potential participants from their patient lists, based on the eligibility criteria, and inform them about the study. Recruitment efforts will also include dissemination of information about this trial to other medical professionals/clinical centers.

5.7.2 Retainment

As recommended by Corrigan et al (34) to minimize the risk of attrition multiple contact details for an alternative contact person will also be obtained if nominated by the participant. Participants will be asked as to their preferred method of contact.

A reminder phone-call or text will be sent before each appointment to help prevent missed appointments. All Investigators in the study will be trained on the importance of establishing rapport with study participants and how to convey why the study is important to participants. Participating subjects will be reminded of subsequent visits.

6 TREATMENT OF SUBJECTS

6.1 Treatment(s) to be Administered

The IP/Placebo will be supplied in kits. Each IP kit contains 3 sachets, 1 sachet contains 5 blisters, 1 blister contains 12 capsules.

In total, 182 subjects will be included in the study, therefore 7 IP kits are necessary for a treatment course. Maximum 10 sites are planned to participate in the study. Taking into account stratified block randomization by 2 binary stratification factors (4 strata per site totally) and possible variative block size, we assume that 12 kits per site will not be used due to possible imbalance within all strata and 12 kits per site should be reserved during the study for newly opened blocks. Thus, 1514 IP/Placebo kits are needed for study population, 1666 IP/Placebo kits needed for the study conduction (10% buffer to avoid transfer between sites).

6.1.1 Investigational Product

Table 6.1. Description of IP.

**NeuroAiD II™ (MLC901)**	
**Trade name:**	NeuroAiD II™
**International Non-proprietary Name (INN):**	NeuroAiD II™ (MLC901)
**Manufacturer/Marketing Authorization Holder:**	Moleac Pte Ltd,<br>Helios #09-08, 11 Biopolis Way, Singapore 138667
**Dosage form:**	Capsule 400 mg (size 0)
**Dosage:**	Recommended treatment is 2 capsules orally, 3 times a day (i.e. 6 capsules per day). Standard treatment is 12 weeks. MLC901 capsules could be:<br>- swallowed as such with water<br>- opened and drunk once powder has been diluted in a sufficient amount of water of room temperature*<br>**Clumping of the powder content may be observed due to the inherent nature of the powder extract. This does not indicate deterioration of quality if used within the specified shelf life.*
**Composition (per 1 capsule)**	See Table 6.2.
**Appearance:**	The capsule cap is dark blue and capsule body is light blue in color. The capsule size is 0. The powder content is brown to dark brown colour and has some agglomerates.
**Storage terms:**	To be stored in a cool dry place not exceeding 30°C

**Package and labelling:**	Capsules are gelatin-free. They are made up of Hypromellose (hyrodypropyl methycelluose HPMC) which is permitted as a food additive for human consumption in accordance with 21 CFR 172.874. The capsules are packed in blister made of PVDC/PE/PVC multilayer clear blister front and 25-micron aluminum backing with 12 capsules per stip. Every 5-blister strip (i.e. 60 capsules) is prepacked into an aluminum sachet before being placed in a box.

Table 6.2. Composition of MLC901.

**Active Ingredients**				
	**Ingredients (Latin names)**	**Parts Used**	**Quantity* (mg) per capsule**	**Equivalent extract per capsule (mg)**
1	*Radix Astragali*	Dried root	800	111.5
2	*Radix et Rhizoma Salviae miltiorrhizae*	Dried root/rhizome	160	22.3
3	*Radix Paeoniae rubra*	Dried root	160	22.3
4	*Rhizoma chuanxiong*	Dried rhizome	160	22.3
5	*Radix Angelicae sinensis*	Dried root	160	22.3
6	*Flos Carthami*	Dried flower	160	22.3
7	*Semen persica*	Dried ripe seed	160	22.3
8	*Radix polygalae*	Dried root	160	22.3
9	*Rhizoma Acori tatarinowii*	Dried rhizome	160	22.3

**Excipients**			
	**Name**	**Functions**	**Quantity (mg) per capsule**
1	Dextrin	Bulking agent	57
2	Maltodextrin	Bulking agent	51
3	Magnesium stearate	Lubricant	2
**Ingredients of Capsule Shell**			

	Name	Functions	Quantity (mg) per capsule
1	Brilliant blue FCF-FD&C Blue 1	Capsule colorant	0.0819
2	Titanium dioxide	Capsule-opacifier	1.895
3	Hypromellose	Capsule- structure	89.79
4	Water	Solvent	4.23

6.1.2 Placebo

Table 6.3. Description of Placebo.

**Placebo for NeuroAiD II™ ()**	
**Placebo manufacturer:**	Poli Medical Company Pte Ltd
**Dosage form:**	Capsule 400 mg (size 0)
**Composition (per 1 capsule)**	Dextrin, Chocolate Brown, Cameral, Magnesium stearate
**Appearance:**	The capsule cap is dark blue and capsule body is light blue in color. The capsule size is 0. The powder content is brown to dark brown colour and has some agglomerates.
**Storage terms:**	Store in cool dry place ≤ 30 °C
**Package and labelling:**	Capsules are gelatin-free. They are made up of Hypromellose (hyrodypropyl methycelluose HPMC) which is permitted as a food additive for human consumption in accordance with 21 CFR 172.874. The capsules are packed in blister made of PVDC/PE/PVC multilayer clear blister front and 25-micron aluminum backing with 12 capsules per stip. Every 5-blister strip (i.e. 60 capsules) is prepacked into an aluminum sachet before being placed in a box.

6.2 Medications/Treatments Permitted (Including Rescue Medication) and Not Permitted Before and/or During the Study

All participants will continue to receive standard medical care, without any changes in medical treatment recorded except for the medications which should be restricted during the study. The medications to be restricted in this study include drugs that can potentially affect the study results, e.g., most importantly, patients' performance upon CNS-VS testing.

These medications to be restricted during the study include drugs with nootropic, metabolic or sedative effects, or those which can exert such effects based on the Investigator's opinion. The Investigator should prescribe any of these drugs only upon consultation with the Sponsor and after the Sponsor approves the use of such medication.

6.3 Procedures for Monitoring Subject Compliance

Subjects will be supplied with IP kits. Each IP kit contains 3 sachets. One sachet contains 5 Blisters, one blister contains 12 capsules.

The scheme of patients supply with IP will be the following:

- 2 kits will be given on Visit 2 (Day 1) - 1 kit for administration and 1 kit reserved),
- 2 kits will be given on Visit 3 (Day 30±7), and
- 3 kits – will be given on Visit 4 (Day 90±14).

One reserved kit should always be left at the patient's.

If a participant misses one dose, he/she will be advised to take the dose as soon as he/she remembers or with the next dose. If more than one dose is missed (e.g. missed a whole day) the participant will be advised to take one dose as soon as they remember and then to continue treatment as usual. No more than 8 capsules should be taken in one 24-hour period. Participants will be asked not to discard any capsules that they have not taken. Subjects will bring the dispensed study medication IP kits (including sachets and blisters) to each visit. Any leftover capsules (e.g. capsules that participants had forgotten to take) will be collected by the Investigator at the end of each time period (at 1, 3 and 6 months). The Investigator will check subject compliance by counting the number of returned capsules at specified time points and record the number of missed capsules. All cases of lost or damaged capsules should be documented. Treatment compliance will be calculated by the investigator at Visits 2, 3, and 4 (1,3,6 months). Patients will be given study diaries to be filled with information about actual drug intake by the patient.

Subjects will be considered to be in non-compliance with study medication if they miss >20% of doses required for the visit period, or if they take more than 120% of the doses for the treatment period since last visit. If these criteria for noncompliance are met, a protocol deviation should be reported. Any detected overdose should be reported on CRF.

In case of a compliance deviation, all subjects should be reinstructed about the dosing requirements during study contacts.

7 ASSESSMENT OF EFFICACY

7.1 Specification of the Efficacy Parameters

Efficacy outcomes:

- Cognitive functioning parameters, determined by CNS-VS computer testing, for following cognitive domains:
  - complex attention,
  - other cognitive domains: executive functioning, processing speed, memory (visual and verbal), reaction time
- post-concussion symptoms
- quality of life
- levels of anxiety and depression

in adult patients with long-term cognitive impairment following mTBI compared to baseline after 6 months of NeuroAiD II™ (MLC901) treatment with reference to placebo-treated adult patients with long-term cognitive impairment following mTBI.

2.In case of any improvement in any of the above parameters upon completion of NeuroAiD II™ (MLC901) treatment: to assess changes in improved parameters 3 months after treatment completion with reference to the placebo-treated group.

7.2 Methods and Timing for Assessing, Recording, and Analyzing of Efficacy Parameters

Outcome Assessments

Efficacy outcomes, corresponding assessment methods and terms are summarized in Table 7.1.

Table 7.1. Efficacy outcomes, assessment methods and terms.

Outcomes	Assessment method	Assessment terms
**Primary**		
Change in Complex attention score in adult patients with long-term cognitive impairment following mTBI, who were randomized to receive NeuroAiD II™ (MLC901) compared to patients randomized to placebo.	Change from baseline in the complex attention values, determined by Central Nervous System Vital Signs (CNS-VS) system	after 6 months of NeuroAiD II™ (MLC901) treatment
**Secondary**		
Changes in the scores for the following cognitive domains: executive functioning, processing speed, memory (visual and verbal) and reaction time, in adult patients with long-term cognitive impairment following mTBI, who were randomized to receive NeuroAiD II™ (MLC901), compared to patients randomized to placebo.	Change from baseline in values, determined by CNS-VS system	after 6 months of NeuroAiD II™ (MLC901) treatment
Change in the total score for post-concussion symptoms in adult patients with long-term cognitive impairment following mTBI, who were randomized to	Change from baseline in values, determined by Rivermead Post Concussion	after 6 months of NeuroAiD II™ (MLC901) treatment

receive NeuroAiD II™ (MLC901) compared to patients randomized to placebo.	Symptoms Questionnaire (RPQ)	
**Exploratory**		
Change in the total score for the quality of life, in adult patients with long-term cognitive impairment following mTBI, who were randomized to receive NeuroAiD II™ (MLC901) compared to patients randomized to placebo.	Change from baseline in values, determined by Health-related quality of life questionnaire (QOLIBRI)	after 6 months of NeuroAiD II™ (MLC901) treatment
Change in the total score for levels of anxiety and depression, in adult patients with long-term cognitive impairment following mTBI, who were randomized to receive NeuroAiD II™ (MLC901) compared to patients randomized to placebo.	Change from baseline in values, determined using Hospital Anxiety and Depression Scale (HADS)	after 6 months of NeuroAiD II™ (MLC901) treatment
Changes, compared to baseline, in the analysed parameters (complex attention, executive functioning, processing speed, memory (visual and verbal), reaction time, post-concussion symptoms, quality of life, the levels of anxiety and depression) in adult patients with long-term cognitive impairment following mTBI with reference to the placebo group.	Change from baseline in values, determined by CNS-VS system, RPQ, QOLIBRI, HADS	9 months of NeuroAiD II™ (MLC901) treatment initiation (i.e. 3 months after NeuroAiD II™ (MLC901) treatment completion)

The assessments conducted by the Investigator are expected to take approximately 30-40 minutes. All assessments will be completed in a suitable location (e.g. quiet, private room) where distractions can be minimized as much as possible.

The details of outcome measures are described in Table 4.1.

The Sponsor/CRO will be available to answer questions the Investigator may have. The Investigators will be able to forward any queries that they are unable to answer to Sponsor/CRO for advice.

Table 7.2. Outcome measures.

**Demographic, Baseline and Injury Characteristics**	
Demographic and Baseline characteristics	Age, gender, ethnicity, employment status (pre and post injury: employee/worker/self-employed/unemployed/retired/not clear), educational level (education highest level: secondary level/specialized secondary education/higher education/academic degree), marital status (married or living with partner/not married or living with partner), substance use (alcohol, drugs: never/rarely/regularly/every day), smoking status (Y/N), use of dietary supplements, history of any previous TBI other than the one causing current condition (name, severity, date, duration), current medication use (name of medication (INN), dose, regimen and route of administration, date of beginning and end if applicable, indication), comorbid conditions (diagnosis, duration).
TBI Injury Characteristics	Date of injury, mechanism of injury, severity of injury (GCS score), additional injuries
CFQ score at Screening	CFQ score value

**Primary Outcome**	
Complex attention	*CNS Vital Signs* is a computerized neurocognitive test battery that was developed as a routine clinical screening instrument (27). It is comprised of seven tests:<br>• verbal and visual memory,<br>• finger tapping,<br>• symbol digit coding,<br>• the Stroop Test,<br>• a test of shifting attention and<br>• the continuous performance test.<br><br>The CNS-VS scale is detailed in this Section below.
**Secondary Outcomes**	
Executive functioning, processing speed, memory (visual and verbal) and reaction time	*CNS Vital Signs*
Post Concussive Symptoms	*Rivermead Post Concussion Symptoms Questionnaire (RPQ) (29, 30)*<br>This questionnaire asks people about the occurrence of 16 post-concussive symptoms such as headaches, fatigue and restlessness. Participants are to state whether the extent to which they experience each symptom, in comparison to before their accident, on a 5 point scale ranging from 0 (not experienced) to 4 (severe problem). The first 3 items relate to early onset symptoms and individual item scores are summed to give a summary score between 0-12. Items 4-16 are indicative of more severe post concussive symptoms and are summed to reveal a total score between 0-52. The two scales have revealed good test-re-test reliability and adequate external construct validity (29,30)
**Outcomes within exploratory analysis**	
Health Related Quality of Life	*Quality of Life after Brain Injury (QOLIBRI)(32)*<br>The questionnaire consists of 2 parts. The first part assesses satisfaction with health-related quality of life (HRQoL) and is composed of 6 overall items and 29 items allocated to 4 subscales: thinking, feelings, autonomy and social aspects. The second part, devoted to "bothered" questions, is composed of 12 items in 2 subscales: negative feelings and restrictions. The 6 subscales meet standard psychometric criteria. In addition, 2 items evaluate medical-oriented aspects. The QOLIBRI showed good construct validity in the TBI group (31).
Mood	*Hospital Anxiety and Depression Scale (HADS)* (32,33)<br>This scale has been widely used for assessing levels of anxiety and depression in patients with medical problems such as TBI. The scale consists of 14 questions that ask participants how they have been feeling over the past week. There are two subscales (anxiety and depression) and subscale scores range between 0-21 (0-7 normal, 8 to 10 mild, 11-14 moderate and 15-21 representing a severe level of anxiety/depression). The HADS has been found to have high internal consistency[34] and is sensitive to treatment related change.

CNS-VS is a reliable and validated instrument of TBI studies, and notably for studies including mild TBI patients. Details about this testing system are available online at https://www.cnsvs.com.

During the CNS-VS testing, a patient undergoes 7 cognitive tests, after which, based on the results of these tests, the system automatically calculates scores for a number of cognitive domains: complex attention, neurocognitive index, executive functioning, memory (visual and verbal), reaction time, psychomotor speed, processing speed, simple attention, motor speed, composite memory, cognitive flexibility.

Psychometric properties of CNS-VS have been extensively studied, including test-retest reliability, sensitivity, concurrent validity with other psychometric tests and discriminant validity in different clinical settings (various levels of TBI severity, mild cognitive impairment, depression, ADHD, etc) (46,47,48).

Discriminant validity of CNS-VS is supported by studies of patients with mild cognitive impairment and dementia, post-concussion syndrome and severe traumatic brain injury, ADHD (treated and untreated) and depression (treated and untreated) (27). The tests in CNS-VS are also sensitive to malingerers and patients with conversion disorders. The psychometric characteristics of the tests in the CNS VS battery are very similar to the characteristics of the conventional neuropsychological tests upon which they are based (27,28). CNS-VS is free from practice effect and therefore is suitable for use as a serial assessment measure.

In a study of Gualtieri, CNS-VS complex attention score was compared between patients who had recovered from mild brain injury/concussion (with loss of consciousness less than 20 minutes and/or transient post-traumatic amnesia (less than 24 hours) and normal patients (46-48). CNS-VS Complex Attention score was assessed at 100.41 in the subgroup on normal patients, as compared to an average of 92.8 in the mild brain injury/concussion subgroup (46-48). These results suggest that a difference in complex attention score in the range of 8-10 is clinically important and distinguish normal subjects from subjects with some level of post brain injury impairment.

For each subject at each assessment (1, 3, 6, 9 months after treatment initiation) scores in each of the above standard scales (CNS-VS, HADS, QOLIBRI, RPQ) will be collected and used for statistical analysis (Detailed in Section 9).

8 ASSESSMENT OF SAFETY

8.1 Specification of Safety Parameters

Safety outcomes will include:

- Cumulative rate of treatment-emergent adverse events (TEAEs) and adverse drug reactions (ADRs) during the study;
- Cumulative rate of SAEs during the study;
- Discontinuation of treatment (for any reason or due to AEs);
- Clinically significant (assessed by the Investigator) deviations in the results of hematology, blood chemistry and urine laboratory tests compared to baseline (Detailed in Section 4.1.1).

8.2 Methods and Timing for Assessing, Recording, and Analyzing Safety Parameters

The corresponding safety endpoints are*:*

1. Number of TEAEs, recorded during the Study period (Day 1 – Day 270±30) in the NeuroAiD II™ group and in the Placebo group
2. Number of ADRs, recorded during the Study period (Day 1 – 270±30 days) in the NeuroAiD II™ group and in the Placebo group
3. Number of SAEs, recorded during the Study period (Day 1 – 270±30 days) in the NeuroAiD II™ group and in the Placebo group
4. Number of patients who discontinued treatment in the NeuroAiD II™ group and in the Placebo group
   - Due to AE
   - For any reason
5. Number of clinically significant (assessed by the Investigator) deviations in the results of haematology, blood chemistry and urine laboratory tests upon NeuroAiD II™/Placebo treatment completion (Day 180±14) in the NeuroAiD II™ group and in the Placebo group compared to baseline (data of laboratory tests and ECG performed at Screening)

At each study visit, the Investigator will ask patients about their well-being and assess whether any subjective AEs have occurred. The AEs, TEAEs, SAEs and ADRs will be recorded starting from the first IP dose intake (Day 1). Subjects may report to the Investigator the AEs occurring at any other time during the study. All subjects experiencing AEs, whether considered associated with the use of the study drug or not, must be monitored until resolution or stabilization.

Procedures for physical examination, vital signs assessment, 12-lead ECG and laboratory tests were detailed in Section 4. The physical examination results, as well as any changes that have occurred since the last inspection should be described in the primary documents and CRF. Data of physical examination, laboratory tests, vital signs and ECG data will be categorized as "normal", "abnormal, clinically not significantly" or "clinically significant abnormal". The clinically significant deviations in results of laboratory or instrumental assessment and physical examination data, vital signs and ECG data should be registered as AEs in eCRF.

8.3 Procedures for Eliciting Reports of and for Recording and Reporting Adverse Event and Concurrent Illnesses

8.3.1 Definitions

8.3.1.1 Adverse Event

An Adverse Event (AE) is any untoward medical occurrence in a patient or clinical investigation subject administered a pharmaceutical product and that does not necessarily have a causal relationship with this treatment. An AE can therefore be any unfavorable and unintended sign (including an abnormal laboratory finding), symptom, or disease temporally associated with the use of a medicinal (investigational) product, whether or not related to the medicinal (investigational) product.

Treatment Emergent Adverse Event (TEAE) – undesirable event not present prior to medical treatment, or an already present event that worsens either in intensity or frequency following the treatment.

8.3.1.2 Adverse Drug Reaction

Adverse Drug Reaction (ADR) − in the preapproval clinical experience with a new medicinal product or its new usages, particularly as the therapeutic dose(s) may not be established, all noxious and unintended responses to a medicinal product related to any dose should be considered adverse drug reactions. The phrase "responses product" means that a causal relationship between a medicinal product and an adverse event is at least a reasonable possibility, i.e., the relationship cannot be ruled out.

Regarding marketed medicinal products: A response to a drug that is noxious and unintended and that occurs at doses normally used in man for prophylaxis, diagnosis, or therapy of diseases or for modification of physiological function.

8.3.1.3 Serious Adverse Event

Serious Adverse Event (SAE) − any untoward medical occurrence that at any dose:

1. Results in death;
2. Is life-threatening;

   NOTE: The term “life threatening” refers to an event in which the subject was at risk of death at the time of the event; it does not refer to an event that hypothetically might have caused death if it were more severe.

3. Requires inpatient hospitalization or prolongation of existing hospitalization

   NOTE: In general, “hospitalization” means that a given patient has been admitted (usually at least one night) in a medical facility or emergency room for follow-up and / or treatment that could not have been provided during the visit to the doctor or on an outpatient basis. Complications that occur during hospitalization are considered AEs. If a complication lengthens hospitalization or meets other criteria for a SAE, it is considered serious. If there is doubt as to whether the “hospitalization” actually took place, or whether it was necessary, the AE should be considered a SAE. **Scheduled hospitalization to treat a pre-existing condition that did not worsen during this study is not considered a SAE**.

4. Results in persistent or significant disability/incapacity;
5. Is a congenital anomaly/birth defect.
6. Is an important medical event that satisfies any of the following:
   - May require intervention to prevent items 1 through 5 above;
   - May expose the subject to danger, even though the event is not immediately life threatening or fatal or does not result in hospitalization.

SUSAR is any SAE where a causal relationship with the study product is at least reasonably possible but is not listed in the Investigator Brochure (IB), Package Insert, and/or Summary of Product Characteristics.

8.3.2 Evaluation of severity

The severity of each AE will be graded (mild, moderate, severe, life-threatening, death) according to the Common Terminology Criteria for Adverse Events (CTCAE) Grading (Severity) Scale version 5 (https://ctep.cancer.gov/protocoldevelopment/electronic_applications/docs/CTCAE_v5_Quick_Reference_8.5x11.pdf)

8.3.3 Causality assessment

The relationship of each AE to study medication will be assessed using the following categories:

Related:	An AE that follows a reasonable temporal sequence from administration of a drug (including the course after withdrawal of the drug), or for which possible involvement of the drug cannot be ruled out, although factors other than the drug, such as underlying diseases, complications, concomitant drugs and concurrent treatments, may also be responsible.
Not Related:	An AE that does not follow a reasonable temporal sequence from administration of a drug and/or that can reasonably be explained by other factors, such as underlying diseases, complications, concomitant drugs and concurrent treatments.

The relationship of each ADR to study medication will be assessed using the following categories:

Conditional / Unclassified	Event or laboratory test abnormality with more data for proper assessment needed, or additional data under examination
Unlikely related	An event or laboratory test abnormality, with a time to drug intake that makes a relationship improbable (but not impossible), underlying diseases, complications, concomitant drugs and concurrent treatment provide plausible explanation
Possibly related	Event or laboratory test abnormality, with reasonable time relationship to drug intake, which could also be explained by disease or other drugs. Information on drug withdrawal may be lacking or unclear
Probably related	Event or laboratory test abnormality, with reasonable time relationship to drug intake, which is unlikely to be attributed to disease or other drugs. Response to IP withdrawal is clinically reasonable. Rechallenge is not required.

Definitely related	Event or laboratory test abnormality, with plausible time relationship to drug intake which cannot be explained by disease or other drugs. The response to withdrawal is plausible (pharmacologically, pathologically).<br><br>Event definitive pharmacologically or phenomenologically (ie, an objective and specific medical disorder or a recognized pharmacologic phenomenon). Rechallenge satisfactory, if necessary.
Not Applicable	Report suggesting an adverse reaction, which cannot be judged because information is insufficient or contradictory, data cannot be supplemented or verified

8.3.4 Documentation and reporting of adverse events

All AEs will be documented in the AE page of the CRF, whether or not the investigator concludes that the event is related to the drug treatment. The following information will be documented for each event:

- Event term (preferably, diagnosis).
- Dates of the beginning and the end of AE.
- Frequency: intermittent, continuous.
- Severity: CTCAE grades
- Causal relationship between the event and administration of study drug or study procedure (not related, conditional, unlikely related, possibly related, probably related, definitely related, not applicable).
- Action concerning study drug (drug withdrawn, dose not changed, dose reduced/increased, unknown, not applicable).
- Outcome of event (recovered, recovering, not recovered, resolved with sequelae, fatal, unknown).
- Seriousness (serious, non-serious).

Information on TEAEs and SAEs will be received by all members of the Study team.

8.3.4.1 SAE reporting

When an SAE occurs through the AE collection period it should be reported according to the following procedure:

As soon as the Investigator is aware of SAE, he/she:

- immediately registers information in patient's medical file,
- immediately (or within 24 hours of first onset or notification of the event) completes and signs the SAE form and sends it to Atlant Clinical by fax or e-mail (e-mail: p7501.safety@atlantclinical.com, contact information will also be given in the Investigator study file):

Concerning any safety aspects, the Atlant Clinical medical monitor should be contacted:

Name: Irina Mazheykina

Position: Medical Monitor

Tel.: 7 (495) 628 3802

Fax: +7 (495) 628 3633

Mobile: +7 (926) 358 9670

e-mail: irina.mazheykina@atlantclinical.com, p7501.safety@atlantclinical.com.

In the SAE form the information should be completed as fully as possible but contain at a minimum:

- A short description of the event and the reason why the event is categorized as serious.
- Subject identification number.
- Investigator’s name.
- Name of the study drug

In case of SAE, the Sponsor must also be notified either by the Investigator or by Atlant Clinical within 24 hours after the Investigator/CRO is aware of the SAE:

Name: Htar Htar Nwe, MBBS

Position: Senior Medical Advisor

Tel.: +65 6211 3710 Ext 182

Tel. (mobile): +65 8533 0651

e-mail: htarhtar.nwe@moleac.com

Any follow-up information on SAE should be notified within 24 hours via the SAE form (the procedure should be the same as described above). If a non-serious adverse event becomes serious, or if an adverse event becomes a suspected fatal/life-threatening adverse reaction, the procedure should be the same as described above.

For SAEs, the Investigator will send to Atlant Clinical anonymized copies of additional medical documents with relevant information on the event in the same way as described above. Upon SAE completion the final SAE report form should be filled and sent to Atlant Clinical by e-mail: p7501.safety@atlantclinical.com or by Fax: +7 (495) 628 3633

Atlant Clinical will immediately transmit to the Sponsor the forms and documents received from the Investigator (within 24 hours after the information was received by CRO).

8.3.1 Reporting Adverse Events to Subjects

Subjects will be informed of any severe AEs or SAEs that they experience as part of their participation in this trial.

8.3.1.1 Overdose reporting

Cases of overdose with any medication without manifested side effects are not considered AEs, but will be documented in an Overdose section of the eCRF. Following information should be included (but not limited to): whether the overdose was suspected or confirmed, whether or not involving study medication, overdose signs and symptoms, action taken, medication/antidote given or not. Any manifested side effects will be considered AEs.

8.3.1.2 Reporting of Pregnancy

While pregnancy is not considered to be an AE or SAE, any pregnancy complication or elective termination of a pregnancy will be reported as an AE or SAE. A spontaneous abortion is always considered to be an SAE and will be reported as such. In situation when pregnancy occurs during study participation, it should be reported to Atlant Clinical medical monitor (for Sponsor

informing) within 24 hours using the procedure described for SAE reporting. Pregnancy should be recorded on the eCRF and source documents, a specialized Pregnancy Form should be filled.

If a female study participant is pregnant, then further IP intake and all study procedures should be cancelled. The female patient who becomes pregnant during the study will be followed using a Pregnancy Form until the outcome of the pregnancy is known.

Any pregnancies in the partner of a male study participant during the study should also be recorded following authorization from the subject's partner, withdrawal of the male study participant from the study is not required. Pregnancy of the male study participant's partner should also be followed using Pregnancy Form.

For both female subjects and partners of male subjects information about SAEs that occurred during pregnancy with the mother and/or fetus or child should be transmitted to the Sponsor also in case of Study discontinuation.

Pregnancy outcome, including any premature termination, must be reported to the Sponsor. An evaluation after the birth of the child should also be conducted.

8.3.2 Responsibility of the Sponsor

The sponsor will be responsible for reporting all suspected unexpected serious adverse reactions (SUSARs) to regulatory authorities, Investigators and Ethics Committee in accordance with national regulations of Russian Federation. Relative to the first awareness of the event by/or further provision to the Sponsor, SUSARs will be submitted to the regulatory authorities as expedited report within 7 days for fatal and life-threatening events and 15 days for other serious events. Relevant follow-up information will be submitted to a safety report as soon as the information is available. Upon request from regulatory authority, the Sponsor will submit any additional data or information that the agency deems necessary, as soon as possible, but in no case later than 15 calendar days after receiving the request. SAEs that are not SUSARs will be reported to the regulatory authority at least annually in a summary format which includes all SAEs.

The Sponsor will also prepare expedited reports for other safety issues which might alter the current benefit-risk assessment of the study drug or that would be sufficient to consider changes in the study drug administration or in the overall conduct of the trial. The study site also will forward a copy of all expedited reports to its Ethics Committee in accordance with local regulations.

8.4 Type and Duration of the Follow-up of Subjects After Adverse Events

All AEs and SAEs should be followed up until resolution or stabilization. The timelines and procedure for SAE follow-up reports are the same as those for the initial report.

The Investigator must ensure that follow-up of the participant is appropriate to the nature of the event, and that it continues until resolution if deemed necessary.

Any secondary worsening (i.e. from a non-serious adverse event to a serious adverse event) should be notified to the Sponsor without undue delay. Any new information (diagnosis, intensity, measures taken, causality or outcome) regarding an adverse event already reported will be documented in the source document and eCRF.

If the adverse event has not resolved at the participant's final visit in the study, the participant must be followed up suitably and any information on the outcome of the event will be recorded.

8.5 Safety data review by DSMB

An independent Data Safety and Monitoring Board (DSMB, detailed in Section 11.6) will be established and meet at regular intervals as defined by the DSMB Charter after commencing the trial participants recruitment to ensure safety of the intervention by monitoring blinded safety data collected in this Study. The DSMB will meet prior to the start of the Study and at the projected middle of the study recruitment period, it may also meet on an *ad hoc* basis if necessary.

9 STATISTICS

9.1 Description of the Statistical Methods to be Employed

An overall comprehensive statistical analysis plan (SAP) for this clinical trial (including trial design) will be developed and finalized after trial initiation and before data lock/unblinding.

Sponsor/CRO will be responsible for ensuring all data is entered accurately into the study database. All analyses will be undertaken by Sponsor/CRO following completion of recruitment and data collection phases, all data will be entered into a secure database and after the data cleaning process the database will be locked for analysis. All assessment measures will be scored according to standardized procedures. Creation of derived variables or conversion of variables (log-transformation, Z-scores etc.) will be determined for each sub-analysis. These will be documented in the SAP for each of these smaller sub-analyses.

9.1.1 Descriptive analysis of demography and baseline data

Data on demographics, laboratory results, vital signs, physical examination will be presented using the following descriptive statistics: number of observations, percentages, mean, standard deviation, median, quartiles, minimum and maximum values.

These baseline differences and differences in outcomes at 1, 3, 6 and 9-month follow-up will be summarized using means, standard deviations, medians, quartiles, minimums and maximums.

Baseline value for all parameters is defined as the most recent estimate before first drug intake.

9.1.2 Efficacy analysis

Primary analysis will employ ITT population, sensitivity analyses will be performed using ITT population with LOCF imputations and PP population.

Primary efficacy endpoint: Change in complex attention score, determined using Central Nervous System Vital Signs (CNS-VS) computer cognitive testing system, after 6 months of treatment compared to baseline in the group of patients receiving NeuroAiD II™ (MLC901), compared to the placebo group.

For primary endpoint analysis for each patient, the difference in complex attention scores, determined using CNS-VS computer testing, after 6 months of therapy compared to baseline, will be calculated: the change in complex attention score compared to baseline. Mixed effects model (PROC MIXED) will be used with adjustments for baseline and potential covariates. All timepoints will be used for the model. The participant and site will be used as the random effects. Model selection will be undertaken with each outcome using standard selection heuristics. Covariates will be selected based on improving the overall efficiency of the model. Regardless, baseline and age, gender, time since injury (1-3 months/4-12 months) and study center will be included as covariates in the mixed effects model. Descriptive statistics will be calculated to change in complex attention in each group, as well as mean values obtained by the method of least squares from the mixed effects model.

Superiority of MLC901 over placebo will be claimed if

H0: $LSM_T >= LSM_R$ is rejected and thereafter

H1: $LSM_T < LSM_R$ is accepted at significance level of 5%,

where LSM_T is a Least Square Mean of Change from Baseline of Complex attention score after 6 months of treatment assessed by the mixed model repeated measures analysis in the MLC901 group and LSM_R is a Least Square Mean of Change from Baseline of Complex attention score

after 6 months of treatment assessed by the mixed model repeated measures analysis in the Placebo group (lower score of Complex attention is better).

Secondary efficacy endpoints

- Changes in the scores for the following cognitive domains: executive functioning, processing speed, memory (visual and verbal) and reaction time, determined by CNS-VS system, after 6 months of treatment compared to baseline, in the NeuroAiD II™ (MLC901) group, compared to the placebo group.
- Change in the Rivermead Post Concussion Symptoms Questionnaire (RPQ) total score, after 6 months of treatment, compared to baseline, in the NeuroAiD II™ (MLC901) group compared to the placebo group.

Exploratory efficacy endpoints

- Change in the Health-related quality of life questionnaire (QOLIBRI) total score after 6 months of treatment, compared to baseline, the NeuroAiD II™ (MLC901) group compared to the placebo group.
- Change in the Hospital Anxiety and Depression Scale (HADS) score after 6 months of treatment, compared to baseline, in the NeuroAiD II™ (MLC901) group compared to the placebo group.
- Changes, compared to baseline, in the analyzed parameters (complex attention, executive functioning, processing speed, visual and verbal memory, reaction time, RPQ score, QOLIBRI score, HADS score) 9 months after NeuroAiD II™ (MLC901) treatment initiation with reference to baseline, compared to the placebo group. .

The same approach as for primary endpoint will be used for analysis of secondary efficacy endpoints and exploratory endpoints, and completely similar hypotheses are be formulated as for the analysis of the primary endpoint. Mixed effects models will be used to analyze the change in values from baseline, adjusted for covariates and baseline estimates. No sensitivity analysis will be performed for secondary endpoints. No adjustments will be made for multiple comparisons, hypotheses will be tested hierarchically, hypothesis testing by secondary endpoints will be taken into account if statistically significant differences between groups for the primary endpoint are confirmed.

9.1.3 Safety analysis

Safety analysis will be performed in the safety population. Regardless of the reason for the completion of the study, the data of all patients who received at least one IP/Placebo dose according to the assignments will be included in the safety analysis.

Adverse events will be coded using MedDRA dictionary. Incidences of TEAEs/SAEs will be calculated for assessment of AEs. Incidences of adverse events reported during the study will be presented as number of patients with AE in total and in each treatment group. Also, number of AEs per each severity category and per causal relationship with the study drug will be presented.

Incidences of adverse events will be compared between the IP and the Placebo group. The proportion of patients **with at least one adverse event** will be compared in the NeuroAiD II™ group and in the placebo group.

Vital Signs, Laboratory tests, Physical examination, ECG, Pregnancy test results will be tabulated by visits. Descriptive statistics by visit and for change from baseline will be summirized. Number of clinically significant (assessed by the Investigator) deviations in the results of haematology, blood chemistry and urine laboratory tests upon NeuroAiD II™/Placebo treatment completion

(Day 180±14) in the NeuroAiD II™ group and in the Placebo group compared to baseline will be tabulated.

No formal statistical testing is assumed for safety analysis.

9.2 Number of Subjects Planned to be Enrolled

The primary endpoint for the trial is change in complex attention score, determined using CNS-VS computer cognitive testing system, after 6 months of treatment compared to baseline in the group of patients receiving NeuroAiD II™ (MLC901), compared to the placebo group. Primary Analysis: the primary analysis will compare the mean change in the complex attention score, determined by CNS-VS testing, after 6 months of therapy after 6 months of treatment, compared to baseline, between the MLC901 group and the placebo group by analysis of variance in the ITT population.

In a study of Gualtieri (46-48), CNS-VS complex attention score was compared between patients who had recovered from mild brain injury/concussion (with loss of consciousness less than 20 minutes and/or transient post-traumatic amnesia (less than 24 hours) and normal patients. CNS-VS Complex Attention score was assessed at 100.41 in the subgroup on normal patients, as compared to an average of 92.8 in the mild brain injury/concussion subgroup (46,47,48). These results suggest that a difference in complex attention score in the range of 8-10 is clinically important and distinguish normal subjects from subjects with some level of post brain injury impairment.

The sample size for this study is estimated based upon a previous study in 78 patients evaluating the effect of MLC-901 (NeuroAiD II) versus placebo using CNS-VS as main efficacy assessment (21). Results obtained at 6 months for CNS-VS complex attention in (21) are displayed in the following table:

Table 9.1. Complex Attention score: Mean (SD) (extract from the Supplementary Table 1 in (21)

Group	MLC-901 (n=36)	Placebo (n=42)
Baseline	83.86 (22.06)	85.43 (16.52)
After 6 months	94.18 (15.29)	82.31 (21.56)

While the scores are very similar at baseline, there is a difference of about 12 points at 6 months between placebo and MLC-901 groups, in favor of the active treatment. Based upon these results, sample size for current study has been calculated in order to provide 80% power to detect a difference of 10 points in CNS-VS Complex Attention score change from baseline after 6 months (at a 2-sided alpha level 5%). The standard deviation used in this calculation for the parameter is estimated to be 20.

Study is powered to claim superiority of MLC901 over Placebo by primary endpoint for following statistical hypothesis: H0: $LSM_T >= LSM_R$, H1: $LSM_T < LSM_R$, where LSM_T is a Least Square Mean of Change from Baseline of Complex attention score after 6 months of treatment assessed by the mixed model repeated measures analysis in the MLC901 group and LSM_R is a Least Square Mean of Change from Baseline of Complex attention score after 6 months of treatment assessed by the mixed model repeated measures analysis in the Placebo group (lower score of Complex attention is better).

PASS 14 software (Scenario: two sample T-test assuming equal variance) was used to estimate the required sample size. Target power – 80%. Alpha – 0.05. Proportion of subjects in groups – 1:1.

Hypotheses: H0: MT ≥MR, H1: MT < MR, where MT is an arithmetical mean of change of Complex attention score after 6 months of treatment, compared to baseline, in the MLC901 group, and MR is an arithmetical mean of change from baseline of Complex attention score after 6 months of treatment in the placebo group (lower score of Complex attention is better).

An estimate of 20 was obtained from the BRAINS pilot study (21) for standard deviation of change in complex attention from baseline to 6 months, 128 subjects (64 per arm) are required to detect a clinically meaningful difference of -10 in the mean changes in complex attention between MLC901 and placebo arms with 80% power at a two-sided 5% significance level. Sensitivity analysis: 1) power is equal 29% if the mean difference of primary estimate is 5 and power is equal 99% if the mean difference of primary estimate is 20; 2) power is equal 15% if the standard deviation increases up to 30, so some uncertainty in the standard deviation estimate may cause insufficient actual power for comparison.

The sample size is increased to 182 randomized subjects (91 per arm) to allow for 30% dropouts. Considering very high screening failure rate up to 364 subjects can be screened.

9.3 Level of Significance to be Used

The two-sided significance level of 0.05 (5%) will be applied for all statistical tests.

9.4 Criteria for the Termination of the Study

The sponsor may temporarily or permanently stop the study at one or all sites for safety, ethical, protocol compliance or other reasons. If such a need arises, the Sponsor will take steps to notify the center in advance. If a center or study is temporarily or permanently closed, the Investigator must promptly inform the local Ethics Committee. The Sponsor must promptly inform the regulatory authorities of the temporary or permanent closure of the center.

9.5 Procedure for Accounting for Missing, Unused, and Spurious Data

No missing data will be substituted for primary analysis. LOCF imputation method will be employed for sensitivity analysis. Ambigous data will be clarified via queries.

9.6 Procedures for Reporting Any Deviation(s) from the Original Statistical Plan

Statistical methods could be replaced if it contributes to a more correct and informative analysis. Any deviation(s) from the original statistical plan will be described and justified in the Statistical Analysis Report (SAR) and in the Clinical Study Report (CSR).

9.7 Selection of Subjects to be Included in the Analyses

Following populations will be analyzed:

1. Intention-to-treat population (ITT), which includes every subject who is randomized according to randomized treatment assignment.
2. Per-Protocol population (PP): includes all subjects from ITT population who complete all assessments needed for primary endpoint calculation except subjects with significant protocol deviations.
3. Safety population: Patients who received at least one IP/Placebo dose, according to the prescribed treatment.

10 DIRECT ACCESS TO SOURCE DATA/DOCUMENTS

Source data is the information contained in the original medical records and their certified copies that describe the results of clinical observations, examinations and other activities and allow reproducing the course of a clinical study and evaluate its quality. Source data is contained in the source records (originals or their certified copies) and in electronic form if an electronic data collection system is used.

The Investigator has to authorize monitoring of the study (by the authorized representative of the Sponsor and/or CRO), audits (by the authorized representative of the Sponsor or the company authorized by the Sponsor to conduct audits of the study center) and inspections by the regulatory authorities with direct access to source data and source records including primary documentation and to provide his own time and the time of his staff to work with the monitor, Sponsor's representatives, auditors and regulatory inspectorates to discuss findings and other issues.

11 QUALITY CONTROL AND QUALITY ASSURANCE

11.1 Site Monitoring Visits

Monitoring visits to the study site will be made periodically during the study to ensure that all aspects of the protocol are followed. Source documents will be reviewed for verification of data recorded on the eCRFs. Source documents are defined as original documents, data, and records. The investigator and institution guarantee access to source documents by the Sponsor or CRO and by LEC.

All aspects of the study and its documentation will be subject to review by the Sponsor or designee (as long as blinding is not jeopardized), including but not limited to the Investigator's Binder, study medication, subject medical records, informed consent documentation, documentation of subject authorization to use personal health information (if separate from the informed consent forms), and review of eCRFs and associated source documents.

11.2 Protocol deviations

The Investigator should not deviate from the protocol, except where necessary to eliminate an immediate hazard to study subjects. Should other unexpected circumstances arise that will require deviation from protocol-specified procedures, the Investigator should consult with the Sponsor or designee to determine the appropriate course of action. There will be no deviation from the Inclusion or Exclusion criteria.

The site should document all protocol deviations in the subject's source documents and in the protocol violation record form held in the study master file. In the event of a significant deviation, the site should notify the Sponsor.

11.3 Quality Assurance Audits and Regulatory Agency Inspections

The study site also may be subject to quality assurance audits by the Sponsor or designees. In this circumstance, the Sponsor-designated auditor will contact the site in advance to arrange an auditing visit. The auditor may ask to visit the facilities where laboratory samples are collected, where the medication is stored and prepared, and any other facility used during the study. In addition, there is the possibility that this study may be inspected by regulatory agencies, including those of foreign governments. If the study site is contacted for an inspection by a regulatory body, the Sponsor should be notified immediately. The Investigator and institution guarantee access for quality assurance auditors to all study documents.

11.4 Data Monitoring and Quality Control

NeuroAiD II™ and placebo are provided by Moleac Singapore Pte Ltd and manufactured under the recognized principles of current Good Manufacturing Practice (GMP). This includes that regular audits are conducted by independent health authorities. The GMP standards apply to manufacturing procedures and processes, raw materials, laboratory testing, packaging and distribution. Batches of NeuroAiD II™ are analysed thoroughly by third-party independent laboratories to confirm compliance with international standards, ensuring utmost safety for patients.

Several sources of bias are possible in a study of this type. To overcome these possible biases, particular procedures have been developed. Adherence to these procedures will be monitored at regular meetings of the CRO/Sponsor and the Steering Committee.

- A Data and Safety Monitoring Board will be established including a statistician.
- Due to the nature of the self-report assessments the Investigator will be instructed to rely primarily on information provided by participants.
- Data and logic checks will be conducted to ensure accuracy of the documentation.
- Substantial bias could occur if a low response is achieved from participants, and if there were systematic differences in information about outcomes between cases. These problems will be overcome as far as possible by monitoring responses, ensuring repeated attempts at contact, and allowing flexible convenient assessment times.
- The study will be monitored to ensure data quality and to facilitate entry of participants into the study. An independent Data Safety and Monitoring Board (DSMB) will be established and meet at regular interval as defined by the DSMB Charter (detailed in Section 11.6). Regular monitoring will be done by the Study Manager of CRO and report shared with the Sponsor.

All revisions to the global protocol will be done by the Sponsor and Steering committee (if required) and relevant Ethics Committees will be approached to approve the amendments. If an Amendment substantially alters the study design or increases the potential risk to the participant: (1) the consent form will be revised and submitted to the Ethics Committee(s) for review and approval or favorable opinion; (2) the revised form will be used to obtain consent from participants currently enrolled in the study if they are affected by the Amendment; and (3) the new form will be used to obtain consent from new participants prior to enrolment.

Prior to commencing recruitment of the study participants, the CRO will organize Investigator meeting (Virtual) for training the Investigators and site staff. Training sessions will include training on administering the tests and questionnaires and principles of ICH good clinical practice. Regular meetings with the Investigators and site staff will be maintained by the project team of the CRO to ensure integrity and conduct of the trial according to the Study Protocol.

The study manager of the CRO will provide yearly reports on study progress until completion of the study/termination or discontinuation of the study to the Sponsor (Moleac), Ethics Committees and/or regulatory authorities as per local requirements.

11.5 Steering Committee

The role of the Steering Committee (SC) is to provide oversight of the conduct of the clinical trial and ensures the study is conducted to the required standards. The SC will oversee scientific and practical aspects of the study. The SC will ensure that the study runs in a manner that is safe for study participants and also provides appropriate safety and efficacy data to the Sponsor (Moleac) and to the study investigators. In its safety role, the SC will work with the Data and Safety Monitoring Board (DSMB) established for this clinical trial.

Specific responsibilities of the Steering Committee include, but are not limited to, the following:

- Overall supervision of the clinical trial;
- Monitor recruitment rates and take steps to reduce and minimize deviations from the study protocol, recommend protocol amendments if needed;
- Periodic review of the progress of the clinical trial towards milestones to maximize the likelihood of completion within the agreed time period;
- Work in conjunction with the Data Safety Monitoring Board (DSMB) and review reports on participant safety and data from DSMB;

- Amicably resolve any issues within the research team and the Sponsor regarding the study's data management and monitoring procedures or any recommendations for modifications to the Study Protocol;
- Approve publication and authorship plans suggested by Scientific Advisors and PIs;
- Consider any new external information relevant to the Study.

The SC has the scientific responsibility for this clinical trial and works in conjunction with DSMB who recommends continuation, modification or termination of the study due to safety considerations.

The SC will consist of at least 5 members, including Chair of the SC. The Sponsor (Moleac) will approve all nominations for the SC. SC membership will consist of persons with strong scientific expertise and track record in the field of Traumatic Brain Injury and no conflict of interest. At least three SC members, including the SC Chair or the Chair's designee, will constitute a quorum. The PI will be allowed to attend SC meetings.

The SC will meet prior to the start of the study, and every 6 months thereafter, depending on progress of recruitment and study conduct. The SC may also meet on an *ad hoc* basis as necessary, and/or at the request of the Sponsor (Moleac).

Prior to study initiation a Charter describing in detail SC composition, responsibilities and processes will be signed by the Sponsor (Moleac) and the PI.

11.6 Data Safety Monitoring Board (DSMB)

An independent Data Safety and Monitoring Board (DSMB) will be established and meet at regular intervals as defined by the DSMB Charter after commencing the trial participants recruitment to ensure safety of the intervention and monitoring Study progress. The role of the Data and Safety Monitoring Board (DSMB) is to safeguard the interests of study participants and to monitor blinded safety data collected in this clinical trial.

Specific responsibilities of the DSMB include, but are not limited to, the following:

- To review the DSMB charters and make recommendation for any changes
- To agree to and evaluate the safety monitoring procedures of this clinical study as proposed by the Study team;
- To make recommendations to the SC regarding any necessary modifications to the Study Protocol and/or to the safety monitoring procedures of this Study;
- To review safety data blinded to treatment allocation, including case reports of suspected ADRs, SUSARs and to request further analyses as necessary;
- Conclude each data review with recommendations to whether the Study should continue without changes, be modified, be analyzed as interim, be terminated, or other optional approaches
- To protect the confidentiality of the study data and the DSMB discussions.

Every effort will be made to reach a consensus within the DSMB and with the Principal Investigator (PI). In case of disagreement on recommended modifications, the SC will make the final decision.

The DSMB will consist of at least three members, with at least one biostatistician, an expert in the field of TBI, and a medical expert with no conflict of interest.

At least 2 DSMB members are required to constitute a quorum.

Prior to study initiation a Charter describing in detail DSMB composition, responsibilities and processes will be signed by the Sponsor and the PI.

12 ETHICS

This study will be conducted in accordance with the ethical principles that have their origins in the Declaration of Helsinki. The study protocol and any subsequent amendment(s) will be submitted to the Ethics Committee. The study will be conducted in compliance with the protocol, GCP regulations and the applicable regulatory requirements.

The regulatory application or submission for regulatory approval will be made by the Sponsor as required by law of Russian Federation. Regulatory considerations concerning current study are detailed in Section 2.6.

12.1 Approval by Ethics Committee

The Sponsor or designee will supply relevant documents for submission to the respective Ethics Committee (LEC, Local Ethics Committee) or for the protocol's review and approval. This protocol, the Investigator's Brochure, a copy of the Informed Consent Form, and, if applicable, subject recruitment materials and/or advertisements and other documents required by all applicable laws and regulations, must be submitted to a LEC for approval. The written approval by the LEC of the protocol and subject informed consent must be obtained and submitted to the Sponsor or designee before commencement of the study (i.e., before shipment of the sponsor-supplied drug or study specific screening activity). The approval must refer to the study by exact protocol title, number, and version date; identify versions of other documents (e.g., Informed Consent Form) reviewed; and state the approval date.

Sites must adhere to all requirements stipulated by the respective ethics committee.

12.2 Subject Information and Informed Consent

Written consent documents will embody the elements of informed consent as described in the Declaration of Helsinki and the ICH Guidelines for GCP and will be in accordance with all applicable laws and regulations. The Informed Consent Form and subject information sheet will describe the planned and permitted uses, transfers, and disclosures of the subject's personal and personal health information for purposes of conducting the study. The Informed Consent Form and the subject information sheet further explain the nature of the study, its objectives, and potential risks and benefits, as well as the date informed consent is given. The Informed Consent Form will detail the requirements of the participant and the fact that he or she is free to withdraw at any time without giving a reason and without prejudice to his or her further medical care.

The Investigator is responsible for the preparation, content, and LEC approval of the Informed Consent Form. The Informed Consent Form and subject information sheet must be approved by both the LEC and the Sponsor prior to use.

The Informed Consent Form and subject information sheet (if applicable) must be written in a language fully comprehensible to the prospective subject. It is the responsibility of the investigator to explain the detailed elements of the informed consent form and subject information sheet (if applicable) to the subject. Information should be given in both oral and written form whenever possible and in the manner deemed appropriate by the LEC.

The subject must be given ample opportunity to: (1) inquire about details of the study and (2) decide whether or not to participate in the study. If the subject determines he or she will participate in the study, then the Informed Consent Form must be signed and dated by the subject, or the subject's legally acceptable representative, at the time of consent and prior to the subject entering into the study. The subject or the subject's legally acceptable representative should be instructed to sign using their legal names. The investigator must also sign and date the informed consent form at the time of consent and prior to subject entering into the study.

Once signed, the original Informed Consent Form and subject information sheet will be stored in the Investigator's site file. The investigator must document the date the subject signs the informed consent in the subject's medical record. Copies of the signed Informed Consent Form and subject information sheet shall be given to the subject.

All revised Informed Consent Forms must be reviewed and signed by relevant subjects in the same manner as the original informed consent. The date the revised consent was obtained should be recorded in the subject's medical record, and the subject should receive a copy of the revised Informed Consent Form.

12.3 Confidentiality of patient data

The confidentiality of all participants will be protected. Electronic data will be securely stored at the location specified by the Sponsor/CRO. Hard copy data must be stored in a securely locked cabinet at each site in accordance with ethics and country requirements. No participant identifiers will be present on any files transmitted to any committee or any clinical center. No one outside of the study team will have access to records identifying subjects' names at any time.

The data will be stored with the CRO during the study and will be shared with the sponsor (Moleac) upon study completion. In CRFs or any other documents submitted to the Sponsor, the subjects will not be identified by their names, but by an identification code, that was assigned to the subjects, and dates of birth.

Documents not for submission to the Sponsor, i.e. the confidential subject identification code, original consent forms and source records will be maintained by the Investigator in strict confidence.

13 DATA HANDLING AND RECORD KEEPING

The physician participating in the study collects data in accordance with the protocol and any guidelines established by the study center. Each participating Investigator collects Patient Information Sheets with an Informed Consent Form and maintains patient anonymity. In addition, the physician should report any potential serious adverse reactions in accordance with local standards for pharmacovigilance used in standard medical practice.

The Investigator must be familiar with the ICH GCP guidelines and conduct research in accordance with them.

<u>Data logging</u>

The Investigator or his representative is responsible for registering study data in the CRF provided by the Sponsor. The Investigator must ensure the accuracy of the data entered into the CRFs.

The Investigator must ensure that the CRF is completed in a timely manner and must provide the Sponsor's representative with periodic access to patient's records and all study-related materials. The frequency of monitoring visits will be determined by factors such as study design, frequency of patient visits, and rate of enrollment at the center. To confirm that the ICH GCP study is conducted in accordance with the regulatory requirements and the study protocol, as well as the authenticity, accuracy and completeness of the data, the study monitor will review the CRFs and other documents related to the study and verify the primary data.

Upon completion of the study, the Sponsor's representative must visit the study center for a study termination visit. In this case, the collection of the necessary documentation will be carried out.

<u>Storage of basic study documents</u>

The main documents, as defined by the ICH GCP, include signed protocol and all subsequent amendments, copies of completed CRFs, signed patient information sheet forms with Informed Consent Forms for all patients who have given consent, case histories, diaries and other primary documentation, Ethics Committee approvals and all correspondence with the Ethics Committee, including approved documents, drug reporting records, study correspondence, and a list of patient names and addresses.

The Investigator must retain copies of key documents for a period specified by the ICH GCP and regulatory requirements.

The Investigator must inform the Sponsor of the location of key documents and contact the Sponsor for approval before destroying any of them. The investigator should take steps to prevent accidental destruction of these documents before the required time limit.

14 FINANCING AND INSURANCE

Financing: The Sponsor, financing this study, is Moleac Pte Ltd.

Insurance: The Study is insured by AlfaStrakhovanie JSC.

In accordance with Article 44 of the Federal Law "On medicinal products circulation" No. 61-FZ, before the start of the study, the procedure for insuring the health of patients participating in a clinical trial of a medicinal product is carried out. In the event of harm to the patient's health related to the clinical trial, the Insurance company through which the Sponsor entered into the insurance contract undertakes to reimburse all costs for the necessary medical examination and treatment, the need for which arises as a result of the direct effect of the study drug and/or medical manipulations used according to the study protocol.

The Sponsor i.e. Moleac Pte Ltd will indemnify each locality against claims bought against them by or on behalf of study participants (or their dependents) for personal injury to participants arising out of the administration and/or use of the NeuroAiD II™ supplement. Moleac will maintain appropriate insurance with respect to all localities and activities, including its indemnity obligations. This insurance is to be evidenced by a certificate of currency to be made available on request to trial localities.

15 PUBLICATION POLICY

Use of information

Investigators are obliged to use the information received from the Sponsor only for the purposes of this study, and not to use it for other purposes without the written permission of the Sponsor.

The Investigator must provide the Sponsor with the complete results of the study and all data obtained by the Investigator during the study. During the course of the study and after its completion, information about the study can only be provided by the Sponsor only, except as required by law and regulations.

Publication of results

The PI or investigator shall be free to make scholarly publications on the findings of the project upon approval of the Steering Committee and the Sponsor. To ensure compliance with confidentiality, the Sponsor will provide the other with a copy of any proposed disclosures related to the project in advance of the proposed disclosure. There will be no constraints applied to examination of thesis and other publications. If necessary, publication of a thesis may be subject to approval and agreed timeline by the Sponsor.

16 REFERENCES

1. Maas AIR, Menon DK, Adelson PD, Andelic N, Bell MJ, Belli A, et al. Traumatic brain injury: Integrated approaches to improve prevention, clinical care, and research. *The Lancet Neurology*. 2017; 16: 987-1048
2. Langlois JA, Rutland-Brown W, Wald MM. The epidemiology and impact of traumatic brain injury: A brief overview. *Journal of Head Trauma Rehabilitation*. 2006; 21: 375-378
3. Hyder AA, Wunderlich CA, Puvanachandra P, Gururaj G, Kobusingye OC. The impact of traumatic brain injuries: A global perspective. *Neurorehabilitation*. 2007; 22: 341-353
4. Feigin VF, Theadom A, Barker-Collo SL, Starkey N, McPherson K, Kahan M, et al. Incidence of traumatic brain injury in new zealand: A population-based study. *The Lancet Neurology*. 2013; 12: 53-64
5. Halliwell B. Reactive oxygen species and the central nervous system. *Journal of Neurochemistry*. 1992; 59: 1609-1623
6. McMahon P, Hricik A, Yue JK, Puccio AM, Inoue T, Lingsma HF, et al. Symptomatology and functional outcome in mild traumatic brain injury: Results from the prospective track-tbi study. *Journal of Neurotrauma*. 2013;31 Epub ahead of print
7. Theadom A, Parag V, Dowell T, McPherson K, Starkey N, Barker-Collo S, et al. Persistent problems 1 year after mild traumatic brain injury: A longitudinal population study in new zealand. *British Journal of General Practice*. 2016; 66: 16-23.
8. Cramer SC. Repairing the human brain after stroke: I. Mechanisms of spontaneous recovery. *Ann Neurol*. 2008; 63: 272-287
9. Iverson GL. Outcome from mild traumatic brain injury. *Curr Opin Psychiatry*. 2005; 18: 301-317
10. Lannsjo M, af Geijerstam JL, Johansson U, Bring J, Borg J. Prevalence and structure of symptoms at 3 months after mild traumatic brain injury in a national cohort. *Brain injury*. 2009; 23: 213-219
11. Zumstein MA, Moser M, Mottini M, Ott SR, Sadowski-Cron C, Radanov BP, et al. Long-term outcome in patients with mild traumatic brain injury: A prospective observational study. *Journal of Trauma*. 2011; 7: 120-127
12. Chen C, Venketasubramanian N, Gan RN, Lambert C, Picard D, Chan BP, et al. Danqi piantang jiaonang (dj), a traditional chinese medicine, in poststroke recovery. *Stroke; a journal of cerebral circulation*. 2009; 40: 859-863
13. Heurteaux C, Gandin C, Borsotto M, Widmann C, Brau F, Lhuillier M, et al. Neuroprotective and neuroproliferative activities of neuroaid (mlc601, mlc901), a chinese medicine, in vitro and in vivo. *Neuropharmacology*. 2010; 58: 987-1001
14. Quintard H, Borsotto M, Veyssiere J, Gandin C, Labbal F, Widmann C, et al. Mlc901, a traditional chinese medicine protects the brain against global ischemia. *Neuropharmacology*. 2011; 61: 622-631
15. Bavarsad Shahripour R, Shamsaei G, Pakdaman H, Majdinasab N, Nejad EM, Sajedi SA, et al. The effect of neuroaid (mlc601) on cerebral blood flow velocity in subjects' post brain infarct in the middle cerebral artery territory. *Eur J Intern Med*. 2011; 22: 509-513
16. Harandi AA, Abolfazli R, Hatemian A, Ghragozlee K, Ghaffar-Pour M, Karimi M, et al. Safety and efficacy of mlc601 in iranian patients after stroke: A double-blind, placebo-controlled clinical trial. *Stroke Res Treat*. 2011; 2011: 721613
17. Young SH, Zhao Y, Koh A, Singh R, Chan BP, Chang HM, et al. Safety profile of mlc601 (neuroaid) in acute ischemic stroke patients: A singaporean substudy of the chinese medicine neuroaid efficacy on stroke recovery study. *Cerebrovascular Diseases*. 2010; 30: 1-6

18. Kong KH, Wee SK, Ng CY, Chua K, Chan KF, Venketasubramanian N, et al. A double-blind, placebo-controlled, randomized phase ii pilot study to investigate the potential efficacy of the traditional chinese medicine neuroaid (mlc 601) in enhancing recovery after stroke (tiers). *Cerebrovascular Diseases*. 2009; 28: 514-521
19. Siow CH. Neuroaid in stroke recovery. *Eur Neurol*. 2008; 60: 264-266
20. Gan R, Lambert C, Lianting J, Chan ES, Venketasubramanian N, Chen C, et al. Danqi piantan jiaonang does not modify hemostasis, hematology, and biochemistry in normal subjects and stroke patients. *Cerebrovascular Diseases*. 2008; 25: 450-456
21. Theadom A, Barker-Collo S, Jones KM, Parmar P, Bhattacharjee R, Feigin VL. MLC-901 (neuroaid ii) for cognition after traumatic brain injury: A pilot randomized clinical trial. Eur J Neurol. 2018; 25: 1055-e1082.
22. Al Fauzi A, Prihastomo KT, IRanuh IGMAR, Apriawan T, Wahyuhadi J, Parenrengi MA, Turchan A, Bajamal AH, Notobroto HB. Clinical outcomes of MLC601 (NeuroAiDTM) in traumatic brain injury: a pilot study. Brain Sci. 2020; 10(60): 1-9.
23. Broadbent DE, Cooper PF, FitzGerald P, Parkes KR. The cognitive failures questionnaire (cfq) and its correlates. *British Journal of Clinical Psychology*. 1982; 21: 1-16
24. Pocock SJ. *Clinical trials a practical approach*. Chichester: John Wiley & Sons; 1983.
25. Munivenkatappa A, Agrawal A, Shukla DP, Kumaraswamy D, Devi BI. Traumatic brain injury: Does gender influence outcomes? *Int J Crit Illn Inj Sci*. 2016; 6: 70-73
26. Papaliagkas V, Papantoniou G, Tsolaki M, Moraitou D. Self-report instruments of cognitive failures as screening tools for subjective cognitive impairment in older adults. *Hellenic journal of nuclear medicine*. 2017; 20 Suppl: 58-70
27. Gualtieri CT, Johnson LG. Reliability and validity of a computerized neurocognitive test battery, cns vital signs. *Archives of Clinical Neuropsychology*. 2006; 21: 623-643
28. Papathanasiou A, Messinis L, Georgiou VL, Papathanasopoulos P. Cognitive impairment in relapsing remitting and secondary progressive multiple sclerosis patients: Efficacy of a computerized cognitive screening battery. *ISRN neurology*. 2014; 2014: 151379
29. King NS, Crawford S, Wenden FJ, Moss NE, Wade DT. The rivermead post concussion symptoms questionnaire: A measure of symptoms commonly experienced after head injury and its reliability. *Journal of Neurology*. 1995; 242: 587-592
30. Eyres S, Carey A, Gilworth G, Neumann V, Tennant A. Construct validity and reliability of the rivermead post-concussion symptoms questionnaire. *Clinical Rehabilitation*. 2005; 19: 878-887
31. Von Steinbuechel N, Petersen C, Bullinger M. Assessment of health-related quality of life in persons after traumatic brain injury - development of the qolibri, a specific measure. 2005:43-49
32. Zigmond AS, Snaith RP. The hospital anxiety and depression scale. *Acta Psychiatrica Scandinavica*. 1983; 67: 361-370
33. Bjelland I, Dahl AA, Haug TT, Neckelmann D. The validity of the hospital anxiety and depression scale. An updated literature review. *Journal of psychosomatic research*. 2002; 52: 69-77
34. Corrigan JD, Harrison-Felix C, Bogner J, Dijkers M, Terrill MS, Whiteneck G. Systematic bias in traumatic brain injury outcome studies because of loss to follow-up. *Arch Phys Med Rehabil*. 2003; 84: 153-160.
35. Common terminology criteria for adverse events (ctcae). Version 5.0. Published: November 27, 2017. U.S. Department of health and human services, national institutes of health, national cancer institute https://ctep.Cancer.Gov/protocoldevelopment/electronic_applications/docs/ctcae_v5_quick_reference_8.5x11.Pdf accessed 4 september 2018.
36. Investigator's Brochure for MLC901 v. 2.0 dated 30 November 2020 (Moleac Pte Ltd).

37. Quintard H, Lorivel T, Gandin C, Lazdunski M, Heurteaux C. MLC901, a Traditional Chinese Medicine induces neuroprotective and neuroregenerative benefits after traumatic brain injury in rats. Neuroscience. 2014 Sep 26; 277:72-86. doi: 10.1016/j.neuroscience.2014.06.047. Erratum in: Neuroscience. 2014 Oct 10; 278:265-6.

38. Tsai MC, Chang CP, Peng SW, Jhuang KS, Fang YH, Lin MT, Tsao TC. Therapeutic efficacy of Neuro AiD™ (MLC 601), a traditional Chinese medicine, in experimental traumatic brain injury. J Neuroimmune Pharmacol. 2015 Mar;10(1):45-54. doi: 10.1007/s11481-014-9570-0.

39. Bavarsad Shahripour R, Hemati A, Hosseinzadeh Maleki A. A randomized trial to assess the long-term safety of NeuroAiD among Caucasian patients with acute ischemic stroke. Chin J Integr Med. 2014;20(11):812-7.

40. Navarro JC, Molina MC, Baroque Ii AC, Lokin JK. The Use of NeuroAiD (MLC601) in Postischemic Stroke Patients. Rehabil Res Pract. 2012; 2012:506387. doi: 10.1155/2012/506387.

41. Ghandehari K, Izadi Mood Z, Ebrahimzadeh S, Picard D, Zhang Y. NeuroAid (MLC601) versus piracetam in the recovery of post-infarct homonymous hemianopsia. Neural Regen Res. 2011;6(6):418-22. doi: 10.3969/j.issn.1673-5374.2011.06.003.

42. Venketasubramanian N, Chen CL, Gan RN, Chan BP, Chang HM, Tan SB, Picard D, Navarro JC, Baroque AC 2nd, Poungvarin N, Donnan GA, Bousser MG; CHIMES Investigators. A double-blind, placebo-controlled, randomized, multicenter study to investigate CHInese Medicine Neuroaid Efficacy on Stroke recovery (CHIMES Study). Int J Stroke. 2009 Feb;4(1):54-60. doi: 10.1111/j.1747-4949.2009.00237.

43. Chen CL, Young SH, Gan HH, Singh R, Lao AY, Baroque AC 2nd, Chang HM, Hiyadan JH, Chua CL, Advincula JM, Muengtaweepongsa S, Chan BP, de Silva HA, Towanabut S, Suwanwela NC, Poungvarin N, Chankrachang S, Wong KS, Eow GB, Navarro JC, Venketasubramanian N, Lee CF, Bousser MG; CHIMES Study Investigators. Chinese medicine neuroaid efficacy on stroke recovery: a double-blind, placebo-controlled, randomized study. Stroke. 2013 Aug;44(8):2093-100. doi: 10.1161/STROKEAHA.113.002055.

44. Venketasubramanian N, Young S, Tay SS, Chang HM, Umapathi T, Chan B, de Silva A, Wong L, Navarro J, Zhao YD, Tan SB, Chen C. Chinese medicine NeuroAiD efficacy stroke recovery-extension study (CHIMES-E study): an observational multicenter study to investigate the longer-term efficacy of NeuroAiD in stroke recovery. Cerebrovasc Dis. 2013;35 Suppl 1:18-22. doi: 10.1159/000346233.

45. Venketasubramanian N, Young SH, Tay SS, Umapathi T, Lao AY, Gan HH, Baroque AC 2nd, Navarro JC, Chang HM, Advincula JM, Muengtaweepongsa S, Chan BP, Chua CL, Wijekoon N, de Silva HA, Hiyadan JH, Suwanwela NC, Wong KS, Poungvarin N, Eow GB, Lee CF, Chen CL; CHIMES-E Study Investigators. CHInese Medicine NeuroAiD Efficacy on Stroke Recovery - Extension Study (CHIMES-E): A Multicenter Study of Long-Term Efficacy. Cerebrovasc Dis. 2015;39(5-6):309-18. doi: 10.1159/000382082.

46. Gualtieri C.T., Johnson L.G. “Reliability and validity of a computerized neurocognitive test battery, CNS Vital Signs”. Archives of Clinical Neuropsychology, 22, 623-43 (2006)

47. Johnson L.G., Gualtieri C.T., “A computerized test battery sensitive to mild and severe brain injury. Medscape J Med, 10, 90 (2008)

48. Gualtieri CT, Johnson LG, Benedict KB. Psychometric and clinical properties of a new, computerized neurocognitive assessment battery. Bal Harbor, FL: American Neuropsychiatric Association Annual Meeting, 2004

49. Kaishibayev S. et al. Efficacy of NeuroAiD (MLC901) in combination therapy during early rehabilitation period of patients who had suffered an ischemic stroke (Study report).

50. Murie-Fernández M, Marzo MM. Predictors of Neurological and Functional Recovery in Patients with Moderate to Severe Ischemic Stroke: The EPICA Study. Stroke Res Treat. 2020;2020:1419720. Published 2020 May 1. doi:10.1155/2020/1419720.

17 APPENDICES

17.1 Glasgow Coma Scale

To obtain the Glasgow Coma Scale score it is necessary to sum up the scores for each of the 3 parameters (eye opening, best motor response, verbal response)

Parameter	Score
**Eye opening**	
Spontaneous – open before stimulus	4
To sound - after spoken or shouted request	3
To pressure - after finger tip stimulus	2
No opening at any time, no interfering factor	1
Closed by local factor	Non testable
**Best motor response**	
Obeys commands - obeys 2-part request	6
Brings hand above clavicle to stimulus on head neck - localising	5
Bends arm at elbow rapidly but features not predominantly abnormal – normal flexion	4
Bends arm at elbow, features clearly predominantly abnormal – abnormal flexion	3
Extends arm at elbow	2
No movement in arms / legs, no interfering factor	1
Paralysed or other limiting factor	Non testable
**Verbal response**	
Correctly gives name, place and date - orientated	5
Not oriented but communication coherently - confused	4
Intelligible single words	3
Only moans / groans	2
No audible response, no interfering factor	1
Factors interfering with communication	Non testable
Source: www.glasgowcomascale.org	

18 AMENDMENTS TO THE PROTOCOL

Amendment No. 1: Previous Protocol version 1.0 (18.01.2021), corrected to Protocol version 2.0

Justification for the amendment: Request of Ministry of Health of Russian Federation

**Previous text (version 1.0):**	**Changed to (version 2.0):**	**Justification for the amendment:**
**Study synopsis,**<br>**3.Study aims, objectives, and endpoints,**<br>**7.2.Methods and Timing for Assessing, Recording, and Analyzing of Efficacy Parameters**<br>**9.1.2. Efficacy analysis**<br>Secondary efficacy endpoints 3,4,5	**Study synopsis,**<br>**3.Study aims, objectives, and endpoints,**<br>**7.2.Methods and Timing for Assessing, Recording, and Analyzing of Efficacy Parameters**<br>**9.1.2. Efficacy analysis**<br>Secondary efficacy endpoints 3,4,5 moved to Exploratory endpoints	MoH request concerning smaller number of secondary endpoints
**Study synopsis,**<br>**3.Study aims, objectives, and endpoints,**<br>**9.1.2. Efficacy analysis**<br>*Wording for efficacy endpoints changed*<br>**Primary efficacy endpoint**<br>Complex attention improvement, assessed by change from baseline in the values, determined by Central Nervous System Vital Signs (CNS-VS) system in adult patients with long-term cognitive impairment following mTBI, randomized to receive NeuroAiD II™ (MLC901) after 6 months of treatment compared to patients with long-term cognitive impairment following mTBI, randomized to placebo.<br>**Secondary efficacy endpoints**<br>• Improvement in the following cognitive domains: executive functioning, processing speed, memory (visual and verbal) and reaction time, assessed by change from baseline in values, determined by CNS-VS system in adult patients with long-term cognitive impairment following mTBI, randomized to receive NeuroAiD II™ (MLC901), after 6 months of treatment compared to patients with long-term cognitive impairment following mTBI, randomized to placebo.<br>• Improvement of post-concussion symptoms, assessed by change from baseline in values, determined by Rivermead Post Concussion Symptoms Questionnaire (RPQ) in adult patients with long-term cognitive impairment following mTBI, randomized to receive	**Study synopsis,**<br>**3.Study aims, objectives, and endpoints,**<br>**9.1.2. Efficacy analysis**<br>*Wording for efficacy endpoints changed*<br><u>Primary efficacy endpoint:</u> Change in complex attention score, determined using Central Nervous System Vital Signs (CNS-VS) computer cognitive testing system, after 6 months of treatment compared to baseline in the group of patients receiving NeuroAiD II™ (MLC901), compared to the placebo group.<br><u>Secondary efficacy endpoints</u><br>- Changes in the scores for the following cognitive domains: executive functioning, processing speed, memory (visual and verbal) and reaction time, determined by CNS-VS system, after 6 months of treatment compared to baseline, in the NeuroAiD II™ (MLC901) group, compared to the placebo group.<br>- Change in the Rivermead Post Concussion Symptoms Questionnaire (RPQ) total score, after 6 months of treatment, compared to baseline, in the NeuroAiD II™ (MLC901) group compared to the placebo group.<br><u>Exploratory efficacy endpoints</u><br>• Change in the Health-related quality of life questionnaire (QOLIBRI) total score after 6 months of treatment, compared to baseline, the NeuroAiD II™ (MLC901) group compared to the placebo group.<br>• Change in the Hospital Anxiety and Depression Scale (HADS) score after 6 months of	MoH request concerning wording of efficacy endpoints

NeuroAiD II™ (MLC901) after 6 months of treatment compared to patients randomized to placebo.<br>• Improvement in the quality of life, assessed by change from baseline in values, determined by Health-related quality of life questionnaire (QOLIBRI) in adult patients with long-term cognitive impairment following mTBI, randomized to receive NeuroAiD II™ (MLC901) after 6 months of treatment compared to patients randomized to placebo.<br>• Improvement in the levels of anxiety and depression, assessed by change from baseline in values, determined using Hospital Anxiety and Depression Scale (HADS) in adult patients with long-term cognitive impairment following mTBI, randomized to receive NeuroAiD II™ (MLC901) after 6 months of treatment compared to patients randomized to placebo.<br>• Improvement, compared to baseline, in the analyzed parameters (complex attention, executive functioning, processing speed, memory (visual and verbal), reaction time, post-concussion symptoms, quality of life, the levels of anxiety and depression) in adult patients with long-term cognitive impairment following mTBI after 9 months of NeuroAiD II™ (MLC901) treatment initiation (i.e. 3 months after NeuroAiD II™ (MLC901) treatment completion) with reference to the same placebo-treated group of adult patients with long-term cognitive impairment following mTBI. This will be performed for the parameters which will be improved (if any) after 6 months of NeuroAiD II™ (MLC901) treatment in the same population of patients.	treatment, compared to baseline, in the NeuroAiD II™ (MLC901) group compared to the placebo group.<br>• Changes, compared to baseline, in the analyzed parameters (complex attention, executive functioning, processing speed, visual and verbal memory, reaction time, RPQ score, QOLIBRI score, HADS score) 9 months after NeuroAiD II™ (MLC901) treatment initiation with reference to baseline, compared to the placebo group. .	
**Synopsis,**<br>**5.Selection and withdrawal of patients**<br>*Inclusion criterion 1 – Wording changed*<br>…Mild TBI is evidenced by any of the following:<br>• worst Glasgow Coma Score 13-15 as assessed on scene, on admission and over next 3 days<br>• loss of consciousness for 0-30 minutes	**Synopsis,**<br>**5.Selection and withdrawal of patients**<br>*Inclusion criterion 1 – Wording changed*<br>…The mTBI diagnosis should be documented. Mild TBI is evidenced by any of the following:<br>• best Glasgow Coma Score 13-15 as assessed on scene, on admission and over next 3 days<br>• loss of consciousness for up to 30 minutes<br>• dazed and confused at the time of injury or post-TBI amnesia of < 24 hours duration.	To provide a more correct definition of mTBI

• evidence of being dazed and confused or not remembering what happened immediately before or after the accident<br><br>These manifestations of TBI must not be due to drugs, alcohol or medications, caused by other injuries or treatment for other injuries (e.g., systemic injuries, facial injuries or intubation), or caused by other problems (e.g., psychological trauma, language barrier or co-existing medical conditions). However, other physical injuries sustained at the time of TBI are not exclusion criteria.		
**Synopsis,**<br><br>**5. Selection and withdrawal of patients**<br><br>*Exclusion criterion 1 – Wording changed*<br><br>Moderate or Severe TBI, determined by Glasgow Coma Score of <13, fatal injury or requiring neurosurgery (even if surgery was not done)<br><br>*Exclusion criterion 6 – changed*<br><br>Unknown date of injury.	**Synopsis,**<br><br>**5. Selection and withdrawal of patients**<br><br>*Exclusion criterion 1 – Wording changed*<br><br>Moderate or Severe TBI, determined by best Glasgow Coma Score of <13 (as assessed on scene, on admission and over next 3 days), fatal or injury or requiring neurosurgery (even if surgery was not done)<br><br>*Exclusion criterion 6 – changed*<br><br>No documented evidence of mTBI	To provide a more correct definition of mTBI
**Synopsis,**<br><br>**Information about restricted medication was added**<br><br>-	**Synopsis,**<br><br>**Information about restricted medication was added**<br><br>The medications to be restricted during the study include drugs with nootropic, metabolic or sedative effects, or those which can exert such effects based on the Investigator's opinion or otherwise can strongly affect patient's performance upon CNS-VS testing. The Investigator should prescribe any of these drugs only upon consultation with the Sponsor and after the Sponsor approves the use of such medication.	MoH request concerning concurrent medication
**Synopsis,**<br><br>**Sections 9.1.1., 9.1.2.**<br><br>*following text removed:*<br><br>Data collected at baseline will be reported and compared between the NeuroAiD II™ and Control (placebo) groups. These baseline differences and differences in outcome will be tabulated between the NeuroAiD II™ and the Control group at 1, 3, 6 and 9-month follow-up. These will be summarized using means (95% confidence intervals [CI]), standard deviations, medians, quartiles, minimums and maximums.	**Synopsis**<br><br>**Sections 9.1.1., 9.1.2.**<br><br>*Following text added:*<br><br>Data on demographics, laboratory results, vital signs, physical examination will be presented using the following descriptive statistics: number of observations, percentages, mean, standard deviation, median, quartiles, minimum and maximum values.<br><br>These baseline differences and differences in outcomes at 1, 3, 6 and 9-month follow-up will be summarized using means, standard deviations, medians, quartiles, minimums and maximums.<br><br>…	MoH request about detailing of statistical methods

…<br><br>The hierarchical principle will be used to address multiplicity while testing statistical hypotheses. Confirmatory analysis of secondary endpoints will be considered if the superiority is claimed by primary endpoint. A multivariate mixed repeated measures analysis of covariance model will be used, adjusting for baseline and potential covariates including but not limited to age and time since injury. The participant and site will be used to represent the random effects. The random-effects approach here will account for any imbalance in sample sizes recruited per site, or proportions allocated per treatment group. Model selection will be undertaken with each outcome using standard selection heuristics. Covariates will be selected based on improving the overall efficiency of the model. Regardless, baseline and age, gender, time since injury (1-3 months/4-12 months) and study center will be included as covariates in the multivariate mixed repeated measures analysis of covariance model.	For primary endpoint analysis for each patient, the difference in complex attention scores, determined using CNS-VS computer testing, after 6 months of therapy compared to baseline, will be calculated: the change in complex attention score compared to baseline. Mixed effects model (PROC MIXED) will be used with adjustments for baseline and potential covariates. All timepoints will be used for the model. The participant and site will be used as the random effects. Model selection will be undertaken with each outcome using standard selection heuristics. Covariates will be selected based on improving the overall efficiency of the model. Regardless, baseline and age, gender, time since injury (1-3 months/4-12 months) and study center will be included as covariates in the mixed effects model. Descriptive statistics will be calculated to change in complex attention in each group, as well as mean values obtained by the method of least squares from the mixed effects model.<br><br>Superiority of MLC901 over placebo will be claimed if<br><br>H0: $LSM_T >= LSM_R$ is rejected and thereafter<br><br>H1: $LSM_T < LSM_R$ is accepted at significance level of 5%,<br><br>where LSM_T is a Least Square Mean of Change from Baseline of Complex attention score after 6 months of treatment assessed by the mixed model repeated measures analysis in the MLC901 group and LSM_R is a Least Square Mean of Change from Baseline of Complex attention score after 6 months of treatment assessed by the mixed model repeated measures analysis in the Placebo group (lower score of Complex attention is better).<br><br>…<br><br>The same approach as for primary endpoint will be used for analysis of secondary efficacy endpoints and exploratory endpoints, and completely similar hypotheses are be formulated as for the analysis of the primary endpoint. Mixed effects models will be used to analyze the change in values from baseline, adjusted for covariates and baseline estimates. No sensitivity analysis will be performed for secondary endpoints. No adjustments will be made for multiple comparisons, hypotheses will be tested hierarchically, hypothesis testing by secondary endpoints will be taken into account if statistically significant differences between groups for the primary endpoint are confirmed.	
**Synopsis**<br><br>**Sample Size Justification** – *Wording changed*<br><br>An estimate of 20 was obtained from the BRAINS pilot study (21) for standard	**Synopsis**<br><br>**Sample Size Justification** – *Wording changed*<br><br>Sample size was calculated using PASS 14 software.	MoH request concerning sample size justification

deviation of change in complex attention from baseline to 6 months, 128 subjects are required to detect a clinically meaningful difference of 10 in the mean changes in complex attention between MLC901 and placebo arms with 80% power at a two-sided 5% significance level. The sample size is increased to 182 subjects (91 per arm) to allow for 30% dropouts. Considering very high screening failure rate up to 364 subjects can be screened.	Assumptions for sample size calculations:<br><br>An estimate of 20 for standard deviation of change in complex attention from baseline to 6 months was obtained from the BRAINS pilot study of NeuroAid II (MLC901) in 78 patients with mild or moderate TBI. A minimally clinically meaningful difference of -10 in the mean change in complex attention, determined by CNS-VS, per group was determined. Target power - 80%. Two-sided significance level - 5%. The proportion of subjects in groups – 1:1. Hypotheses: H_0: $M_T \geq M_R$, H1: $M_T < M_R$, where M_T is an arithmetical mean of change of Complex attention score after 6 months of treatment, compared to baseline, in the MLC901 group, and M_R is an arithmetical mean of change from baseline of Complex attention score after 6 months of treatment in the placebo group (lower score of Complex attention is better).<br><br>128 subjects are required to detect a clinically meaningful difference of -10 in the mean changes in complex attention between MLC901 and placebo arms with 80% power at a two-sided 5% significance level. The sample size is increased to 182 subjects (91 per arm) to allow for 30% dropouts. Considering very high screening failure rate up to 364 subjects can be screened.	
**2.3.1.Nonclinical studies**<br><br>-	**Nonclinical studies**<br><br>*Information on 2 preclinical studies of IP in mTBI in rats added in the end of section*	MoH request concerning IP preclinical studies in mTBI
**2.3.2.Clinical studies**<br><br>-	**2.3.2.Clinical studies**<br><br>*Detailed safety information from prior clinical studies of MLC601 and MLC901 was added, Table 2.3 added*<br><br>*Detailed information about previous clinical studies of MLC901 added*	MoH request concerning IP safety data and clinical trials of MLC901
**2.5.Description of and Justification for the Route of Administration, Dosage, Dosage Regimen, and Treatment Period**<br><br>-	**2.5.Description of and Justification for the Route of Administration, Dosage, Dosage Regimen, and Treatment Period**<br><br>Detailed dose rationale and information about overall extent of exposure in clinical trials was added,<br><br>Table 2.4 added	MoH request concerning dose justification
**5.Selection and withdrawal of patients**<br><br>Mild TBI is defined as an external force from an incident causing injury to the brain and resulting in an altered level of consciousness, evidenced by *worst Glasgow Coma Score of 13-15 as assessed on scene, on admission and over next 3 days* **or** *loss of consciousness for 0-30 minutes* **or** *evidence of being dazed and confused or*	**5.Selection and withdrawal of patients**<br><br>- (*text about mTBI recall in patients removed*)	MoH request concerning documented mTBI

not remembering what happened immediately before or after the accident. In the absence of medical records documenting TBI, the questions potential participants should be asked to document TBI occurrence are:<br>• Did you lose consciousness (knocked out)?<br>• Were you dazed, confused, or seeing stars?<br>• Did you have difficulty remembering the injury (what happened)?		
**6.2.Medications/Treatments Permitted (Including Rescue Medication) and Not Permitted Before and/or During the Study**<br>All participants will continue to receive standard medical care, without any changes in medical treatment recorded. No medications or procedures will be prohibited.	**6.2.Medications/Treatments Permitted (Including Rescue Medication) and Not Permitted Before and/or During the Study**<br>All participants will continue to receive standard medical care, without any changes in medical treatment recorded except for the medications which should be restricted during the study. The medications to be restricted in this study include drugs that can potentially affect the study results, e.g., most importantly, patients' performance upon CNS-VS testing.<br>These medications to be restricted during the study include drugs with nootropic, metabolic or sedative effects, or those which can exert such effects based on the Investigator's opinion. The Investigator should prescribe any of these drugs only upon consultation with the Sponsor and after the Sponsor approves the use of such medication.	MoH request concerning concurrent medication
**7.2.Methods and Timing for Assessing, Recording, and Analyzing of Efficacy Parameters**<br>-	**7.2.Methods and Timing for Assessing, Recording, and Analyzing of Efficacy Parameters**<br>*Information about CNS-VS added:*<br>CNS-VS is a reliable and validated instrument of TBI studies, and notably for studies including mild TBI patients.<br>Details about this testing system are available online at https://www.cnsvs.com.<br>During the CNS-VS testing, a patient undergoes 7 cognitive tests, after which, based on the results of these tests, the system automatically calculates scores for a number of cognitive domains: complex attention, neurocognitive index, executive functioning, memory (visual and verbal), reaction time, psychomotor speed, processing speed, simple attention, motor speed, composite memory, cognitive flexibility.<br>Its psychometric properties have been extensively studied, including test-retest reliability, sensitivity, concurrent validity with other psychometric tests and discriminant validity in different clinical settings (various levels of TBI severity, mild	MoH request concerning details of primary endpoints

	cognitive impairment, depression, ADHD, etc) (46,47,48). In a study of Gualtieri, CNS-VS complex attention score was compared between patients who had recovered from mild brain injury/concussion (with loss of consciousness less than 20 minutes and/or transient post-traumatic amnesia (less than 24 hours) and normal patients (46-48). CNS-VS Complex Attention score was assessed at 100.41 in the subgroup on normal patients, as compared to an average of 92.8 in the mild brain injury/concussion subgroup (46-48). These results suggest that a difference in complex attention score in the range of 8-10 is clinically important and distinguish normal subjects from subjects with some level of post brain injury impairment.	
**Table 7.1**<br><br>Complex attention improvement in adult patients with long-term cognitive impairment following mTBI, who were randomized to receive NeuroAiD II™ (MLC901) compared to patients randomized to placebo.<br><br>Improvement in the following cognitive domains: executive functioning, processing speed, memory (visual and verbal) and reaction time, in adult patients with long-term cognitive impairment following mTBI, who were randomized to receive NeuroAiD II™ (MLC901), compared to patients randomized to placebo.<br><br>Improvement of post-concussion symptoms in adult patients with long-term cognitive impairment following mTBI, who were randomized to receive NeuroAiD II™ (MLC901) compared to patients randomized to placebo.<br><br>Improvement in the quality of life, in adult patients with long-term cognitive impairment following mTBI, who were randomized to receive NeuroAiD II™ (MLC901) compared to patients randomized to placebo.<br><br>Improvement in the levels of anxiety and depression, in adult patients with long-term cognitive impairment following mTBI, who were randomized to receive NeuroAiD II™ (MLC901) compared to patients randomized to placebo.<br><br>Improvement, compared to baseline, in the analysed parameters (complex attention, executive functioning, processing speed, memory (visual and verbal), reaction time, post-concussion symptoms, quality of life,	**Table 7.1**<br><br>Change in Complex attention score in adult patients with long-term cognitive impairment following mTBI, who were randomized to receive NeuroAiD II™ (MLC901) compared to patients randomized to placebo.<br><br>Changes in the scores for the following cognitive domains: executive functioning, processing speed, memory (visual and verbal) and reaction time, in adult patients with long-term cognitive impairment following mTBI, who were randomized to receive NeuroAiD II™ (MLC901), compared to patients randomized to placebo.<br><br>Change in the total score for post-concussion symptoms in adult patients with long-term cognitive impairment following mTBI, who were randomized to receive NeuroAiD II™ (MLC901) compared to patients randomized to placebo.<br><br>Change in the total score for the quality of life, in adult patients with long-term cognitive impairment following mTBI, who were randomized to receive NeuroAiD II™ (MLC901) compared to patients randomized to placebo.<br><br>Change in the total score for levels of anxiety and depression, in adult patients with long-term cognitive impairment following mTBI, who were randomized to receive NeuroAiD II™ (MLC901) compared to patients randomized to placebo.<br><br>Changes, compared to baseline, in the analysed parameters (complex attention, executive functioning, processing speed, memory (visual and verbal), reaction time, post-concussion symptoms, quality of life, the levels of anxiety and depression) in adult patients with long-term cognitive impairment following mTBI with reference to the placebo group	Wording changed for efficacy parameters to make them clearer

the levels of anxiety and depression) in adult patients with long-term cognitive impairment following mTBI with reference to the same placebo-treated group of adult patients with long-term cognitive impairment following mTBI. This will be performed for the parameters which will be improved (if any) after 6 months of NeuroAiD II™ (MLC901) treatment in the same population of patients.		
**9.1.1.Descriptive analysis of demography and baseline data**<br>Baseline value for all parameters is defined as the most recent estimate before randomization.	**9.1.1.Descriptive analysis of demography and baseline data**<br>**(added to Synopsis)**<br>Baseline value for all parameters is defined as the most recent estimate before first drug intake.	To avoid mistakes in Study procedures
**9.1.2. Efficacy analysis**<br>Exploratory descriptive and visual analysis will be employed for differences in the shape of recovery curves for the analysed parameters (complex attention, executive functioning, processing speed, memory (visual and verbal), reaction time, post-concussion symptoms, quality of life, levels of anxiety and depression) of the group randomized to receive NeuroAiD II™ (MLC901) and the placebo group with time points 0, 1, 3, 6 and 9 months after treatment initiation.	**9.1.2. Efficacy analysis**<br>*Removed*	To make description of statistical analysis clearer according to the request of MoH
**9.1.3. Safety analysis**	**9.1.3. Safety analysis**<br>Adverse events will be coded using MedDRA dictionary.	Details of safety analysis added

<table>
<tr>
<td>9.2.Number of Subjects Planned to be Enrolled

An estimate of 20 was obtained from the BRAINS pilot study (21) for standard deviation of change in complex attention from baseline to 6 months, 128 subjects are required to detect a clinically meaningful difference of 10 in the mean changes in complex attention between MLC901 and placebo arms with 80% power at a two-sided 5% significance level. The sample size is increased to 182 subjects (91 per arm) to allow for 30% dropouts. Considering very high screening failure rate up to 364 subjects can be screened.</td>
<td>9.2.Number of Subjects Planned to be Enrolled

Following text and Table 9.1 added:

The primary endpoint for the trial is change in complex attention score, determined using CNS-VS computer cognitive testing system, after 6 months of treatment compared to baseline in the group of patients receiving NeuroAiD II™ (MLC901), compared to the placebo group. Primary Analysis: the primary analysis will compare the mean change in the complex attention score, determined by CNS-VS testing, after 6 months of therapy after 6 months of treatment, compared to baseline, between the MLC901 group and the placebo group by analysis of variance in the ITT population.

In a study of Gualtieri (46-48), CNS-VS complex attention score was compared between patients who had recovered from mild brain injury/concussion (with loss of consciousness less than 20 minutes and/or transient post-traumatic amnesia (less than 24 hours) and normal patients. CNS-VS Complex Attention score was assessed at 100.41 in the subgroup on normal patients, as compared to an average of 92.8 in the mild brain injury/concussion subgroup (46,47,48). These results suggest that a difference in complex attention score in the range of 8-10 is clinically important and distinguish normal subjects from subjects with some level of post brain injury impairment.

The sample size for this study is estimated based upon a previous study in 78 patients evaluating the effect of MLC-901 (NeuroAiD II) versus placebo using CNS-VS as main efficacy assessment (21).

…

While the scores are very similar at baseline, there is a difference of about 12 points at 6 months between placebo and MLC-901 groups, in favor of the active treatment. Based upon these results, sample size for current study has been calculated in order to provide 80% power to detect a difference of 10 points in CNS-VS Complex Attention score change from baseline after 6 months (at a 2-sided alpha level 5%). The standard deviation used in this calculation for the parameter is estimated to be 20.

Study is powered to claim superiority of MLC901 over Placebo by primary endpoint for following statistical hypothesis: H0: $LSM_T >= LSM_R$, H1: $LSM_T < LSM_R$, where LSM_T is a Least Square Mean of Change from Baseline of Complex attention score after 6 months of treatment assessed by the mixed model repeated measures analysis in the MLC901 group and LSM_R is a Least Square Mean of Change from Baseline of Complex attention score after 6 months of treatment assessed by the mixed model repeated measures analysis in</td>
<td>MoH request concerning sample size justification</td>
</tr>
</table>

	the Placebo group (lower score of Complex attention is better).<br><br>PASS 14 software (Scenario: two sample T-test assuming equal variance) was used to estimate the required sample size. Target power – 80%. Alpha – 0.05. Proportion of subjects in groups – 1:1.<br><br>Hypotheses: H0: MT ≥MR, H1: MT < MR, where MT is an arithmetical mean of change of Complex attention score after 6 months of treatment, compared to baseline, in the MLC901 group, and MR is an arithmetical mean of change from baseline of Complex attention score after 6 months of treatment in the placebo group (lower score of Complex attention is better).<br><br>An estimate of 20 was obtained from the BRAINS pilot study (21) for standard deviation of change in complex attention from baseline to 6 months, 128 subjects (64 per arm) are required to detect a clinically meaningful difference of -10 in the mean changes in complex attention between MLC901 and placebo arms with 80% power at a two-sided 5% significance level. Sensitivity analysis: 1) power is equal 29% if the mean difference of primary estimate is 5 and power is equal 99% if the mean difference of primary estimate is 20; 2) power is equal 15% if the standard deviation increases up to 30, so some uncertainty in the standard deviation estimate may cause insufficient actual power for comparison.<br><br>The sample size is increased to 182 randomized subjects (91 per arm) to allow for 30% dropouts. Considering very high screening failure rate up to 364 subjects can be screened.	
**11.5.Steering Committee**<br><br>The committee provides advice to the Principal Investigator (PI).<br><br>• Taking steps to reduce and minimize deviations from the study protocol, recommend protocol amendments if needed;<br><br>• To resolve any discrepancies within the Study team, or between Study team and the Sponsor (Moleac) regarding the study's data management and monitoring procedures or any recommendations for modifications to the Study Protocol;	**11.5.Steering Committee**<br><br>*Information about SC added:*<br><br>-<br><br>• Monitor recruitment rates and take steps to reduce and minimize deviations from the study protocol, recommend protocol amendments if needed;<br><br>• Amicably resolve any issues within the research team and the Sponsor regarding the study's data management and monitoring procedures or any recommendations for modifications to the Study Protocol;	To provide more correct information on SC functions

The SC will consist of at least 5 members, one of whom is a Medical Affairs personnel, and one as a permanent guest of the Sponsor (Moleac).	The SC will consist of at least 5 members, including Chair of the SC	
**11.6.Data Safety Monitoring Board (DSMB)**<br>-<br>…<br>-<br>The DSMB will consist of at least three members, with at least one biostatistician. DSMB membership will consist of persons with strong scientific expertise and track record in the field of Traumatic Brain Injury and no conflict of interest.	**11.6.Data Safety Monitoring Board (DSMB)**<br>• To review the DSMB charters and make recommendation for any changes<br>…<br>• To protect the confidentiality of the study data and the DSMB discussions.<br>The DSMB will consist of at least three members, with at least one biostatistician, an expert in the field of TBI, and a medical expert with no conflict of interest.	To provide more correct information on DSMB functions
**15.PUBLICATION POLICY**<br>Publication of study materials without the written permission of the Sponsor is not permitted.<br>The Sponsor may publish any data and information related to the study (including data and information obtained by the Investigator) without the consent of the Investigator.	**15.PUBLICATION POLICY**<br>The PI or investigator shall be free to make scholarly publications on the findings of the project upon approval of the Steering Committee and the Sponsor. To ensure compliance with confidentiality, the Sponsor will provide the other with a copy of any proposed disclosures related to the project in advance of the proposed disclosure. There will be no constraints applied to examination of thesis and other publications. If necessary, publication of a thesis may be subject to approval and agreed timeline by the Sponsor.	To provide the Investigator the possibility to publish scientific data
**16.REFERENCES**	**16.REFERENCES**<br>Publications 37-50 added	To provide references for added information of preclinical and clinical studies
	**Table of contents, table of abbreviations, misprints corrected**	

Amendment No. 2: Previous Protocol version 2.0 (08.04.2021), corrected to Protocol version 3.0

Justification for the amendment: Protocol clarification required per Quality Assurance.

Previous text (version 2.0):	Changed to (version 3.0):	Justification for the amendment:
**1.3 Study Schedule**<br>**Table .1.1 Schedule of study procedures**<br>Added text in the footnote of the table (as in next column)	**1.3 Study Schedule**<br>**Table .1.1 Schedule of study procedures**<br>** Not necessary if screening (visit 1) and randomization (visit 2) are performed on the same day (which will be Day 1).	To provide clarification
**4.2.1. Randomization**<br>This approach will help to stratify by study center, time since injury [1-3 months/4-12 months] and gender.	This approach will help to stratify by study center, time since injury [1-3 months/4-12 months] and gender. Time since injury between 3 and 4 months will be stratified to 1-3 months.	To provide clarification
