# Supplementary material for: A double-blind, placebo-controlled, randomized, multi-centre, phase III study of MLC901 (NeuroAiDTMII) for the treatment of cognitive impairment after mild traumatic brain injury
Source: PLoS One. 2025 Jul 10;20(7):e0310229. doi: 10.1371/journal.pone.0310229 (PMC12244563; doi:10.1371/journal.pone.0310229)
Supplement: S2 File — (PDF) [file pone.0310229.s002.pdf]

SUPPLEMENTARY materials

Table of content

FIGURE S1. STUDY PROCESSES 2

ADDITIONAL DETAILS ABOUT POWER CALCULATION 3

TABLE S2. CNS VITAL SIGNS TEST DESCRIPTIONS AND COMPUTATION OF DOMAIN SCORES 4

TABLE S3. MEANS AND STANDARD DEVIATIONS FOR THE OUTCOME MEASURES ACROSS TIME-POINTS........ 7

TABLE S4. RIVERMEAD POST-CONCUSSION QUESTIONNAIRE SCORES IN THE MLC901 AND PLACEBO GROUPS AT BASELINE, 3, 6 AND 9 MONTHS POST-RANDOMISATION 8

REFERENCES 10

Figure S1. Study processes

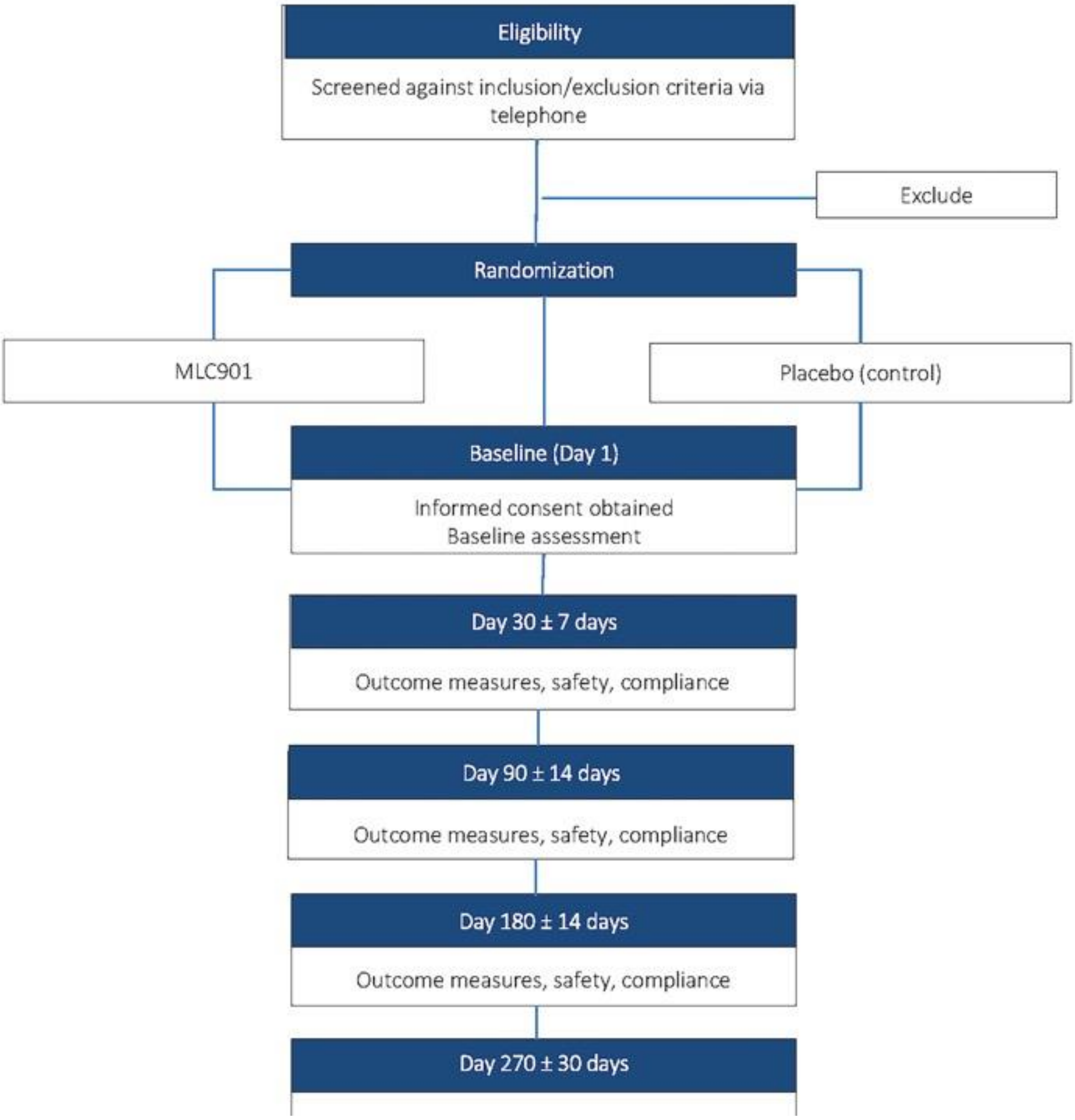

Additional details about power calculation

The sample size calculation for the trial was based upon the results obtained in the pilot study done in New Zealand by Theadom et al (Table 1).[1]

Table S1. Complex Attention score : Mean (SD)

	MLC-901 (n=36)	Placebo (n=42)
**Baseline**	83.86 (22.06)	85.43 (16.52)
**Month 6**	94.18 (15.29)	82.31 (21.56)

As can be seen in the table above, the SD for the score at M6 is in the range 15-21, so the SD for the change from baseline at M6 is likely to be < 20.

However, in view of some uncertainty about the variability of the measure, and in order to be conservative and achieve sufficient power for the trial, we designed the study protocol using a SD of 20.

The intraclass correlation for centers was not taken into account in the power calculation, as there was limited information to use as to which magnitude of correlation to consider in the calculation. In the final analysis, however, the intraclass correlation for centers is accounted for, via including study centers as fixed effect in the model.

It can be noted also that the withdrawal rate of 30% planned for in the power calculation turned out to be largely overestimated, resulting in a power for the trial higher than the originally planned 80%.

Table S2. CNS Vital Signs test descriptions and computation of domain scores

Cognitive Domain	Tests	Test Description	Scoring
Verbal Memory	Verbal Memory Test (VMT)	The VMT measures recognition and memory for words. There are two parts to the test. For immediate recognition, fifteen words are presented on the screen one by one, every two seconds and the participant has to identify those words nested among a set of new words. For the delayed recognition trial, after conducting six other tests, participants are asked again to identify those words nested among new words.	VMT Correct Hits Immediate + VMT Correct Passes Immediate + VMT correct Hits Delay + VMT Correct Passes Delay
Visual Memory	Visual Memory Test (VIM)	The VIM measures recognition and memory for figures. Fifteen geometric figures are presented on the screen, one by one. For the immediate recognition test, the participant has to identify the figures nested among a set of new figures. Then, after five more tests, there is a delayed recognition trial.	VIM Correct Hits Immediate + VIM Correct Passes Immediate + VIM Correct Hits Delay + VIM Correct Passes Delay
Psychomotor Speed	Finger Tapping Test (FTT) Symbol Digit Coding Test (SDC)	The FTT test requires participants to press the space bar with their right index finger as many times as they can in 10 seconds. There is one practice test, and then there are three test trials. The test is repeated with the left hand.	FTT Right Taps Average + FTT Left Taps Average + SDC Correct Responses
Processing Speed	Symbol Digit Coding Test (SDC)	For the SDC test eight symbols are presented on the screen with a number below each symbol. The participant types in the number that	SDC Correct Responses - SDC Errors

		corresponds to the symbol that is highlighted. Only the digits from 2 through 9 are used to avoid any potential confusion between “1” and “l” on the keyboard. The numerical pad is not able to be used, to reduce bias for those skilled at using the numerical pad or for those that are right- versus left-handed.	
Reaction Time	Stroop Test (ST)	The Stroop test has three parts. In the first part, the words red, yellow, blue, and green (printed in black) appear at random on the screen, and the participant presses the space bar as soon as they see the word. In the second part, the words red, yellow, blue, and green appear on the screen, printed in colour. The participant presses the space bar when the color ofthe word matches what the word says. In the third part, the words red, yellow, blue, and green appear on the screen, printed in colour. Participants press the space bar when the colour of the word does not match what the word says.	(ST Complex Reaction Time Correct + Stroop Reaction Time Correct) / 2
Complex attention	Stroop Test (ST)		Stroop Commission Errors + SAT Errors + CPT Commission Errors + CPT Omission Errors
Cognitive Flexibility	Shifting Attention Test (SAT)<br>Stroop Test (ST)	The SAT test measures the ability to shift from one instruction set to another quickly and accurately. Three figures appear on the screen, one on top and two on the bottom. The top figure is either a square or a circle. The bottom figures are a square and a circle. The figures are either red or blue (mixed randomly). The participant is asked to match one of the bottom figures to the top figure with the	SAT Correct Responses - SAT Errors - Stroop Commission Errors
Executive Functioning	Shifting Attention Test (SAT)		SAT Correct Responses - SAT Errors

		instruction changing<br>at random (i.e., match the figures by<br>shape, or by colour).	

Table S3. Means and standard deviations for the outcome measures across time-points

Outcome Measure	MLC901 (months)					Placebo (months)				
	Baseline N=92	1 N=92	3 N=89	6 N=89	9 N=89	Baseline N=90	1 N=90	3 N=90	6 N=89	9 N=89
**Cognitive Functioning (CNS Vital Signs)***										
Verbal memory (60-120)	88.5 (20.7)	92.3 (20.9)	101.7 (19.9)	108.1 (17.9)	107.1 (20.5)	89.3 (22.3)	93.8 (21.9))	104.1 (17.5)	108.1 (17.9)	107.1 (20.5)
Visual memory (60-120)	95.1 (19.8)	97.2 (20.9)	105.5 (20.0)	109.1 (23.9)	111.0 (24.0)	96.4 (21.9)	99.7 (21.9)	106.6 (22.6)	112.8 (20.2)	112.9 (22.3)
Processing speed (60-120)	84.7 (17.9)	89.8 (18.8)	99.3 (19.4)	104.2 (22.7)	104.1 (19.8)	82.7 (16.3)	90.0 (18.6)	97.9 (19.9)	100.7 (20.9)	102.5 (16.2)
Executive functioning (60-120)	83.1 (20.5)	94.9 18.5)	103.4 (16.6)	105.6 (16.6)	105.7 (16.0)	85.6 (18.1)	95.6 (16.3)	103.4 (14.6)	107.4 (14.9)	106.6 (13.7)
Reaction time (60-120)	76.2(16.8)	77.1(17.4)	83.5(18.7)	83.2 (17.7)	85.3 (18.2)	75.7 (17.7)	79.0 (17.6)	81.9 (16.7)	83.0 (19.0)	84.5 (18.4)
Complex attention (60-120)	81.4 (22.6)	90.6 (23.5)	102.8 (16.7)	103.0 (17.1)	103.9 (17.5)	85.7 (22.2)	90.7 (22.0)	100.8 (15.2)	105.4 (13.6)	105.7 (12.5)
**Cognitive Failures Questionnaire at baseline**										
Total Score (0-100)	47.0 (10.7)					45.3 (8.7)				
**Rivermead Post-Concussion Symptoms Questionnaire**										
RPQ Total Score	26.9 (9.4)	22.7 (9.5)	18.1 (8.7)	15.3 (8.9)	14.0 (9.2)	24.3 (9.6)	23.0 (8.7)	19.2 (9.2)	18.2 (9.6)	16.6 (9.2)
**Quality of Life ***										
QOLIBRI Total Score	61.7 (10.7)	66.1 (10.9)	71.0 (10.9)	72.6 (13.8)	76.5 (11.2)	66.7 (11.5)	66.7 (12.5)	70.3 (11.1)	70.3 (14.2)	75.3 (11.1)
**Mood**										
Anxiety (0-21)	8.0 (3.7)	7.0 (3.3)	5.4 (3.0)	4.3 (2.9)	4.0 (3.1)	7.0 (3.5)	6.9 (3.8)	5.9 (3.1)	5.3 (3.0)	4.4 (2.9)
Depression (0-21)	6.6 (3.4)	6.2 (3.3)	4.6 (2.9)	3.7 (2.9)	3.4 (2.7)	6.4 (3.3)	5.9 (2.9)	5.4 (2.6)	4.7 (2.5)	4.4 (2.6)

*=high scores mean better outcome

Table S4. Rivermead Post-Concussion Questionnaire scores in the MLC901 and Placebo groups at baseline, 3, 6 and 9 months post-randomisation

Parameter	Visit/Score	Placebo (N=90)	MLC901 (N=92)	P-value*
Forgetfulness, poor memory	Baseline			
	0	9 (10.00)	7 (7.61)	
	1	14 (15.56)	8 (8.70)	
	2	28 (31.11)	34 (36.96)	
	3	31 (34.44)	28 (30.43)	
	4	8 (8.89)	15 (16.30)	
	1 month			0.526
	0	10 (11.11)	8 (8.70)	
	1	9 (10.00)	13 (14.13)	
	2	34 (37.78)	28 (30.43)	
	3	33 (36.67)	36 (39.13)	
	4	4 (4.44)	7 (7.61)	
	3 months			0.384
	0	13 (14.44)	7 (7.61)	
	1	14 (15.56)	23 (25.00)	
	2	35 (38.89)	44 (47.83)	
	3	25 (27.78)	15 (16.30)	
	4	3 (3.33)	3 (3.26)	
	6 months			0.008
	0	11 (12.22)	16 (17.39)	
	1	23 (25.56)	32 (34.78)	
	2	30 (33.33)	35 (38.04)	
	3	21 (23.33)	6 (6.52)	
	4	5 (5.56)	3 (3.26)	
	9 months			0.006
	0	15 (16.67)	16 (17.39)	
	1	21 (23.33)	41 (44.57)	
	2	29 (32.22)	26 (28.26)	
	3	21 (23.33)	6 (6.52)	
	4	4 (4.44)	3 (3.26)	
Parameter	Visit/Score	Placebo (N=90)	MLC901 (N=92)	P-value*
Poor Concentration	Baseline			
	0	8 (8.89)	6 (6.52)	
	1	14 (15.56)	10 (10.87)	
	2	22 (24.44)	34 (36.96)	
	3	36 (40.00)	31 (33.70)	
	4	10 (11.11)	11 (11.96)	
	1 month			0.751

	0	7 (7.78)	10 (10.87)	
	1	21 (23.33)	17 (18.48)	
	2	22 (24.44)	27 (29.35)	
	3	34 (37.78)	33 (35.87)	
	4	6 (6.67)	5 (5.43)	
	3 months			0.064
	0	13 (14.44)	9 (9.78)	
	1	19 (21.11)	30 (32.61)	
	2	23 (25.56)	37 (40.22)	
	3	31 (34.44)	14 (15.22)	
	4	4 (4.44)	2 (2.17)	
	6 months			0.002
	0	16 (17.78)	18 (19.57)	
	1	21 (23.33)	35 (38.04)	
	2	19 (21.11)	32 (34.78)	
	3	30 (33.33)	5 (5.43)	
	4	4 (4.44)	2 (2.17)	
	9 months			0.049
	0	17 (18.89)	16 (17.39)	
	1	26 (28.89)	39 (42.39)	
	2	20 (22.22)	29 (31.52)	
	3	23 (25.56)	6 (6.52)	
	4	4 (4.44)	2 (2.17)	
Parameter	Visit/Score	Placebo (N=90)	MLC901 (N=92)	P-value*
Taking Longer to Think	Baseline			
	0	10 (11.11)	6 (6.52)	
	1	14 (15.56)	14 (15.22)	
	2	30 (33.33)	26 (28.26)	
	3	26 (28.89)	37 (40.22)	
	4	10 (11.11)	9 (9.78)	
	1 month			0.762
	0	9 (10.00)	11 (11.96)	
	1	15 (16.67)	17 (18.48)	
	2	34 (37.78)	30 (32.61)	
	3	24 (26.67)	28 (30.43)	
	4	8 (8.89)	6 (6.52)	
	3 months			0.397
	0	13 (14.44)	13 (14.13)	
	1	27 (30.00)	26 (28.26)	
	2	19 (21.11)	35 (38.04)	
	3	26 (28.89)	15 (16.30)	
	4	5 (5.56)	3 (3.26)	
	6 months			0.009
	0	17 (18.89)	21 (22.83)	
	1	20 (22.22)	30 (32.61)	
	2	27 (30.00)	34 (36.96)	
	3	20 (22.22)	5 (5.43)	

	4	6 (6.67)	2 (2.17)	
	9 months			0.093
	0	20 (22.22)	24 (26.09)	
	1	27 (30.00)	30 (32.61)	
	2	18 (20.00)	30 (32.61)	
	3	20 (22.22)	5 (5.43)	
	4	5 (5.56)	3 (3.26)	

*Wilcoxon test

References

1. Theadom A, Barker-Collo S, Jones KM, Parmar P, Bhattacharjee R, Feigin VL. MLC901 (NeuroAiD II) for cognition after traumatic brain injury: a pilot randomized clinical trial. *Eur J Neurol*. 2018;25:1055-e1082. doi: 10.1111/ene.13653
