# Supplementary material for: A double-blind, placebo-controlled, randomized, multi-centre, phase III study of MLC901 (NeuroAiDTMII) for the treatment of cognitive impairment after mild traumatic brain injury
Source: PLoS One. 2025 Jul 10;20(7):e0310229. doi: 10.1371/journal.pone.0310229 (PMC12244563; doi:10.1371/journal.pone.0310229)
Supplement: Attachment — Submitted filename: Samurai Response to PLOS ONE Reviewers.docx [file pone.0310229.s005.docx]

1. Please ensure that your manuscript meets PLOS ONE's style requirements, including those for file naming.

Response: We have thoroughly reviewed the manuscript and made the necessary corrections to make it fully compliant with the PLOS ONE’s style requirements

2. We note that you have indicated that there are restrictions to data sharing for this study. For studies involving human research participant data or other sensitive data, we encourage authors to share de-identified or anonymized data. However, when data cannot be publicly shared for ethical reasons, we allow authors to make their data sets available upon request.

Before we proceed with your manuscript, please address the following prompts:

a) If there are ethical or legal restrictions on sharing a de-identified data set, please explain them in detail (e.g., data contain potentially identifying or sensitive patient information, data are owned by a third-party organization, etc.) and who has imposed them (e.g., a Research Ethics Committee or Institutional Review Board, etc.). Please also provide contact information for a data access committee, ethics committee, or other institutional body to which data requests may be sent.

Response: Done

b) If there are no restrictions, please upload the minimal anonymized data set necessary to replicate your study findings to a stable, public repository and provide us with the relevant URLs, DOIs, or accession numbers. Please see http://www.bmj.com/content/340/bmj.c181.long for guidelines on how to de-identify and prepare clinical data for publication. For a list of recommended repositories, please see https://journals.plos.org/plosone/s/recommended-repositories. You also have the option of uploading the data as Supporting Information files, but we would recommend depositing data directly to a data repository if possible.

Please update your Data Availability statement in the submission form accordingly.

Response: The study sponsor (Moleac Pte Ltd) is committed to provide the study datasets upon written request to Dr Sylvain Durrleman at <sylvain.durrleman@moleac.com>. Should the reasonable request for data sharing be raised, data access to all data used by the authors of this article will be provided without restriction.

3. Please ensure that you refer to Figure 2, 3, 4, and 5 in your text as, if accepted, production will need this reference to link the reader to the figure.

Response: the text has been revised to ensure that unequivocal reference to each of the tables and figures is made in the manuscript

5. Review Comments to the Author

Please use the space provided to explain your answers to the questions above. You may also include additional comments for the author, including concerns about dual publication, research ethics, or publication ethics. (Please upload your review as an attachment if it exceeds 20,000 characters)

Response: all questions above have been addressed in the revised manuscript.

**Reviewer #1:** As the statistical reviewer I will focus on methods and reporting. This is generally and well reported study. The main model is appropriate.

**Major**

1) the power calculation should be presented before the "study participants" section.

Response: the order of the sections has now been revised accordingly. The statistical analyses and power calculation” section has now been split in two sections : “Power calculation” , which is positioned before the “Study participants” section and “Statistical analyses” section, which is the last paragraph of the “METHODS” chapter.

2) in the methods section clarify which time points were assessed - was there a primary endpoint? 3 months, 6 months? or 9 months. Also how was that factored into the mixed effects model? a separate model for each endpoint, controlled for baseline (arguably the clearest way to analyse and present results from). Alternatively, you can have a repeated measures design, which is not what you have (or did not describe as you should have) which may give an "overall" effect across all endpoints.

Response: The primary timepoint was the 6-month assessment for each of the outcomes, corresponding to the end-of-treatment visit. This is clearly stated in the manuscript (line 120). Separate mixed models were constructed for each of the outcome parameters and their dependent variable was the respective outcome parameter change from baseline. The analysis used a repeated measure design with all post baseline timepoints used as repeated effect in each model, while treatment group and its interaction with timepoints along with parameter baseline value, participant age, gender, time since injury (dichotomized into two groups: 1-3 months/4-12 months) and study centers were included as fixed effects in each model. Estimates obtained from the model (Least Squares Means) for the parameters change from baseline at month 6 (primary timepoint) and month 9 were provided with their associated 95%CI and p-values to conclude about treatment efficacy. We added this explanation in the Statistical analysis section of the revised manuscript.

3) I couldn't replicate the power calculations and I followed the reference the authors provide but I could not tally what was said there to what the authors say (in terms of the SD for example). Nevertheless, the power calculations are correct (on the assumptions provided) but they tend to ignore the potential intraclass correlation coefficient since across many centers. So the authors need to discuss this as a potential problem when it comes to power and also confirm in their response where the information on the assumptions was in

the cited paper.

Response: the sample size calculation for the trial was based upon the results obtained in the pilot study done in New Zealand by Theadom et al. (Ref. 16). In this publication, the following data were provided in Supplementary Table 1

**Complex Attention score : Mean (SD**)

|  | **MLC-901 (n=36)** | **Placebo (n=42)** |
| --- | --- | --- |
| **Baseline** | 83.86 (22.06) | 85.43 (16.52) |
| **Month 6** | 94.18 (15.29) | 82.31 (21.56) |

*(extract from the Supplementary Table 1 in the publication*)

As can be seen in the table above, the SD for the score at M6 is in the range 15-21, so the SD for the change from baseline at M6 is likely to be < 20.

However, in view of some uncertainty about the variability of the measure, and in order to be conservative and achieve sufficient power for the trial, we designed the study protocol using a SD of 20.

As pointed out by the Reviewer, the intraclass correlation for centers was not taken into account in the power calculation, as there was limited information to use as to which magnitude of correlation to consider in the calculation. In the final analysis, however, the intraclass correlation for centers is accounted for, via including study centers as fixed effect in the model.

It can be noted also that the withdrawal rate of 30% planned for in the power calculation turned out to be largely overestimated, resulting in a power for the trial higher than the originally planned 80%. We added this information to the Supplementary materials (page 3).

**Minor**

1) multivariable not multivariate (implies multiple outcomes).

Response: we thank the reviewer for the remark and will make the modifications

2) methods section "tests of difference" is not a clear enough statement. State the exact tests used for each variable type.

Response: we have clarified in the manuscript that t-tests and Chi-2 tests were used for the quantitative and the categorical variables, respectively.

3) the authors say "Model selection was undertaken with each outcome using standard selection heuristics. Covariates were selected based on improving the overall efficiency of the model. Regardless, baseline value of the outcome variable, age, gender, time since injury (1-3 months/4-12 months) and study center were included as covariates in the mixed effects model". The authors need to clarify the model building more clearly in terms of the covariate selection process and explain better how they arrived at the final model.

Response: we thank the reviewer for the remark, and we have simplified the section to make it more precise, as in fact the “heuristic” covariate selection model was more straightforward, and simply included in the model the clinically important variables as covariates. Indeed, as explained in Response to question 2 and stated in the revised manuscript (lines 221-227), the model that was used is a repeated measure design with all post baseline timepoints used as repeated effect, while treatment group and its interaction with timepoints along with parameter baseline value, participant age, gender, time since injury (1-3 months/4-12 months) and study centers were included as fixed effects in each model.

**Reviewer #2:** Thank you for the opportunity to review this manuscript. Overall, the study is well-designed and addresses an important clinical question regarding the efficacy and safety of MLC901 (NeuroAiD II) for treating cognitive impairment following mild traumatic brain injury (TBI).

**Strengths:**

The randomized, double-blind, placebo-controlled, multi-center design is a significant strength and adds considerable value to the research. This approach represents the gold standard for clinical trials, ensuring high internal validity. The use of stratified permuted block randomization and intention-to-treat analysis further enhances the reliability of the results.

The study’s comprehensive evaluation, which includes both primary and secondary outcomes—cognitive functioning, post-concussion symptoms (RPQ), quality of life (QOLIBRI), and the Hospital Anxiety and Depression Scale (HADS)—provides a thorough assessment of the intervention’s effects.

Additionally, the detailed reporting of adverse events contributes to the evaluation of the treatment's safety profile.

Achieving a 98% retention rate at the 9-month follow-up is commendable for a study of this length and reinforces the credibility of the data.

Although the primary outcome did not reach statistical significance, the clinically and statistically significant improvements observed in secondary measures—such as post-concussion symptoms, quality of life, anxiety, and depression—are noteworthy and provide valuable insights.

Response: we thank the reviewer for these comments and the recognition of the study’s strengths.

**Areas for Improvement:**

The lack of significant findings for the primary outcome (complex attention) may raise some concerns. Strengthening the discussion by exploring potential reasons why the intervention did not impact primary cognitive outcomes would be beneficial. It might also help to consider methodological challenges, such as issues with implementing the CNS Vital Signs tool in the Russian context.

Response: we have emphasized in the discussion the methodological challenges and some limitations of the computerized assessment of CNS-VS of Complex Attention and other CNS-VS cognitive parameters.

Since the CNS Vital Signs tool may not have been fully adapted for use in Russia, this could have influenced the results. It would be helpful to address how cultural and linguistic factors were considered and how they might be mitigated in future research.

Response: Indeed, the tool was not fully validated in its Russian translation, and some limitations to travel to Russia, some restrictions due to Covid-19 pandemia leading to training sessions conducted only via virtual meetings, limited investigators meetings, etc. The limited digital literacy of some patients in Russia has also led to suboptimal use of the computerized assessment tool. These methodological challenges and some limitations of the computerized assessment of CNS-VS of Complex Attention and other CNS-VS cognitive parameters in Russia (including cultural and linguistic factors) may have influenced the negative results of these outcome measures in the trial. We added these statements to the Discussion section of the revised manuscript.

The study's population is limited to Russian participants, which may restrict its generalizability. Future studies could benefit from including a more diverse sample to broaden the applicability of the findings.

Response: We add this point as a limitation in the Discussion section, recognizing that, although being a large and multicenter trial, the SAMURAI study is conducted in only one country.

The data availability statement mentions that some restrictions will apply. For a journal like PLOS ONE, a more open data-sharing policy is typically encouraged. If full data sharing is not possible, a clearer explanation of the restrictions would be beneficial.

Response: The study sponsor (Moleac Pte Ltd) is committed to provide the study datasets upon written request to Dr Sylvain Durrleman at <sylvain.durrleman@moleac.com>. Should the reasonable request for data sharing be raised, data access to all data used by the authors of this article will be provided without restriction.

The interpretation of the results, particularly the significant improvements in secondary outcomes, could be expanded to provide greater insight into the clinical relevance of MLC901. Even if its cognitive benefits were not demonstrated, the improvement in quality of life and other outcomes is important.

Response: We have made modifications in the manuscript to further discuss these important endpoints and further emphasize their importance. Specifically, we stated that the significant improvements in non-cognitive secondary outcomes up to 9 months post-randomisation are of considerable clinical significance because up to 20% of people with mild TBI show persistent post-concussion symptoms, about one third of people with mild TBI experience reduced quality of life, and many of them suffer from anxiety or depression.

The manuscript could also benefit from some reorganization to enhance clarity, particularly in the results and discussion sections. A more concise presentation of tables and figure legends would help readers better understand the data.

Response: In the revised manuscript, we re-organized the Methods section, revised the Discussion section, and amended Tables 1-2, and Figures 2-6.

While the discussion covers relevant background information, it would be strengthened by a more thorough comparison with existing literature, particularly other studies on MLC901 and treatments for cognitive impairments following TBI.

Response: Unfortunately, apart from the current trial and pilot trial in New Zealand we referred to, there was no other RCTs evaluating treatment effects of MLC901 on cognitive impairments following TBI.

Lastly, expanding the discussion of limitations and offering more concrete suggestions for future research would enhance the paper. For example, recommendations for adapting cognitive testing tools to different cultural contexts and exploring the long-term effects of MLC901 on non-cognitive outcomes would be valuable additions.

Response: We thank the reviewer for this valuable suggestion. In the revised manuscript, we added to the last paragraph of Discussion that adapting cognitive testing tools to different cultural contexts and exploring the long-term effects of MLC901 on non-cognitive outcomes are also warranted.

While revising your submission, please upload your figure files to the Preflight Analysis and Conversion Engine (PACE) digital diagnostic tool, https://pacev2.apexcovantage.com/. PACE helps ensure that figures meet PLOS requirements. To use PACE, you must first register as a user. Registration is free. Then, login and navigate to the UPLOAD tab, where you will find detailed instructions on how to use the tool. If you encounter any issues or have any questions when using PACE, please email PLOS at [figures@plos.org](mailto:figures@plos.org). Please note that Supporting Information files do not need this step.

Response: Done.
